# Genome Sequence of *Azospirillum brasilense* CBG497 and Comparative Analyses of *Azospirillum* Core and Accessory Genomes provide Insight into Niche Adaptation

**DOI:** 10.3390/genes3040576

**Published:** 2012-09-28

**Authors:** Florence Wisniewski-Dyé, Luis Lozano, Erika Acosta-Cruz, Stéphanie Borland, Benoît Drogue, Claire Prigent-Combaret, Zoé Rouy, Valérie Barbe, Alberto Mendoza Herrera, Victor González, Patrick Mavingui

**Affiliations:** 1 Université de Lyon, UMR 5557 CNRS, USC 1193 INRA, VetAgro Sup Ecologie Microbienne, Villeurbanne 69622, France; E-Mails: eyacosta@yahoo.com (E.A.-C.); stephanie.borland@hotmail.fr (S.B.); benoit.drogue@gmail.com (B.D.); claire.prigent-combaret@univ-lyon1.fr (C.P.-C.); patrick.mavingui@univ-lyon1.fr (P.M.); 2 Centro de Ciencias Genómicas, Universidad Nacional Autónoma de México, AP565-A Cuernavaca, Morelos 62210, México; E-Mails: llozano@ccg.unam.mx (L.L.); vgonzal@ccg.unam.mx (V.G.);; 3 Laboratoire d’Analyse Bioinformatique en Génomique et Métabolisme CNRS UMR8030, France; E-Mail: zrouy@genoscope.cns.fr; 4 Institut de Génomique, CEA, Génoscope, 2 rue Gaston Crémieux, 91057 Evry, France; E-Mail: vbarbe@genoscope.cns.fr; 5 Centro de Biotecnología Genómica, Instituto politécnico Nacional, 88710 Reynosa, Tamaulipas, México; E-Mail: amendozah@ipn.mx

**Keywords:** *Azospirillum*, core genome, chromid, horizontal gene transfer, orthologous groups, rhizosphere

## Abstract

Bacteria of the genus *Azospirillum* colonize roots of important cereals and grasses, and promote plant growth by several mechanisms, notably phytohormone synthesis. The genomes of several *Azospirillum* strains belonging to different species, isolated from various host plants and locations, were recently sequenced and published. In this study, an additional genome of an *A. brasilense* strain, isolated from maize grown on an alkaline soil in the northeast of Mexico, strain CBG497, was obtained. Comparative genomic analyses were performed on this new genome and three other genomes (*A. brasilense* Sp245, *A. lipoferum* 4B and *Azospirillum* sp. B510). The *Azospirillum* core genome was established and consists of 2,328 proteins, representing between 30% to 38% of the total encoded proteins within a genome. It is mainly chromosomally-encoded and contains 74% of genes of ancestral origin shared with some aquatic relatives. The non-ancestral part of the core genome is enriched in genes involved in signal transduction, in transport and in metabolism of carbohydrates and amino-acids, and in surface properties features linked to adaptation in fluctuating environments, such as soil and rhizosphere. Many genes involved in colonization of plant roots, plant-growth promotion (such as those involved in phytohormone biosynthesis), and properties involved in rhizosphere adaptation (such as catabolism of phenolic compounds, uptake of iron) are restricted to a particular strain and/or species, strongly suggesting niche-specific adaptation.

## 1. Introduction

Bacteria of the genus *Azospirillum* colonize roots of important cereals and grasses, and promote plant growth by several mechanisms, notably phytohormone synthesis [1,2]. Besides their potential as biofertilizer, some strains can also benefit plant health through biological control of phytoparasitic plants [3] or bacterial pathogens [4], or by inducing disease resistance [5]. In addition, *Azospirillum* may have applications in bioremediation of wastewater as it can increase the growth of microalgae commonly used in this process, such as *Chlorella* [6]. In order to rationalize the use of *Azospirillum*, genetics studies on amenable strains have mainly focused on genes involved in nitrogen fixation, auxin synthesis and on properties linked to survival in the rhizosphere [1,2,7]; knowledge of the gene repertoire of several strains may provide new insights into the *Azospirillum-*plant association. 

Pioneer studies have shown that genomes of *Azospirillum* are constituted of multiple replicons and their size varies among species from 4.8 Mbp to 9.7 Mpb [8,9]. Recently, the genomes of four strains belonging to different species, isolated from various host plants and locations, were sequenced and published. The genome of *Azospirillum* sp. B510, a strain isolated from disinfected rice stems in Japan, has a size of 7.6 Mbp and consists of a single chromosome (3.31 Mbp) and six plasmids [10]. The genome of *Azospirillum amazonense* Y2, a strain isolated from the gramineous *Hyparrhenia rufa* in Brazil, was reported earlier to be constituted of four replicons of 2.7 Mbp, 2.2 Mbp, 1.7 Mbp and 0.75 Mbp [9] and its draft sequence composed of 1,617 contigs was published recently [11]. Genomes of *Azospirillum lipoferum* 4B, a strain isolated from rice in France, and *Azospirillum brasilense* Sp245, a strain isolated from wheat in Brazil, both carry seven replicons and display genome sizes of respectively 6.8 and 7.5 Mbp [12]. Whereas the largest replicon has all the features of a bacterial chromosome, several replicons could be defined as chromids [13] and some strains of *Azospirillum* appear to possess the largest number of chromids among all prokaryotic genomes sequenced to date [12]. Moreover, very little synteny was found between replicons of *Azospirillum* strains and more genomic rearrangements could be pinpointed in *Azospirillum* genomes compared to rhizobial genomes known for their genome plasticity [12]. This extraordinary genome plasticity was previously described experimentally in *Azospirillum*: indeed, the appearance of phenotypic variants was correlated with plasmid loss or reorganization [14] and the presence of bacteriophages was evidenced [15].

Interestingly, among the family *Rhodospirillaceae*, members of the genus *Azospirillum* have the particularity of being terrestrial and plant-associated whereas nearly all known representatives live in aquatic habitats. By using a robust scheme for detecting ancestral and horizontally transferred genes in *Azospirillum*, it was shown that nearly half of the genes whose origins could be resolved, appear to be horizontally transferred from soil and plant-associated bacteria; not surprisingly the majority of genes encoding functions critical for survival in the rhizosphere and association with plants are among those acquired by horizontal gene transfer (HGT) [12]. Moreover, separation of *Azospirillum* from their close aquatic relatives approximately coincided with the emergence of vascular plants on land [12].

In the present study, the genome sequence of another member of this genus, *A. brasilense* CBG497, a strain isolated from maize grown on an alkaline soil (pH 8) in the northeast of Mexico, was first obtained. The choice was made on this strain as it is able to stimulate maize biomass yield under greenhouse conditions [16], and was recently developed as a commercial biofertilizer [17]. Then, comparative genomics analyses were performed on the four available whole genomes in order to define the *Azospirillum* core genome. The following questions were addressed: Does the core genome contain mainly genes of ancestral origin shared with aquatic relatives? Does the core genome contain genes putatively involved in rhizosphere adaptation and interaction with plants? Which functions are specific to a strain or to a species?

## 2. Results and Discussion

### 2.1. Genomic Features of *Azospirillum* Genomes

When *A. brasilense* CBG497 was subjected to replicon analysis by the plasmid Eckhardt method, five plasmids could be evidenced [18]. Pulse-field gel electrophoresis analysis confirmed the presence of five plasmids, with estimated sizes of 1.8 Mbp, 0.73Mbp, 0.65 Mbp, 0.60 Mbp and 0.15 Mbp (data not shown). The whole genome sequence of *A. brasilense* CBG497 was obtained by the 454 pyrosequencing technology and after assembly a total of 156 contigs was obtained and clustered into six scaffolds corresponding to the six expected replicons. It comprises a chromosome of 2.9 Mbp and plasmids of 1.6 Mbp, 0.731 Mbp, 0.488 Mbp, 0.606 Mbp and 0.149 Mbp that correspond respectively to p1, p2, p3, p4 and p6 of *A. brasilense* Sp245. Thus, the same nomenclature as the one used for *A. brasilense* Sp245 was applied to designate these plasmids. According to PFGE data, only the sequence of p3 seems incomplete (estimated size of 650 kb *versus* a sequenced size of 488 kb); consequently, genome coverage is estimated at 97%–98%.

General genomic features of *A. brasilense* CBG497 and of the three other *Azospirillum* strains used in this study are presented in Table 1. Genome size ranges from 6.5 Mbp (*A. brasilense* CBG497) to 7.6 Mbp (*Azospirillum* sp. B510). All strains are composed of seven replicons except *A. brasilense* CBG497 which contains only six. For all strains, only the biggest replicon has a typical chromosomal OriC replication origin whereas all the other replicons have repABC/parAB plasmid-type replication systems. Some of the latters have been classified as chromids [12,13]; p1, p2 and p4 of *A. brasilense* CBG497 also fulfill the chromid criteria as they contain respectively 40, 6 and 9 of the essential core genes that are found on the chromosome in other species [13,18]. The smallest replicon, p6, is a typical plasmid for all strains studied and displays the lowest of the averaged GC content, which suggests an external origin by HGT; such an observation was previously made for *Rhizobium etli* and *Rhizobium leguminosarum* [19]. 

Chromids and plasmids comprise the largest proportion of the total genome, with 55.2% for *A. brasilense* CBG497, 56.4% for *A. lipoferum* 4B and *Azospirillum* sp. B510, and 59.8% for *A. brasilense* Sp245. So, in addition to possessing the largest number of chromids among all prokaryotic genomes sequenced to date, *Azospirillum* has the biggest proportion of its genome on non-chromosomal replicons. 

**Table 1:** Genomic features of *Azospirillum* strains used in this study.

Strain and features	Chromosome	p1	p2	p3	p4	p5	p6	TOTAL
***A. brasilense*** ** CBG497**						Absent		
size of replicon^§^	2,900,071	**1,598,241**	**731,389**	488,405	**606,415**		148,687	6,473,208
G+C content	68.4	68.8	68.8	66.05	69.3		67.1	68.4
number of ORFs	2895	1430	643	512	583		122	6185
rRNA	nk	1	1	nk	nk		0	nk
tRNA	42	16	1	2	6		0	67
***A. brasilense*** ** Sp245**								
size of replicon	3,023,440	**1,766,028**	**912,449**	778,798	**690,334**	191,828	167,364	7,530,241
G+C content	68.6	68.6	68.3	68.2	69	66.7	66.8	68.5
number of ORFs	3309	1812	922	824	691	163	125	7846
rRNA	2	3	2	0	1	0	0	8
tRNA	44	25	2	0	9	0	1	81
***A. lipoferum*** ** 4B size of replicon**								
G+C content	2,988,332	**1,040,425**	**750,123**	**648,491**	**645,253**	**478,032**	295,744	6,846,400
number of ORFs	67.6	67.6	67.6	67.8	68.3	67.7	67.1	67.7
rRNA	2904	883	640	555	599	415	237	6233
tRNA	2	3*	2	1	0	1^†^	0	9
	46	12	5	2	6	8	0	79
***Azospirillum sp.*** **B510**								
size of replicon	3,311,395	**1,455,109**	**723,779**	**681,723**	**628,837**	**537,299**	261,596	7,599,738
G+C content	67.8	67.6	67.5	67.4	68	67.5	65.9	67.6
number of ORFs^#^	3287	1263	693	589	598	464	232	7126
rRNA	2	4	1	1	0	1	0	9
tRNA	45	14	2	3	6	9	0	79

**^§ ^**When the size is indicated in bold, the chromid definition applies to the corresponding replicon, *i.e.* plasmid-type maintenance replication systems, presence of essential genes and a nucleotide composition close to that of the chromosome [12,13]; **^¶ ^**The third criteria of chromid definition (nucleotide composition close to that of the chromosome) does not apply for these two replicons; * The 5S rRNA is missing from one of the operons; **^†^** The 23S rRNA is absent;**^ #^** The number of ORFs corresponds to the one established after the sequence was imported and annotated into the MaGe platform [78]; nk: not known.

### 2.2. Genomic Relatedness between *Azospirillum* Strains

Before undertaking comparative genomic analysis, the relatedness between *Azospirillum* sp. B510 and the other *Azospirillum* strains used in this study was clarified. *Azospirillum* sp. B510 was previously shown to be related to the species *A.lipoferum* to which it was originally affiliated [20]. Subsequent studies revealed that it was closer to the species *A. oryzae* than to the species *A. lipoferum* [10]. Since new species of *Azospirillum * have recently been described, a 16S rRNA phylogenetic tree was constructed, and this confirmed that strain B510 was more closely related to the species *A. oryzae* and *A. zeae* (Supplementary Figure S1). Recently, the average nucleotide identity was determined between *A. lipoferum* 4B and *Azospirillum* sp. B510, and was found to be 91% [12] supporting the fact that these two strains belong to different species [21].

**Figure 1:**
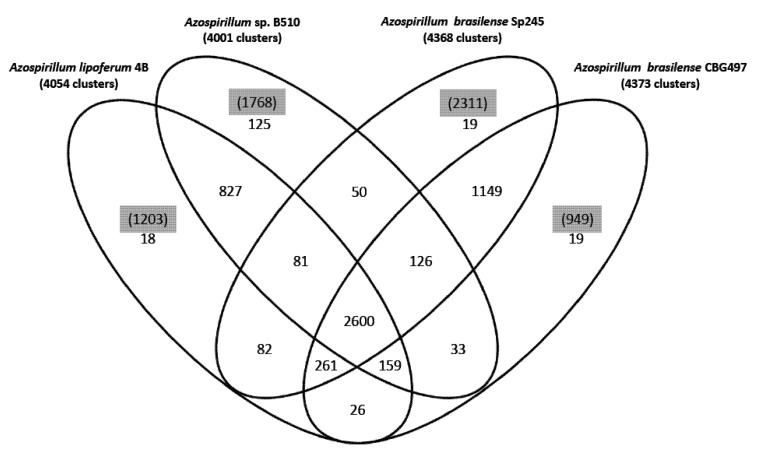
Venn diagram showing the distribution of protein families in the genomes of *A. brasilense* CBG497, *A. brasilense* Sp245, *A. lipoferum* 4B, *Azospirillum* sp. B510. Numbers in black indicate the number of protein families; numbers in parenthesis and highlighted in grey refer to the number of unique proteins in each genome that do not fall in any family.

**Figure 2:**
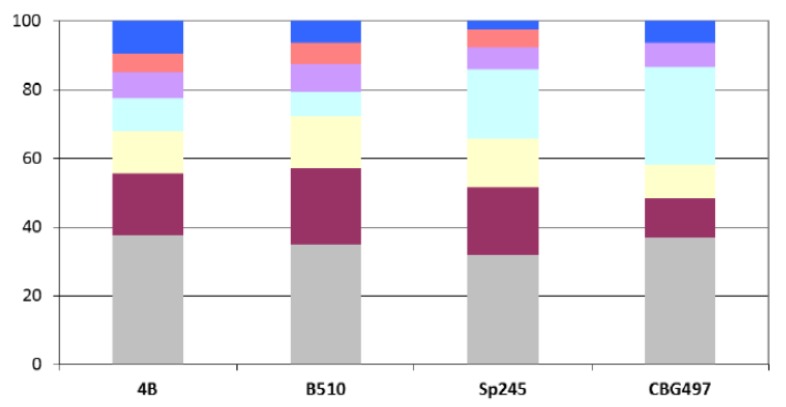
Repartition among replicons of *Azospirillum* unique proteins. Color legend: Grey (chromosome), burgundy (p1), yellow (p2), light blue (p3), purple (p4), orange (p5), dark blue (p6).

A first comparative analysis was undertaken in order to classify proteins into families and hence to evaluate the genomic relatedness among *Azospirillum* strains in terms of protein coding content. As the draft sequence of *A. amazonense* Y2 is composed of 1,617 contigs with only 3,319 predicted CDS, far fewer than what is expected for its 7.3 Mbp genome-size, it was excluded for the comparative analysis. The predicted proteins of the four remaining *Azospirillum* genome sequences (a total of 27,400 proteins) were clustered using the MCL algorithm [22]. About 47% of the protein families identified (2,600 out of 5,575) are shared by the four strains (Figure 1); the two *A. brasilense* strains share 74% of the protein families (4,136 out of 5,575) whereas the *A. lipoferum* and *Azospirillum* sp. pair share 66% of the protein families (3,667 out of 5,575). Other combinations share between 51.2% and 54.6% of the protein families; these results are in accordance with *A. lipoferum* 4B and *Azospirillum* sp. B510 belonging to different species but to species more closely related than the *A. brasilense* and *A. lipoferum* species. A number of protein families are exclusive to individual genomes (Figure 1). As for proteins that do not appear in any protein families, their number is quite variable from one strain to another (949 for *A. brasilense* CBG497; 2,311 for *A. brasilense* Sp245; 1,203 for *A. lipoferum* 4B; 1,768 for *Azospirillum* sp. B510) and not surprisingly is in direct correlation with genome size. Repartition of these unique proteins among replicons shows that the biggest proportion (>60%) is located outside the chromosome, and that the repartition is variable from one strain to another (Figure 2). For each genome, the array of unique proteins contain approximately 20% of ancestral proteins, 30 to 43% of proteins previously classified as horizontally acquired and the rest being classified as unassigned [12]; this last category consists of proteins having no orthologues in the nr database of Genbank. Some of the unique proteins with assigned functions will be discussed in subsequent sections. 

### 2.3. The *Azospirillum* Core Genome

**Figure 3:**
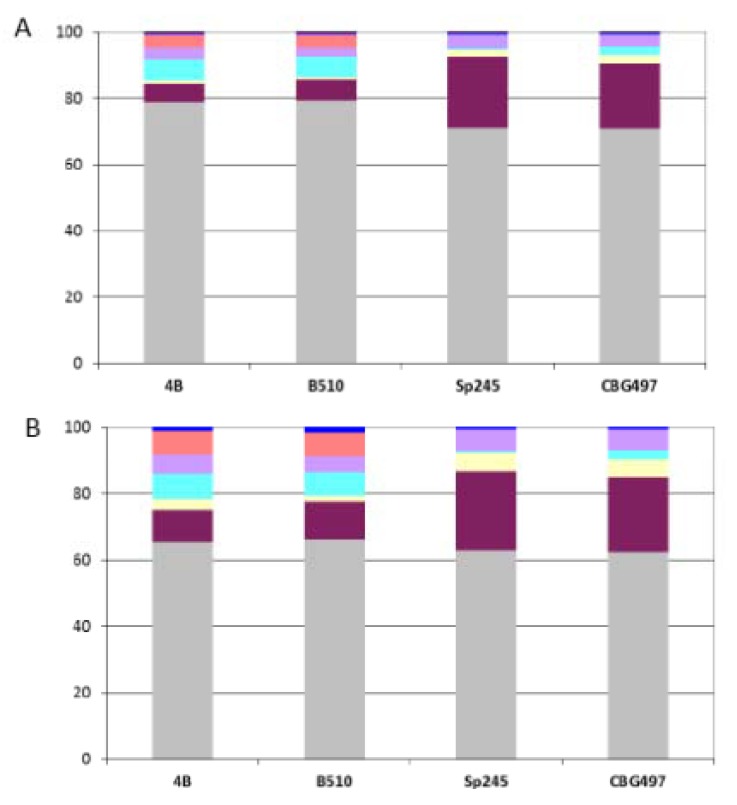
Repartition of orthologous groups among replicons for each *Azospirillum* strain. (A) Repartition of the 1,151 *Azospirillum*-*Rhodospirillum* orthologous groups (ANC-core). (B) Repartition of the 2,328 *Azospirillum* orthologous groups (AZO-core). Color legend: Grey (chromosome), burgundy (p1), yellow (p2), light blue (p3), purple (p4), orange (p5), dark blue (p6).

The most probable set of orthologous proteins shared by the four *Azospirillum* strains and by the phylogenetically related *Rhodospirillum centenum* SW strain was identified by a reciprocal best blast hit criterion. *Rhodospirillum rubrum* was not included in the analysis as this strain is more related to the genus *Magnetospirillum* than to *Azospirillum* (data not shown). A total of 1,151 proteins is shared by these five strains (Supplementary Table S1); this minimal gene set can be considered as the “ancestral” core genome (designated ANC-core) as it contains nearly exclusively (95%) proteins previously classified as ancestral using the scheme developed previously [12]. As expected, the ANC-core is largely encoded by the chromosome and by p1 (from 85% to 90%) in the four *Azospirillum* strains (Figure 3A).

The same procedure was applied to identify the *Azospirillum* core genome (designated AZO-core) *i.e.*, the set of orthologous proteins shared by the four *Azospirillum* strains. A total of 2,328 proteins (including the 1,151 of the ANC-core) is shared by the four strains, representing between 30% to 38% of the total encoded proteins within a genome (Supplementary Table S1). A similar study undertaken on four other *Rhodospirillaceae* belonging to the *Magnetospirillum* genus estimated the magnetobacterial core genome at about 891 genes, which represents 18 to 24% of the total proteins encoded by those genomes [23]. Three different species of *Streptococcus* were shown to share around half of their genes [24].

The AZO-core set is also dominated by proteins of ancestral origin (74%) but contains more than a fifth (22%) of proteins encoded by horizontally acquired genes and a small proportion of proteins encoded by genes whose origin could not be resolved (4%). The repartition among replicons shows that AZO-core is mainly chromosomally-encoded (from 62% to 65% according to the strain considered) (Figure 3B). The non-chromosomal proportion of AZO-core is unevenly distributed among strains. There is a strong dominance of p1 in the *A. brasilense* strains (p1 > p4 > p2), which might be attributable to the size of this replicon; genes of the AZO-core that are p1-encoded in *A. brasilense* strains are found on the chromosome in the two other strains. For *A. lipoferum* and *Azospirillum* sp. strains, the non-chromosomal proportion of AZO-core is mainly found on p1, p3 and p5 (Figure 3B); orthologues of those p3- and p5-encoded genes are scattered on the different replicons bearing AZO-core genes in *A. brasilense* (*i.e.*, chromosome, p1, p2 and p4) (data not shown). So, it appears that p3, p5 (absent in CBG497) and p6 are accessory replicons for *A. brasilense*, which is in accordance with their plasmid (versus chromid) status. For *A. lipoferum* 4B and *Azospirillum* sp. B510, the unique defined plasmid p6, appears to be a dispensable replicon. The p2 chromid of those two strains bears very few AZO-core genes, which is in accordance with the very small number of house-keeping genes previously identified on this replicon [12] and with the observation of p2 loss at high frequency in *A. lipoferum* 4B [14]. 

**Figure 4:**
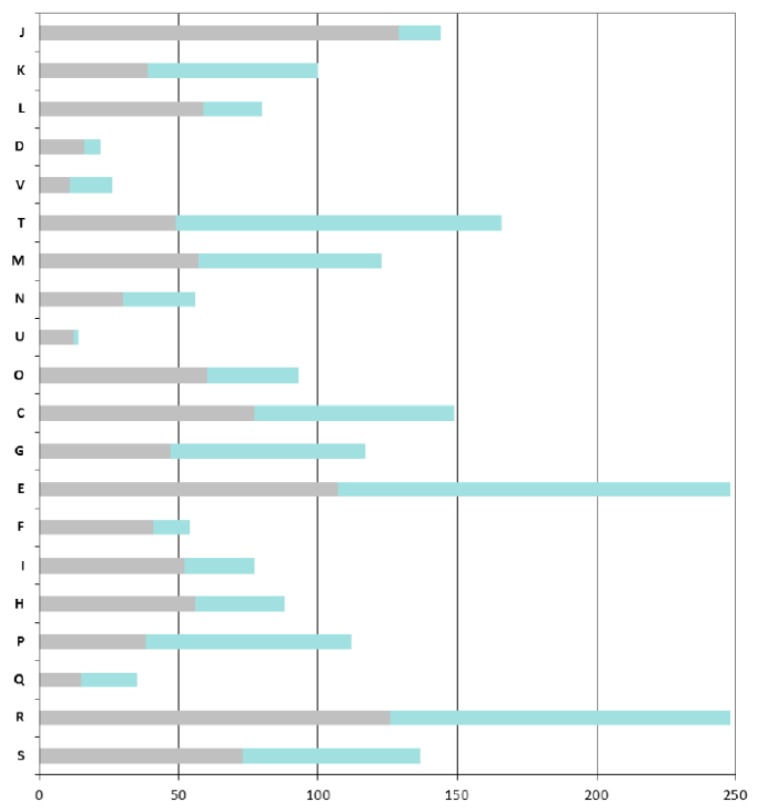
COGs functional classification of the *Azospirillum* orthologous groups. Bars indicate the numbers of orthologous groups for each COG retrieved from the MaGe platform for the *A. lipoferum* 4B orthologues. For each bar, the grey part represents the number of orthologous groups found in the ancestral (*Azospirillum-Rhodospirillum)* orthologous groups, the green part represents the number of orthologous groups that are unique to the *Azospirillum* genus. COG: J, Translation, ribosomal structure and biogenesis; K, Transcription; L, Replication, recombination and repair; D, Cell cycle control; V, Defence mechanisms; T, Signal transduction mechanisms; M, Cell wall, membrane envelope biogenesis; N, Cell motility; U, Intracellular trafficking and secretion; O, Postranslational modification and chaperones; C, Energy production and conversion; E, Amino acid transport and metabolism; F, Nucleotide transport and metabolism; G, Carbohydrate transport and metabolism; H, Lipid transport and metabolism; I, Coenzyme transport and metabolism; P, inorganic ion transport and metabolism; Q, Secondary metabolites biosynthesis, transport and catabolism; R, General function prediction; and S, Function unknown.

As expected, the COGs that are overrepresented in the ANC-core are mainly those involved in “house-keeping” functions: COGs J (Translation, ribosomal structure and biogenesis), C (Energy production and conversion), O (Post-translational modifications, protein turnover, chaperones), M (Cell wall / membrane / envelope biogenesis), E (Amino acid transport and metabolism) and H (Coenzyme transport and metabolism) (Figure 4). 

A particular focus on genes found in the AZO-core that are absent in the ANC-core shows that *Azospirillum* is more adapted to life in fluctuating environments; indeed the AZO-core contains many additional genes involved in signal transduction (COG T, such as two-component histidine kinases, diguanylate cyclases, methyl-accepting chemotaxis receptors) and regulation of transcription (COG K); around 8% of the additional genes encode regulators of two-component systems, transcriptional regulators (mainly LysR, GntR and LuxR-FixJ families) and sigma factors (Figure 4). Adaptation to the rhizosphere is also illustrated by the enrichment of COG G and more importantly COG E, corresponding respectively to “carbohydrate transport and metabolism” and to “amino acid transport and metabolism” (this latter category also includes metabolism of organic acids, compounds that are abundant in the rhizosphere). *Azospirillum* has acquired numerous transport systems (representing 18% of the genes that are specific to the AZO-core); those transporters, mainly ABC transporters (scattered through COGs E, G, and I), can serve to internalize the wide diversity of organic and mineral compounds present in the rhizosphere (notably organic compounds exuded by plant roots) or to expel putative plant toxic compounds via MDR efflux pumps. Genes likely involved in bacterial surface properties are also enriched (COG M), such as those involved in the processing of complex sugars (LPS, EPS), increasing the ability of *Azospirillum* to attach to roots. Genes involved in direct plant-growth promotion do not belong to the AZO-core and seem to have been gained specifically after speciation events or by individual strains (see below). One exception is the PQQ operon, allowing the synthesis of the cofactor pyrroloquinoline quinone, a compound displaying plant growth-promoting properties [25].

**Figure 5:**
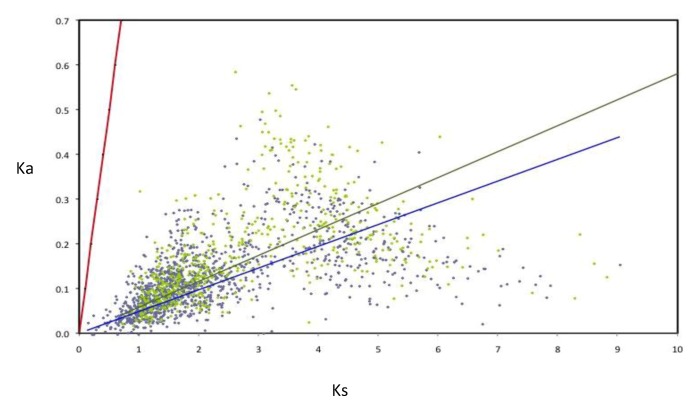
Rates of synonymous (Ks) and non-synonymous substitutions (Ka) in orthologous genes of *Azospirillum*. Linear regressions for chromosomal orthologous genes (blue line and diamonds) and non-chromosomal orthologous genes (green line and diamonds) are indicated. As neutrality (red line) assumes equal nucleotide substitution rates per synonymous and non-synonymous sites, points under the neutrality line indicate negative selection. Strong selective constraints are acting on chromosomal genes (R2 = 0.127) but are slightly less intense for non-chromosomal genes (R2 = 0.092) as can be seen by the dispersion of the green diamonds.

The rates of evolution were evaluated for the components of the AZO-core; in order to perform this, the rates of nucleotide substitution per synonymous (Ks) and non-synonymous (Ka) were calculated for a subset of 1,807 AZO-core genes (for those being chromosomal in all strains and those being non-chromosomal in all strains). All the orthologous groups are under negative selection (Figure 5). Nevertheless many non-chromosomal genes show higher Ka and Ks values than the chromosomal genes, as illustrated by the slopes of the regression lines, suggesting that negative selection is less constrained for genes outside the chromosome. Such an observation was previously made with the *Rhizobium* core genome [19].

### 2.4. Strain-specific Genes Involved in the Colonisation of Plant Roots

The focus was then put on genes with assigned functions that are restricted to a unique strain or to a subgroup of *Azospirillum* strains. A part of the corresponding genes falls into unique protein families described earlier (see section 2.2 and Figure 1) and have mainly been acquired by horizontal gene transfer. Their relevance to interaction with plants and adaptation to the rhizosphere is discussed.

Chemotaxis and motility are primordial for the initiation of root colonization in a wide range of rhizobacteria. The chemotaxis system integrates environmental signals into an appropriate bacterial response by using a dedicated signal transduction pathway. Whereas the AZO-core contains genes implicated in flagellum biosynthesis and genes belonging to the four common chemotaxis operons, specific genes confirm the presence of one and two additional operons respectively in *A. lipoferum* 4B and in *Azospirillum* sp. B510 [12]. Consequently, those two strains, and notably *Azospirillum* sp. B510, contain a significant number of unique genes encoding methyl-accepting proteins, able to detect various physicochemical cues and to relay information to the flagellar motors via a signal transduction cascade.

Interactions between plant and bacterial polysaccharides are thought to mediate bacterial aggregation and attachment processes [26,27]. The AZO-core contains genes whose involvement in biosynthesis of exopolysaccharide (EPS) and lipopolysaccharide (LPS) was demonstrated, such as *noeL*, *noeJ* and *rmlD* [7,28]; however, the presence of several unique genes suggests that EPS and LPS components might differ from one *Azospirillum* strain to another, a feature previously reported for LPS [7]. Only *A. brasilense* strains possess additional clusters of ancestral genes involved in EPS biosynthesis and/or transport (such as AZOBR_p310279, AZOBR_p330029, AZOBR_p60088, AZCBG_p60114, AZCBG_230032). Besides, *A. brasilense* genomes contain an additional gene involved in LPS biosynthesis and acquired by HGT (AZCBG_p190020; AZOBR_p210177). These genes might be relevant for the adaptation of azospirilla strains to their environment [29].

*Azospirillum* strains have gained different root-adhesion mechanisms. Indeed, TAD pili are exclusive to the *A. brasilense* species; these pili play an essential role in biofilm formation, colonization and pathogenesis in various genera [30] and their role in biofilm formation was recently assessed in *A. brasilense* Sp245 [12]. Cellulose synthesis is another mechanism by which bacteria can tightly bind to the roots [31]. Gene(s) involved in cellulose synthesis and acquired by HGT are found exclusively in *A. lipoferum* 4B and *Azospirillum* sp. B510. 

Cellulases and hemicellulases likely contribute to endophytic plant colonization, a property described for *A. brasilense* Sp245 and *Azospirillum* sp. B510 [20,32]. *Azospirillum* genomes were previously shown to encode a substantial number of glycosyl hydrolases (from 26 to 34), some of them likely capable of degrading plant cell wall [12]. However, some CAZy families are restricted to a species; genomes of *A. lipoferum* 4B and *Azospirillum* sp. B510 encode cellulases belonging to the GH8 and GH16 (licheninase) families whereas *A. brasilense* strains possess an endoglucanase of the GH12 family (AZOBR_p440082 and AZCBG_p490023). Both *A. brasilense* strains possess unique glycosyl hydrolase-encoding genes with no orthologues in any other *Azospirillum* genomes.

### 2.5. Strain-Specific Genes Involved in Plant Growth Promotion

The contribution of nitrogen fixation to plant growth promotion by *Azospirillum* is controversial; however, greenhouse and field inoculation experiments significantly reduced the required dozes of nitrogen fertilization [1]. In addition to the nitrogen fixation cluster that has been inherited vertically, *A. brasilense* Sp245 genome harbours a second cluster for nitrogen fixation (AZOBR_p350011 to AZOBR_p350024). This cluster encodes a vanadium nitrogenase and may have been horizontally transferred from *Rhodopseudomonas palustris*; an integrase lies just downstream of this operon (AZOBR_p350032). As the genome of *A. brasilense* CBG497 is not closed (part of the p3 is missing), the occurrence of this operon was searched by PCR: amplification with primers for *vnfX* (AZOBR_p350013), *vnfD* (AZOBR_p350020) and *vnfH* (AZOBR_p350022) was negative, suggesting that this operon has been acquired recently by *A. brasilense* Sp245.

Phosphate solubilization represents another important trait for enhancement of plant nutrition [33]. Interestingly, *A. lipoferum* 4B and *Azospirillum sp.* B510 may have acquired the ability to solubilize inorganic phosphates through the secretion of gluconic acid. Oxidation of glucose into gluconic acid takes place in the periplasm and is assumed by a PQQ-dependent glucose dehydrogenase (AZOLI_p50302 / AZL_e01560) [34]. No orthologue was found in the *A. brasilense* genomes; such a property which has been scarcely reported in the *Azospirillum* genus [35] deserves to be investigated.

Plant growth promotion by *Azospirillum* is thought to occur essentially through modulation of the plant hormonal balance via the synthesis of phytohormones, notably the phytohormone indole-3-acetic acid (IAA), or via the degradation of phytohormones or precursors of phytohormones, such as 1-aminocyclopropane-1-carboxylic acid (ACC) [36]. *A. brasilense* Sp245 is well-known to produce IAA from tryptophan (Trp) through the indole-3-pyruvate (IPyA) biosynthetic pathway [37,38,39]. One key enzyme of this pathway, encoded by *ppdC*/*ipdC*, is the indole-3-pyruvate decarboxylase, which mediates conversion of indole-3-pyruvate into indole-3-acetaldehyde (second step of the pathway). This gene is present in the genome of the two *A. brasilense* strains (AZOBR_40354 and AZCBG_190067), but absent from genomes of *A. lipoferum* 4B and *Azospirillum* sp. B510. The first and third steps of the IPyA pathway correspond respectively to the conversion of tryptophan into IPyA and of indole-3-acetaldehyde into IAA. These steps are catalyzed by enzymes, respectively aromatic aminotransferases and NAD-dependent aldehyde dehydrogenase, which are common and non-specific enzymes. Recently, the contribution of *hisC1*, which encodes an aromatic amino acid aminotransferase-1 (AAT1), to IAA production was evidenced in *A. brasilense* Sp7 [40]. Homologues sharing identity levels higher than 75% with AAT1 from Sp7 were found in all four *Azospirillum* genomes (AZOLI_1579, AZCBG_330158, AZOBR_120044, AZL_012940). 

In Sp245, it was shown that an *ipdC* knockout mutant still produced 10% of the wild-type IAA production level [39], indicating that other metabolic pathways contribute, even though to a lesser extent, to IAA production. Indeed, a large set of genetic and biochemical studies strongly suggest that *A. brasilense* might possess a triptamine (TAM) and an indole-3-acetonitrile (IAN) pathway [41,42]. The IAN pathway corresponds to the conversion of Trp into indole-3-acetaldoxime (IAox), of IAox into IAN and then of IAN into IAA. In *Arabidopsis thaliana,* two cytochrome P450 enzymes (CYP79B2 and CYT79B3) catalyze the formation of IAox from Trp, and two nitrilase genes *NIT1* and *NIT2* have been shown to contribute to IAA biosynthesis *in vivo* [41,43]*.* P-blast search performed on *Azospirillum* genomes with the two nitrilase protein sequences from *A. thaliana* revealed a putative nitrilase in *A. brasilense* Sp245 (AZOBR_p350044, respectively 48.9% and 49.84% identity with NIT1 and NIT2) and in *Azospirillum sp.* B510 (AZL_020600, respectively 36.09% and 34.78% identity); those genes were previously classified as HGT [12]. 

The indole-3-acetamide pathway (IAM) involves the decarboxylation of Trp into IAM by a Trp monooxygenase (*iaaM*), and the hydrolysis of IAM into IAA by an indole acetamide hydrolase (*iaaH*). The existence of this pathway was suggested in *Azospirillum sp.* B510, with candidates for *iaaM* and *iaaH* represented respectively by AZL_b03560 and AZL_b03580 [10], two genes that are unique to the B510 genome. However, AZL_b03560 appears rather encoding a triptamine oxidase that might be involved in the conversion of TAM into indole-3-acetaldehyde. In B510, it seems that IAM is produced rather from the conversion of IAN to IAM by a nitrile hydratase rather than directly from Trp by a Trp monooxygenase. Consistently, no homologues of *iaaM* from *Agrobacterium tumefaciens*, *Dickeya dadantii* or *Pseudomonas syringae* pv. *syringae* were found in any *Azospirillum* genomes. Interestingly, eight clustered genes unique to the B510 genome and previously classified as HGT encode putative nitrile hydratases (AZL_a09780, AZL_a09790, AZL_a09810, AZL_a09820, AZL_a09830, AZL_a09840, AZL_a09850, AZL_a09860) and are located near a transposase and a tRNA. Future studies are required to verify if all those genetic determinants are implicated in IAA biosynthesis in those strains, since HPLC analyses revealed the ability to produce IAA in the presence of Trp for *A. brasilense* Sp245 and CBG497 but this production was negligible for *A. lipoferum* 4B and *Azospirillum sp.* B510 (our unpublished results).

Besides the ability to produce IAA, it was investigated whether *Azospirillum* strains are able to catabolize this phytohormone. IAA catabolism has been characterized in some rhizobacteria such as *Pseudomonas putida*, and relies on the presence of the *iac* locus (for IAA catabolism) constituted of 10 genes with coding similarity to enzymes acting on indole or amidated aromatics and to proteins with regulatory or unknown function [44,45]. Homologs of the *iac* genes are present in *A. lipoferum* 4B (AZOLI_p10981 to AZOLI_p10991) and *Azospirillum sp.* B510 (AZL_a08890 to AZL_a08810). The IAA catabolism phenotype was investigated and a slight growth on 5 mM IAA as sole carbon and nitrogen source was observed in the two strains while no growth was observed in the two *A. brasilense* strains (data not shown). Thus, it appears that, unlike *A. brasilense* strains, *A. lipoferum* 4B and *Azospirillum sp.* B510 may not be able to produce IAA but can metabolize such a substrate.

The deamination of ACC is another key activity involved in the modulation of the plant hormonal balance by rhizobacteria. ACC is the immediate precursor of plant ethylene, and its deamination leads to a decrease of ethylene production in plants. Because ethylene inhibits root growth and may be produced in too large amounts during plant stress response, bacterial ACC deamination can enhance both root system development and plant stress tolerance [46]. The ACC deaminase activity is encoded by *acdS* that is widely distributed in Proteobacteria; among the *Azospirillum* genus, this gene is mostly harboured by strains of the *A. lipoferum* species and has been acquired by HGT [12,47,48]. Accordingly, *acdS* and *acdR* (encoding a *lrp*-like transcriptional activator of *acdS*) are absent from the *A. brasilense* Sp245 and CB497 genomes, whereas they lie on the second largest chromid in *A. lipoferum* 4B (*i.e.*, AZOLI_p20559 and AZOLI_p20560) and in *Azospirillum sp.* B510 (*i.e.*, AZL_b04170 and AZL_b04180). 

The plant hormonal balance might also be modulated by the degradation of salicylate into catechol via salicylate 1-monooxygenase (EC 1.14.13.1). Such an enzyme was identified in *A. lipoferum* 4B and in *A. brasilense* strains (AZOLI_p20435 / AZOBR_p480008 / AZCBG_p410058). AZOLI_p20435 displays only 36-37% identity with the *A. brasilense* orthologs and 81% to NahG of *Pseudomonas fluorescens* SBW25 whereas the *A. brasilense* orthologs display strong identity (67%–68%) with NahW of *Burkholderia xenovorans* LB400; this observation is consistent with two independent acquisitions through HGT [12]. Nevertheless only *A. lipoferum* 4B harbors the metabolic pathway for catechol degradation (see below) and thus may use salicylate as a source of energy and carbon.

### 2.6. Strain-Specific Catabolic Pathways Involved in Adaptation to the Rhizosphere

Examination of strain-specific genes revealed specific catabolic properties that might be relevant for adaptation to the rhizosphere. A complete ribose degradation pathway was identified in *A. lipoferum* 4B and *Azospirillum sp.* B510 involving a ribokinase (*rsbK*, AZOLI_p20179 / AZL_b03490), a deoxyribokinase/ribokinase (*deoK*, AZOLI_p20643 / AZL_b05870), and a deoxyribose mutarotase (*deoM*, AZOLI_p20642 / AZL_b05860). The catabolic pathway of myo-inositol was identified only in *Azospirillum sp.* B510 (AZL_b00950 and AZL_b01030 to AZL_b01060).

*A. lipoferum* 4B and *Azospirillum sp.* B510 may have the ability to degrade the organophosphonate 2-aminoethylphosphonate. Organophosphonates are quite abundant in nature, primarily as components of phosphonolipids, but also as constituents of polysaccharides, glycoproteins, glycolipids and several antibiotics. The enzymes catalyzing the first two steps, *i.e.*, 2-aminoethylphosphonate-pyruvate transaminase (*phnW*, AZOLI_p20203 / AZL_a10490) and phosphonoacetaldehyde hydrolase (*phnX*, AZOLI_p20204 / AZL_a10480) are present only in those two genomes.

*Azospirillum* sp. B510 may degrade and use the aliphatic amine methylamine as a nitrogen source. Various aliphatic amines can be emitted in agricultural systems, notably methylamine (MMA), dimethylamine and trimethylamine [49]. An alternative MMA oxidative pathway different from the direct oxidation carried out by MMA dehydrogenase was fully characterized on *Methyloversatilis universalis* FAM5 [50]. This eight gene cluster composed of *mgdABCD*, *gms* and *mgsABC* is present in other methylotrophs [51,52] and in nonmethylotrophs, including *Agrobacterium tumefaciens* C58 which can grow using MMA as sole nitrogen source. The genome of *Azospirillum* sp. B510 carries this cluster (AZL_a09510 to AZL_a09580), next to *purU* and *folD* encoding respectively formyltetrahydrofolate deformylase and methylenetetrahydrofolate dehydrogenase/cyclohydrolase that are necessary for the detoxification of formaldehyde generated by this metabolic pathway [53]. 

**Figure 6:**
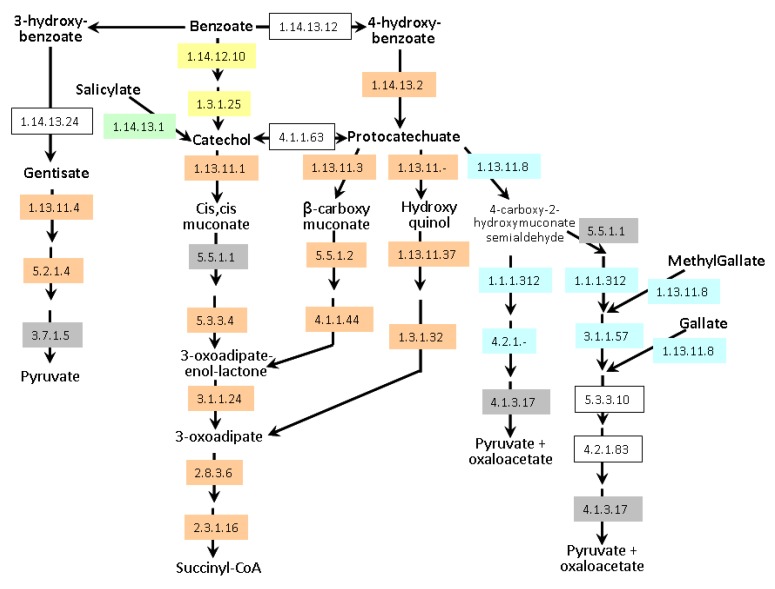
Aromatic compounds catabolism in *Azospirillum*. Enzymes framed in grey are encoded by all genomes. Enzymes framed in orange are encoded exclusively by genomes of *A. lipoferum* 4B and *Azospirillum* sp. B510. Enzymes framed in yellow are exclusive to *Azospirillum* sp. B510. Enzymes highlighted in blue are specific to the *A. brasilense* strains (Sp245 and CBG497). Conversion of salicylate into catechol (green) is encoded by all genomes except that of *Azospirillum* sp. B510. Non-colored enzymes are missing in all strains. Enzyme 1.1.1.312 was previously known as 1.2.1.45. Alternative names for gentisate and protocatechuate are respectively 2,5-hydroxybenzoate and 3,4-hydroxybenzoate.

Degradation of aromatic compounds (*i.e.,* organic molecules containing one or more aromatic rings mainly produced by plants) is dominated by aerobic and anaerobic bacteria and aerobic fungi [54]. The aerobic catabolism of aromatic compounds usually involves the oxygenolytic hydroxylation of the aromatic ring, producing central dihydroxylated aromatic intermediates (e.g., catechol, protocatechuate, gentisate, homoprotocatechuate, homogentisate and hydroxyhydroquinone). These intermediates are then cleaved by different types of ring-cleavage dioxygenases, generating aliphatic compounds that funnel into the tricarboxylic acid (TCA) cycle through a small number of central pathways [55]. Degradation of a broad spectrum of aromatic natural and xenobiotic compounds relies on two central intermediates: catechol and protocatechuate. *A. lipoferum* 4B and *Azospirillum sp.* B510 can degrade these compounds via the β-ketoadipate pathway (Figure 6); they also possess enzymes allowing the conversion of benzoate and 4-hydroxybenzoate into protocatechuate. In addition, *Azospirillum sp.* B510 can convert benzoate into catechol. Both strains can also metabolise gentisate (2,5-dihydroxybenzoate). *A. brasilense* strains seem to be less versatile as only the meta-cleavage pathway of protocatechuate degradation has been identified; catabolism of gallate and methylgallate might be present but one of the key enzymes (EC 4.2.1.83) could not be identified. Experimentally, growth on protocatechuate as the sole carbon source was observed for all strains except *A. brasilense* CBG497 (data not shown). As for transporters, *A. brasilense* strains possess a protocatechuate transporter with strong identity to a *Bradyrhizobium* transporter (AZOBR_p310195 in Sp245 and a gene present on the missing part of p3 in CBG497 as revealed by PCR). A gene encoding hydroxybenzoate transporter is found adjacent to the gentisate degradation pathway in *A. lipoferum* 4B and *Azospirillum sp.* B510 (AZOLI_p20645 and AZL_a09170) and displays identity with *pcaK* of *Ralstonia*. PcaK functions as a chemoreceptor for chemotaxis towards aromatic acids [56]. Additional hydroxybenzoate transporters are encoded by the genome of *Azospirillum sp.* B510 (AZL_a07380 and AZL_b03660).

The phenylacetate catabolic pathway is the central route where catabolic pathways of many aromatic compounds converge and are directed to the TCA cycle [57]. The aerobic degradation of phenylacetate by epoxidation of CoA thioesters occurs in ~16% of all bacterial species with sequenced genomes [54]. Within the genomes of *Azospirillum*, only *A. lipoferum* 4B and *Azospirillum sp.* B510 strain harbour the complete *paa* catabolic cluster; 11 of these loci are located on the p4/d replicon (AZOLI_p40257 to AZOLI_p40267), whereas *paaX, paaY,* and *paaF* (which is duplicated) are located onto the chromosome, thus constituting five different clusters, an organization previously reported in *Pseudomonas putida* [58]*.* Seven of the genes located on p4 (*paaA, paaB, paaC, paaD, paaE, paaN* and *paaJ*) have been classified as horizontally transferred whereas the others have been classified as ancestral [12], suggesting a complex evolution of this catabolic pathway. The functionality of the *paa* genes was assessed as both strains were able to grow on phenylacetic acid as the sole carbon source, whereas the *A. brasilense* strains showed no growth (Supplementary Figure S2). 

Thus, *A. lipoferum* 4B and *Azospirillum sp.* B510 seem to be more versatile for aromatic compound degradation than *A. brasilense* strains; indeed, in addition to the above-mentioned pathways, several aromatic ring-hydroxylating dioxygenases could be identified. It will be interesting to determine if this versatility is related to the composition of the host plant exudates, as a result of niche-specific adaptation. This versatility could also be related to environmental conditions, such as soil type and cultural practices (like flooding for rice) that can greatly influence the microbial community.

### 2.7. Other Strain-Specific Genes Likely Involved in Adaptation to the Rhizosphere

*Azospirillum* strains are not equally equipped for iron acquisition, an important component of bacterial metabolism. Discrepancies are observed at the level of siderophores biosynthesis and uptake. A 14 kb region that spans AZOLI_p20158 to AZOLI_p20165 is predicted to be involved in pyochelin biosynthesis and appeared to be exclusively present in the *A. lipoferum* 4B genome. The corresponding genes, classified as HGT [12], show protein identity levels greater than 45% with those of *Pseudomonas fluorescens* and a high degree of synteny. A cluster of genes involved in enterobactin biosynthesis was specifically found in the two *A. brasilense* strains (AZOBR_p350073 to AZOBR_p350079 / ACBG_p22001 to AZCBG_p22007). *A. brasilense* Sp245 possesses a unique cluster of eight genes predicted to be involved in siderophore transport (AZOBR_220054 to AZOBR_p220061); a gene encoding a *fecI*–type sigma factor is located directly upstream of this cluster (AZOBR_p220053) and is similar to PsbS/PvdS, a sigma-70 ECF of *Pseudomonas* (56% of identity at the protein level with PsbS of *Pseudomonas* sp. B10). PvdS was shown to regulate the transcription of pyoverdine biosynthesis genes under iron starvation in *P. aeruginosa* [59]. A hemin ABC transporter is also present in the genomes of *A. brasilense* Sp245 and *A. lipoferum* 4B. As plant colonization ability has been shown to be linked to iron acquisition systems [60], discrepancies observed among azospirilla could be relevant.

Plants are capable of producing reactive oxygen species (ROS), as a defence mechanism against both pathogenic and symbiotic bacteria [61,62]. Living organisms have built up mechanisms to protect themselves against oxidative stress, with antioxidant enzymes such as catalase and superoxide dismutase, small proteins like thioredoxin and glutaredoxin, and molecules such as glutathione. Comparative genomic analysis shows that next to common mechanisms, several enzymes involved in the oxidative stress response differ among *Azospirillum* species. For example, a gene encoding a superoxide dismutase (SodA) is found uniquely in *A. brasilense* strains (AZOBR_p440007 / AZCBG_p410047), whereas a gene encoding a catalase is present in *A. lipoferum* 4B and in *Azospirillum sp.* B510 (AZOLI_p10486 / AZL_a00280). Moreover, a bifunctional catalase-peroxidase (KatG) is only found in *A. lipoferum* 4B (AZOLI_p30178). The deduced amino acid sequence of this ORF have 80% identity with the KatG protein of *Rhizobium etli*, and is directly located downstream of the gene encoding the OxyR transcription factor. KatG plays a role in survival during stationary-phase in *R. etli*, but is not essential for nodulation and nitrogen fixation in symbiosis with *Phaseolus vulgaris* [63]. 

Components of a type VI secretion system (T6SS) have previously been identified in the three published genomes and classified as HGT [12]; however the T6SS components display discrepancies among strains. A region with an organization similar to that of Alpha-proteobacteria (such as *Azorhizobium*) is located on the chromosome of *A. lipoferum* 4B (AZOLI_0998 to AZOLI_1020) and *Azospirillum* sp. B510 (AZL_017990 to AZL_017770). A second region exclusive to *A. lipoferum* 4B (AZOLI_p30482 to AZOLI_p30489) displays similarities with T6SS of *Bradyrhizobium japonicum* USDA110. A third region unrelated to the previous ones lies on the biggest chromid of the two *A. brasilense* strains and of *Azospirillum* sp. B510, and may have been acquired from Beta-proteobacteria. 

T6SS are involved in a broad variety of bacterial functions: from pathogenesis (by delivering effectors to target eukaryotic cells) to biofilm formation and stress sensing [64]. This large set of functions is reflected by a vast diversity of regulatory mechanisms [65]. T6SS can also confer toxicity towards other bacteria, providing a means of interspecies competition to enhance environmental survival [66]. Upregulation of *A. brasilense* Sp245 T6SS in response to exposure to IAA, as could happen in the rhizosphere, favors a role in plant-bacteria interactions [67]; the role of T6SS thus deserves to be investigated.

Several ORFs in all four *Azospirillum* genomes have been annotated as laccase-like; however, only one ORF (AZOLI_p30139 in *A. lipoferum* 4B and AZL_c02540 in *Azospirillum* sp. B510) possesses two typical copper-binding motifs [68]. Laccases- or laccase-like multicopper oxidases (EC 1.10.3.2) catalyze the oxidation of various substrates, such as phenols, diamines and metals, coupled with the reduction of molecular oxygen to water. The first report of a prokaryotic laccase is from *A. lipoferum* 4B [69], where it was shown to play a role in melanization and utilization of plant phenolic compounds [70]. Moreover, laccase-positive strains are less sensitive to the inhibitory action of quinone analogs due to rearrangements of their respiratory chain, a feature that might be a competitive advantage in the rhizosphere in the presence of quinone compounds [71]. A survey of bacterial laccases suggests they are an advantageous trait for a rhizosphere bacterium as they are involved in various functions such as copper resistance, manganese oxidation, pigmentation, oxidation of toxic compounds, and destruction of reactive oxygen species [72]. 

### 2.8. Accessory Components Related to Genome Plasticity

The extraordinary genome plasticity of *Azospirillum* has been evidenced by experimental data [14,73] and by whole genome alignments [12]. However, comparison of the AZO-core and the ANC-core does not allow the identification of key determinants that could partly explain this genomic plasticity, such as genes encoding recombinases, resolvases or topoisomerases. 

Genomic regions carrying prophage elements seem to be specific to each strain; the only related element present in the AZO-core encodes a phage-related lysozyme (AZOLI_2690 / AZL_003440 / AZOBR_20012 / AZCBG_120001). This gene is absent from the ANC-core despite its initial assignment as ancestral [12]; this discrepancy comes from the fact that an orthologue is present in the genome of *Magnetospirillum magneticum* but absent from the genome of *R. centenum* used here to establish the ANC-core. The genomic context of this gene is identical in the four *Azospirillum* strains but does not display any other phage-related genes, suggesting that this gene is a phage remnant.

In *A. brasilense* Sp245, a unique region encompassing about 65 kb (from AZOBR_p340083 to AZOBR_p340194) contains several ORFs of phage origin and many ORFs encoding proteins of unknown function, and is framed by transposase/integrase. This region could correspond to the 65-kb prophage previously isolated from this strain [15]. This putative prophage of *A. brasilense* Sp245 shows no homology to a prophage sequence obtained from *A. brasilense* Cd, an observation which is consistent with the absence of hydridization signal previously reported [15]; in addition, this region has no equivalent in the genome of *A. brasilense* CBG497. Blast search in the genome of *A. brasilense* CBG497 using the sequence of *A. brasilense* Cd prophage reveals a single hit with the above mentioned ORF encoding a phage-related lysozyme (AZCBG_120001); no other ORF of phage origin was found in the genome of *A. brasilense* CBG497. The release of phage particles upon induction by mitomycin C was previously reported for several strains of *Azospirillum* [15]. When the same procedure was applied to *A. brasilense* CBG497, no lysis was observed indicating that CBG497 hosts no mitomycin C-inducible prophage (data not shown). Whereas all phages from *A. brasilense* strains displayed genomes sizes from 62 to 65 kb, phages from *A. lipoferum* strains (including 4B) and from *Azospirillum* sp. B510 displayed a size of about 10 kb. Several pieces of evidence imply that these small prophages are rather gene transfer agents (GTA) than real prophages [15]. GTAs typically package bacterial genome fragments and atypically package a portion of their own genome and constitute conspicuous mechanisms of generalized transduction; they seem to be widespread among Alpha-proteobacteria [74]. However identification of GTA genes from sequenced genomes is not always straighforward as GTA genes can be scattered throughout the genome [74,75]. 

Two chromosomal regions with their putative *att* sites were previously identified as prophages in the genome of *Azospirillum* sp. B510 [10]: B510PP01, a region of 66.7 kb that is partially duplicated (60.2 kb) and B510PP02, a region of approximately 20 kb. Only the two ORFs framing B510PP01 (*i.e.*, AZL_008150 and AZL_008670) have orthologues in the *A. lipoferum* 4B genome (respectively, AZOLI_2072 and AZOLI_2071), suggesting that this phage may no longer be present in the latter strain. Blast searches with GTA genes of *Rhodobacter capsulatus* (accession number AF181080) identify putative GTA genes in the two duplicated sequences of B510PP01 (identity >25%); moreover, a stretch of four contiguous genes (encoding terminase / portal protein / prohead protease / capsid) display a similar organization than the corresponding GTA genes of *R. capsulatus*. Thus it is likely that B510PP01 or part of B510PP01 corresponds to a GTA.

Four prophage regions are present in *A. lipoferum* 4B. First, a chromosomal region of 31.4 kb (AZOLI_1757 to AZOLI_1794) harbours four genes (AZOLI_1775 to AZOLI_1771) that despite no homology at the DNA level display a similar organization with GTA genes of *R. capsulatus* encoding terminase, phage portal protein, phage prohead protease and capsid. This region could correspond to the phage particles containing random 10-kb fragments of host genomic DNA [15]. Three other regions bear chromids (AZOLI_p10448 to AZOLI_p10472, 25.5 kb; AZOLI_p10780 to AZOLI_p10794, 13.6 kb; AZOLI_p20026 to AZOLI_p20039, 13.1 kb) and have similarities with lambda-type or Mu-type prophages.The tRNAs lie upstream or downstream of these three regions. 

So *A. lipoferum* 4B and *Azospirillum* sp. B510 have been subjected to multiple phage infection events, that may have contributed to genomic rearrangements. Moreover, GTA mediating generalized transduction may have contributed to acquisition of foreign DNA.

Other elements that certainly contribute to the high genomic plasticity of *Azospirillum* genomes are CRISPR sequences (*i.e.*, Clustered Regularly Interspaced Short Palindromic Repeats); CRISPR are thought to be involved in repartition of genome copies during cell division, to facilitate recombination and act as a defence mechanism against phages [76]. CRISPR were previously identified in 4B (126), B510 (153) and Sp245 (12) [12] and searched in CBG497 using the CRISPR web interface [77]. Seventeen CRISPR could thus be identified in the genome of *A. brasilense* CBG497 (on chromosome and on p2). Such smaller numbers of CRISPR in the genomes of *A. brasilense* strains might be due to their unclosed status or to their limited exposure to phage infections. Finally, a detailed analysis of the two closed genomes (those of *A. lipoferum* 4B and *Azospirillum* sp. B510) revealed the presence of multiple insertion sequences scattered in all the replicons [12]: 99 IS belonging to 37 different families in *A. lipoferum* 4B and 310 IS belonging to 59 different families in *Azospirillum* sp. B510. Altogether, these features may have contributed to shape *Azospirillum* genome and to promote rearrangements between the different replicons. 

## 3. Experimental Section

### 3.1. DNA Sequencing

DNA extraction of *A. brasilense* CBG497 and sequencing using the pyrosequencing method was performed as previously described [18]. 156 contigs were assembled into six replicons according to the genome organization of *A. brasilense* Sp245 using the MUMer software [78]. The sequence and annotations are available from the MicroScope platform [79,80].

### 3.2. Phylogenetic Analysis and Genome Comparisons

The 16S rRNA sequences were downloaded from EMBL. The sequences were first aligned using MUSCLE [81] and a maximum likelihood tree was generated using the SeaView platform [82]. To cluster protein families, BLAST-P comparisons of ‘‘all *versus* all’’ complete proteomes of *A. brasilense* CBG457, *A. brasilense* Sp245, *A. lipoferum* 4B and *Azospirillum* sp. B510 were done. Clustering was achieved with MCL using an e-value of 10^-7^ and an inflation parameter of 1.2 [22]. 

### 3.3. Orthologues Grouping and Analysis of Evolutionary Rates

The most probable set of orthologous proteins shared by the four *Azospirillum* strains (designated AZO core) was identified using a reciprocal best-hit criterion. To that end, all the predicted proteins of one genome were searched against the other predicted proteomes and vice versa using BLAST with cutoff e-value of 10^−^^12^ and employing the Blosum-80 matrix [83]. In addition to this criterion, to be included in an orthologue group, the alignment region between the subject protein and the query protein had to be at least 75%, and there had to be at least 35% similarity of both query and target sizes. 2,328 orthologue groups were identified in *Azospirillum*. Exclusive genes were recorded as those with no hit in the genomes at e-value of <10^−^^6^. COG categories for each orthologue group were retrieved from the MaGe platform on the genome of *A. lipoferum* 4B; when several COGs were attributed, the COG with the highest score was retained. The same procedure was performed with an additional genome, that of *Rhodospirillum centenum* SW, to determine the ancestral core genome (designated ANC core). The number of nucleotide substitutions per synonymous site ‘‘Ks’’ and the number of nucleotide substitutions per non-synonymous site ‘‘Ka’’ were determined with the KaKs Calculator v1.2 software testing all different selection models and model averaging [84].

### 3.4. Bench Experiments

PCR amplifications were performed according to the *Taq* polymerase manufacturer (Invitrogen, Cergy-Pontoise, France) in 25 μL using 50 ng of template DNA. The amplification cycle consisted of an initial 5 min at 95 °C; 35 cycles of 30 s at 95 °C, 30 s at annealing temperature, and 30 s at 72 °C; followed by a final 7-min extension at 72 °C. Genomic DNA was extracted from bacterial cultures grown for 20 h in LBm broth with a DNeasy Tissue Kit (Qiagen, Courtaboeuf, France) according to the manufacturer’s instructions. The following PCR primers (synthesized by Invitrogen) have been used: F9504 (GATCAGGCCCAAGTCAACCT) and F9505 (GTTCAGCAGGTCCAGGATGT) for *vnfX* (AZOBR_p350013); F9510 (GCATGATCGTCTACACCACCT) and F9511 (GATGCCGTACTGCTTCTTCAG) for *vnfD* (AZOBR_p350020); F9536 (GTCAAGACCATGTCGAAGACC) and F9537 (GGAGTTCTTCAGCTCCAGGAT) for *vnfH* (AZOBR_p350022); F9528 (ATGGGGCTGGTGATCTTCTAC) and F9529 (GAAGATGCTGGTGAAGTCGAG) for AZOBR_p310195 (coding for a protocatechuate transporter).

For degradatation of aromatic compounds, precultures of *Azospirillum* were performed in AB malate 0.2% over 16 to 20 h at 28 °C under agitation. Cells were pelleted, washed and inoculated at an optical density of 0.05 into AB medium containing 0.1% phenylacetate or protocatechuate as sole carbon source. Stock solutions of aromatic compounds were prepared at 20% (w/v) in dimethyl sulphoxide. Growth was performed at 28 °C under agitation and monitored at 580 nm for the experiment with phenylacetate.

For utilization of IAA, cells were precultured and washed as above and were inoculated into AB medium containing 5 mM IAA as sole carbon source (stock solution of IAA at 0.5 M was prepared in acetonitrile). Growth was performed at 28 °C under agitation and recorded after three days.

Induction of phage particles by mitomycin C treatment was performed on *A. brasilense* CBG497 as previously described [15].

## 4. Conclusions

The genome of *A. brasilense* CBG497, a strain isolated from maize grown on an alkaline soil in the northeast of Mexico, was obtained and comparative analyses were performed with three *Azospirillum* genomes previously described. The four *Azospirillum* genomes studied here have genomes ranging from 6.5 to 7.6 Mbp, and are composed of six or seven replicons; chromids and plasmids comprise the largest proportion of the total genome (from 55.2% to 59.8%). The *Azospirillum* core genome consists of 2,328 proteins, representing between 30% to 38% of the total encoded proteins within a genome. It is mainly located on chromosome and contains 74% of genes of ancestral origin shared with some aquatic relatives. The non-ancestral part of the core genome is enriched in gene involved in signal transduction, in transport and metabolism of carbohydrates and amino-acids, and in surface properties, features linked to adaptation to soil and rhizosphere. However, many strain-specific or species-specific genes exhibit functions related to colonization of plant roots (chemotaxis, synthesis of surface polysaccharides, TAD pili), to plant-growth promotion (notably biosynthesis of hormones) and more generally to rhizosphere competence (catabolism of aromatic compounds, iron uptake). Thus, it appears that although *Azospirillum* strains harbour a common set of genes relevant for adaptation to the rhizosphere, each species or strain possesses unique genetic determinants, evidencing niche-specific adaptation. In addition, all genomes contain accessory components related to genome plasticity that could promote acquisition of foreign DNA or rearrangements between replicons. Transcriptomics approaches on *Azospirillum* during their interaction with host plants are now being developed in order to identify bacterial genetic determinants that are essential for this associative symbiosis.

## Supplementary Files

Supplementary File 1 Supplemental Table (PDF, 271 KB)

Supplementary File 2 Supplementary Figures (PDF, 156 KB)
