# Supplementary material for: Genome Sequence of Azospirillum brasilense CBG497 and Comparative Analyses of Azospirillum Core and Accessory Genomes provide Insight into Niche Adaptation
Source: Genes (Basel). 2012 Sep 28;3(4):576–602. doi: 10.3390/genes3040576 (PMC3899980; doi:10.3390/genes3040576)
Supplement: Supplementary File 1 — Supplemental Table (PDF, 271 KB) [file genes-03-00576-s001.pdf]

Supplementary Table 1: List of the orthologous proteins shared by the four *Azospirillum* strains (all) and by *Rhodospirillum centenum* SW (highlighted in grey)

***A. lipoferum* 4B**	***Azospirillum* Sp. B510**	***A. brasilense* Sp245**	***A. brasilense* CBG497**
AZOLI_0004	AZL_026610	AZOBR_10111	AZCBG_40001
AZOLI_0005	AZL_026620	AZOBR_10112	AZCBG_40002
AZOLI_0007	AZL_026640	AZOBR_10212	AZCBG_40093
AZOLI_0008	AZL_026650	AZOBR_10211	AZCBG_40092
AZOLI_0010	AZL_026670	AZOBR_10275	AZCBG_60025
AZOLI_0011	AZL_026680	AZOBR_10276	AZCBG_70001
AZOLI_0016	AZL_026570	AZOBR_30027	AZCBG_140012
AZOLI_0024	AZL_026540	AZOBR_10035	AZCBG_20014
AZOLI_0027	AZL_026530	AZOBR_p170008	AZCBG_p1100012
AZOLI_0028	AZL_026520	AZOBR_p1110084	AZCBG_p130079
AZOLI_0031	AZL_026510	AZOBR_20022	AZCBG_130003
AZOLI_0034	AZL_026490	AZOBR_200030	AZCBG_490208
AZOLI_0036	AZL_026470	AZOBR_200028	AZCBG_490206
AZOLI_0037	AZL_026460	AZOBR_200027	AZCBG_490205
AZOLI_0038	AZL_026450	AZOBR_200026	AZCBG_490204
AZOLI_0048	AZL_026400	AZOBR_10444	AZCBG_100012
AZOLI_0049	AZL_026390	AZOBR_10442	AZCBG_100010
AZOLI_0050	AZL_026380	AZOBR_10441	AZCBG_100009
AZOLI_0053	AZL_026360	AZOBR_10425	AZCBG_80054
AZOLI_0054	AZL_026350	AZOBR_10423	AZCBG_80053
AZOLI_0055	AZL_026340	AZOBR_10420	AZCBG_80050
AZOLI_0057	AZL_026320	AZOBR_10418	AZCBG_80049
AZOLI_0059	AZL_026300	AZOBR_10416	AZCBG_80047
AZOLI_0060	AZL_026290	AZOBR_10415	AZCBG_80046
AZOLI_0064	AZL_026260	AZOBR_10410	AZCBG_80043
AZOLI_0066	AZL_026250	AZOBR_10409	AZCBG_80042
AZOLI_0067	AZL_026240	AZOBR_10408	AZCBG_80041
AZOLI_0069	AZL_026220	AZOBR_10406	AZCBG_80039
AZOLI_0070	AZL_026210	AZOBR_10302	AZCBG_70027
AZOLI_0074	AZL_026200	AZOBR_10301	AZCBG_70026
AZOLI_0075	AZL_026190	AZOBR_10300	AZCBG_70025
AZOLI_0077	AZL_026180	AZOBR_10298	AZCBG_70024
AZOLI_0078	AZL_026170	AZOBR_10297	AZCBG_70023
AZOLI_0082	AZL_026140	AZOBR_30005	AZCBG_150006
AZOLI_0087	AZL_026090	AZOBR_100039	AZCBG_270057
AZOLI_0092	AZL_026040	AZOBR_40255	AZCBG_160196
AZOLI_0095	AZL_026030	AZOBR_10286	AZCBG_70011
AZOLI_0096	AZL_026020	AZOBR_10285	AZCBG_70010
AZOLI_0106	AZL_026010	AZOBR_10193	AZCBG_40077
AZOLI_0107	AZL_026000	AZOBR_10192	AZCBG_40076
AZOLI_0108	AZL_025990	AZOBR_10191	AZCBG_40075
AZOLI_0112	AZL_025960	AZOBR_10185	AZCBG_40071
AZOLI_0118	AZL_025910	AZOBR_10180	AZCBG_40066
AZOLI_0121	AZL_003040	AZOBR_10179	AZCBG_40064
AZOLI_0122	AZL_003050	AZOBR_10178	AZCBG_40063
AZOLI_0124	AZL_003060	AZOBR_10176	AZCBG_40061
AZOLI_0125	AZL_003070	AZOBR_40106	AZCBG_160065
AZOLI_0126	AZL_0344	AZOBR_40108	AZCBG_160067
AZOLI_0130	AZL_003090	AZOBR_140223	AZCBG_370024
AZOLI_0131	AZL_003100	AZOBR_140224	AZCBG_370025
AZOLI_0132	AZL_003110	AZOBR_140225	AZCBG_370026
AZOLI_0133	AZL_003120	AZOBR_140226	AZCBG_370027
AZOLI_0134	AZL_002550	AZOBR_180257	AZCBG_490143
AZOLI_0135	AZL_002560	AZOBR_180256	AZCBG_490142
AZOLI_0136	AZL_002570	AZOBR_180255	AZCBG_490141

AZOLI_0139	AZL_002600	AZOBR_180253	AZCBG_490139
AZOLI_0140	AZL_002610	AZOBR_180251	AZCBG_490138
AZOLI_0141	AZL_002620	AZOBR_180250	AZCBG_490137
AZOLI_0142	AZL_002630	AZOBR_180247	AZCBG_490136
AZOLI_0144	AZL_002660	AZOBR_p470067	AZCBG_p4230017
AZOLI_0145	AZL_002670	AZOBR_p470068	AZCBG_p4230018
AZOLI_0146	AZL_002680	AZOBR_p470069	AZCBG_p4230019
AZOLI_0147	AZL_002690	AZOBR_p470070	AZCBG_p4230020
AZOLI_0149	AZL_002710	AZOBR_180244	AZCBG_490134
AZOLI_0153	AZL_002750	AZOBR_p460095	AZCBG_p4180033
AZOLI_0154	AZL_002760	AZOBR_180243	AZCBG_490133
AZOLI_0157	AZL_002790	AZOBR_180235	AZCBG_490126
AZOLI_0159	AZL_002810	AZOBR_180232	AZCBG_490124
AZOLI_0160	AZL_002820	AZOBR_180230	AZCBG_490122
AZOLI_0163	AZL_002840	AZOBR_180227	AZCBG_490119
AZOLI_0164	AZL_002850	AZOBR_70092	AZCBG_230101
AZOLI_0165	AZL_002860	AZOBR_70093	AZCBG_230102
AZOLI_0166	AZL_002870	AZOBR_70094	AZCBG_230104
AZOLI_0172	AZL_002910	AZOBR_40090	AZCBG_160050
AZOLI_0173	AZL_002920	AZOBR_40091	AZCBG_160051
AZOLI_0174	AZL_002930	AZOBR_40092	AZCBG_160052
AZOLI_0178	AZL_002950	AZOBR_70028	AZCBG_480059
AZOLI_0181	AZL_002970	AZOBR_p440141	AZCBG_p4110006
AZOLI_0194	AZL_2936	AZOBR_180172	AZCBG_490077
AZOLI_0199	AZL_025860	AZOBR_180166	AZCBG_490072
AZOLI_0201	AZL_025840	AZOBR_p470024	AZCBG_p4200007
AZOLI_0204	AZL_025780	AZOBR_200166	AZCBG_520001
AZOLI_0207	AZL_025750	AZOBR_200162	AZCBG_510069
AZOLI_0209	AZL_025730	AZOBR_200160	AZCBG_510067
AZOLI_0212	AZL_025690	AZOBR_200158	AZCBG_510064
AZOLI_0213	AZL_025680	AZOBR_200157	AZCBG_510063
AZOLI_0215	AZL_025660	AZOBR_p1120043	AZCBG_p140059
AZOLI_0217	AZL_025640	AZOBR_40041	AZCBG_160003
AZOLI_0218	AZL_025630	AZOBR_40040	AZCBG_160002
AZOLI_0220	AZL_025610	AZOBR_40038	AZCBG_140001
AZOLI_0221	AZL_025590	AZOBR_40036	AZCBG_140003
AZOLI_0222	AZL_025580	AZOBR_40034	AZCBG_140004
AZOLI_0227	AZL_025320	AZOBR_40394	AZCBG_210009
AZOLI_0229	AZL_025310	AZOBR_40395	AZCBG_210010
AZOLI_0230	AZL_025300	AZOBR_40396	AZCBG_210011
AZOLI_0231	AZL_025290	AZOBR_40397	AZCBG_210012
AZOLI_0236	AZL_025180	AZOBR_180199	AZCBG_490094
AZOLI_0237	AZL_003910	AZOBR_40127	AZCBG_160083
AZOLI_0238	AZL_003920	AZOBR_20025	AZCBG_130006
AZOLI_0241	AZL_003930	AZOBR_20027	AZCBG_130008
AZOLI_0242	AZL_003940	AZOBR_20028	AZCBG_130009
AZOLI_0243	AZL_003950	AZOBR_20029	AZCBG_130010
AZOLI_0247	AZL_003980	AZOBR_40145	AZCBG_160099
AZOLI_0248	AZL_003990	AZOBR_40148	AZCBG_160102
AZOLI_0252	AZL_004010	AZOBR_10053	AZCBG_20040
AZOLI_0254	AZL_004030	AZOBR_10055	AZCBG_20038
AZOLI_0255	AZL_004040	AZOBR_10056	AZCBG_20037
AZOLI_0256	AZL_004050	AZOBR_10058	AZCBG_20035
AZOLI_0257	AZL_004060	AZOBR_10060	AZCBG_20033
AZOLI_0266	AZL_004130	AZOBR_140294	AZCBG_370091
AZOLI_0269	AZL_004150	AZOBR_p1110083	AZCBG_p130078
AZOLI_0272	AZL_004160	AZOBR_p1110082	AZCBG_p130077
AZOLI_0274	AZL_004170	AZOBR_p1110081	AZCBG_p130076
AZOLI_0275	AZL_004180	AZOBR_p1110080	AZCBG_p130075

AZOLI_0276	AZL_004190	AZOBR_p1110079	AZCBG_p130074
AZOLI_0277	AZL_004200	AZOBR_p1110078	AZCBG_p130073
AZOLI_0279	AZL_004210	AZOBR_p1110076	AZCBG_p130071
AZOLI_0281	AZL_004230	AZOBR_p1110074	AZCBG_p130069
AZOLI_0282	AZL_004240	AZOBR_40257	AZCBG_160201
AZOLI_0284	AZL_004260	AZOBR_40261	AZCBG_170001
AZOLI_0285	AZL_004270	AZOBR_40262	AZCBG_170002
AZOLI_0286	AZL_004280	AZOBR_40263	AZCBG_170004
AZOLI_0287	AZL_004290	AZOBR_40267	AZCBG_170007
AZOLI_0288	AZL_004300	AZOBR_40268	AZCBG_170008
AZOLI_0290	AZL_004310	AZOBR_40269	AZCBG_170009
AZOLI_0294	AZL_004330	AZOBR_40270	AZCBG_180001
AZOLI_0295	AZL_004340	AZOBR_40271	AZCBG_180002
AZOLI_0296	AZL_004350	AZOBR_40272	AZCBG_180003
AZOLI_0297	AZL_004360	AZOBR_180192	AZCBG_490088
AZOLI_0299	AZL_004380	AZOBR_180184	AZCBG_490086
AZOLI_0301	AZL_004390	AZOBR_180183	AZCBG_490085
AZOLI_0303	AZL_004400	AZOBR_180182	AZCBG_490084
AZOLI_0306	AZL_004430	AZOBR_p440142	AZCBG_p4110007
AZOLI_0308	AZL_004440	AZOBR_p440145	AZCBG_p4110010
AZOLI_0309	AZL_004450	AZOBR_p440051	AZCBG_p480035
AZOLI_0313	AZL_004480	AZOBR_p440110	AZCBG_p490045
AZOLI_0314	AZL_004500	AZOBR_p440108	AZCBG_p490043
AZOLI_0315	AZL_004510	AZOBR_p440107	AZCBG_p490042
AZOLI_0317	AZL_004550	AZOBR_p440102	AZCBG_p490038
AZOLI_0318	AZL_004540	AZOBR_p440103	AZCBG_p490039
AZOLI_0319	AZL_004530	AZOBR_p440104	AZCBG_p490040
AZOLI_0320	AZL_004520	AZOBR_p440105	AZCBG_p490041
AZOLI_0324	AZL_0507	AZOBR_p440096	AZCBG_p490033
AZOLI_0326	AZL_004580	AZOBR_p440095	AZCBG_p490032
AZOLI_0327	AZL_004590	AZOBR_p440094	AZCBG_p490031
AZOLI_0328	AZL_004600	AZOBR_p440093	AZCBG_p490030
AZOLI_0329	AZL_004610	AZOBR_p1150022	AZCBG_p150281
AZOLI_0348	AZL_004960	AZOBR_70161	AZCBG_240023
AZOLI_0349	AZL_004970	AZOBR_70163	AZCBG_240024
AZOLI_0350	AZL_004980	AZOBR_70164	AZCBG_240025
AZOLI_0351	AZL_004990	AZOBR_70165	AZCBG_240026
AZOLI_0352	AZL_005000	AZOBR_70166	AZCBG_250001
AZOLI_0353	AZL_005010	AZOBR_90016	AZCBG_270015
AZOLI_0354	AZL_005020	AZOBR_90017	AZCBG_270016
AZOLI_0355	AZL_005030	AZOBR_90018	AZCBG_270017
AZOLI_0357	AZL_005040	AZOBR_70169	AZCBG_250004
AZOLI_0358	AZL_005050	AZOBR_70170	AZCBG_250005
AZOLI_0359	AZL_005060	AZOBR_40175	AZCBG_160123
AZOLI_0360	AZL_005070	AZOBR_40177	AZCBG_160124
AZOLI_0361	AZL_005080	AZOBR_40178	AZCBG_160125
AZOLI_0362	AZL_005090	AZOBR_40179	AZCBG_160126
AZOLI_0363	AZL_005100	AZOBR_40180	AZCBG_160127
AZOLI_0365	AZL_005110	AZOBR_40181	AZCBG_160128
AZOLI_0366	AZL_005120	AZOBR_40182	AZCBG_160129
AZOLI_0367	AZL_005130	AZOBR_40183	AZCBG_160130
AZOLI_0369	AZL_005140	AZOBR_40185	AZCBG_160132
AZOLI_0370	AZL_005150	AZOBR_40187	AZCBG_160133
AZOLI_0371	AZL_005160	AZOBR_40188	AZCBG_160134
AZOLI_0372	AZL_005170	AZOBR_40189	AZCBG_160135
AZOLI_0373	AZL_005180	AZOBR_40190	AZCBG_160136
AZOLI_0374	AZL_005190	AZOBR_40191	AZCBG_160137
AZOLI_0376	AZL_005210	AZOBR_40142	AZCBG_160096
AZOLI_0378	AZL_005230	AZOBR_40140	AZCBG_160094

AZOLI_0379	AZL_005240	AZOBR_40139	AZCBG_160093
AZOLI_0380	AZL_005250	AZOBR_40138	AZCBG_160092
AZOLI_0381	AZL_005260	AZOBR_40136	AZCBG_160090
AZOLI_0383	AZL_005280	AZOBR_40133	AZCBG_160088
AZOLI_0384	AZL_005290	AZOBR_110003	AZCBG_330005
AZOLI_0385	AZL_005300	AZOBR_170007	AZCBG_480022
AZOLI_0388	AZL_005330	AZOBR_170004	AZCBG_480019
AZOLI_0389	AZL_005340	AZOBR_170003	AZCBG_480018
AZOLI_0392	AZL_005380	AZOBR_170001	AZCBG_480016
AZOLI_0398	AZL_005430	AZOBR_160049	AZCBG_450018
AZOLI_0402	AZL_005460	AZOBR_160070	AZCBG_480007
AZOLI_0403	AZL_005470	AZOBR_160071	AZCBG_480008
AZOLI_0410	AZL_005520	AZOBR_160076	AZCBG_480012
AZOLI_0411	AZL_005530	AZOBR_160077	AZCBG_480013
AZOLI_0412	AZL_005540	AZOBR_160078	AZCBG_480014
AZOLI_0413	AZL_005560	AZOBR_160043	AZCBG_450012
AZOLI_0414	AZL_005570	AZOBR_160042	AZCBG_450011
AZOLI_0415	AZL_005580	AZOBR_160041	AZCBG_450010
AZOLI_0416	AZL_005590	AZOBR_160039	AZCBG_450009
AZOLI_0421	AZL_005610	AZOBR_160036	AZCBG_450006
AZOLI_0422	AZL_005620	AZOBR_160033	AZCBG_450004
AZOLI_0436	AZL_005640	AZOBR_160017	AZCBG_450001
AZOLI_0442	AZL_005660	AZOBR_160014	AZCBG_440012
AZOLI_0443	AZL_005670	AZOBR_160013	AZCBG_440011
AZOLI_0445	AZL_005680	AZOBR_160012	AZCBG_440010
AZOLI_0446	AZL_005690	AZOBR_160011	AZCBG_440009
AZOLI_0448	AZL_005700	AZOBR_160010	AZCBG_440008
AZOLI_0449	AZL_005710	AZOBR_160009	AZCBG_440007
AZOLI_0450	AZL_005720	AZOBR_160007	AZCBG_440006
AZOLI_0451	AZL_005730	AZOBR_160006	AZCBG_440005
AZOLI_0455	AZL_005760	AZOBR_160004	AZCBG_440003
AZOLI_0456	AZL_005770	AZOBR_160003	AZCBG_440002
AZOLI_0457	AZL_005780	AZOBR_160002	AZCBG_440001
AZOLI_0460	AZL_005800	AZOBR_150257	AZCBG_420169
AZOLI_0461	AZL_005810	AZOBR_150256	AZCBG_420168
AZOLI_0462	AZL_005820	AZOBR_150255	AZCBG_420167
AZOLI_0463	AZL_005830	AZOBR_150254	AZCBG_420166
AZOLI_0464	AZL_005840	AZOBR_150253	AZCBG_420165
AZOLI_0465	AZL_005850	AZOBR_150252	AZCBG_420164
AZOLI_0466	AZL_005860	AZOBR_150251	AZCBG_420163
AZOLI_0467	AZL_005870	AZOBR_150250	AZCBG_420162
AZOLI_0468	AZL_005880	AZOBR_150249	AZCBG_420161
AZOLI_0469	AZL_005890	AZOBR_150248	AZCBG_420160
AZOLI_0470	AZL_005900	AZOBR_150247	AZCBG_420159
AZOLI_0471	AZL_005910	AZOBR_150246	AZCBG_420158
AZOLI_0472	AZL_005920	AZOBR_150245	AZCBG_420157
AZOLI_0473	AZL_005930	AZOBR_150244	AZCBG_420156
AZOLI_0474	AZL_005940	AZOBR_150243	AZCBG_420155
AZOLI_0476	AZL_005960	AZOBR_150240	AZCBG_420153
AZOLI_0477	AZL_005970	AZOBR_150239	AZCBG_420152
AZOLI_0479	AZL_005990	AZOBR_150237	AZCBG_420150
AZOLI_0480	AZL_006000	AZOBR_150236	AZCBG_420149
AZOLI_0481	AZL_006010	AZOBR_150235	AZCBG_420148
AZOLI_0482	AZL_006020	AZOBR_150234	AZCBG_420147
AZOLI_0483	AZL_006030	AZOBR_150233	AZCBG_420146
AZOLI_0484	AZL_006040	AZOBR_150232	AZCBG_420145
AZOLI_0486	AZL_006060	AZOBR_150231	AZCBG_420144
AZOLI_0487	AZL_006070	AZOBR_150230	AZCBG_420143
AZOLI_0488	AZL_006110	AZOBR_150229	AZCBG_420142

AZOLI_0489	AZL_006120	AZOBR_150228	AZCBG_420141
AZOLI_0490	AZL_006130	AZOBR_150227	AZCBG_420140
AZOLI_0491	AZL_006140	AZOBR_40335	AZCBG_190052
AZOLI_0492	AZL_006150	AZOBR_10028	AZCBG_20011
AZOLI_0497	AZL_006180	AZOBR_10016	AZCBG_10072
AZOLI_0498	AZL_006190	AZOBR_10015	AZCBG_10071
AZOLI_0501	AZL_0684	AZOBR_10014	AZCBG_10070
AZOLI_0502	AZL_006200	AZOBR_10013	AZCBG_10069
AZOLI_0505	AZL_006220	AZOBR_10010	AZCBG_10066
AZOLI_0506	AZL_0689	AZOBR_70016	AZCBG_220090
AZOLI_0507	AZL_006230	AZOBR_70017	AZCBG_220091
AZOLI_0508	AZL_006240	AZOBR_70019	AZCBG_220092
AZOLI_0510	AZL_006260	AZOBR_70023	AZCBG_220095
AZOLI_0511	AZL_006270	AZOBR_70024	AZCBG_220096
AZOLI_0512	AZL_006280	AZOBR_180163	AZCBG_490069
AZOLI_0513	AZL_006290	AZOBR_180162	AZCBG_490068
AZOLI_0514	AZL_006300	AZOBR_180160	AZCBG_490066
AZOLI_0515	AZL_006310	AZOBR_180158	AZCBG_490065
AZOLI_0516	AZL_006320	AZOBR_40389	AZCBG_210004
AZOLI_0519	AZL_006340	AZOBR_40386	AZCBG_210002
AZOLI_0520	AZL_006350	AZOBR_40385	AZCBG_210001
AZOLI_0522	AZL_006370	AZOBR_180016	AZCBG_480039
AZOLI_0523	AZL_006380	AZOBR_180017	AZCBG_480040
AZOLI_0524	AZL_006390	AZOBR_180018	AZCBG_480041
AZOLI_0529	AZL_006410	AZOBR_180020	AZCBG_480043
AZOLI_0530	AZL_006420	AZOBR_180021	AZCBG_480044
AZOLI_0531	AZL_006430	AZOBR_180022	AZCBG_480045
AZOLI_0535	AZL_006470	AZOBR_70108	AZCBG_230117
AZOLI_0536	AZL_006480	AZOBR_70107	AZCBG_230116
AZOLI_0537	AZL_006490	AZOBR_70106	AZCBG_230115
AZOLI_0538	AZL_006500	AZOBR_70105	AZCBG_230114
AZOLI_0540	AZL_0721	AZOBR_70104	AZCBG_230113
AZOLI_0541	AZL_006520	AZOBR_70109	AZCBG_230118
AZOLI_0542	AZL_006530	AZOBR_70110	AZCBG_230119
AZOLI_0543	AZL_006540	AZOBR_70111	AZCBG_230120
AZOLI_0545	AZL_006560	AZOBR_70113	AZCBG_230122
AZOLI_0549	AZL_006590	AZOBR_40371	AZCBG_200001
AZOLI_0551	AZL_006610	AZOBR_40373	AZCBG_200003
AZOLI_0552	AZL_006620	AZOBR_40375	AZCBG_200005
AZOLI_0553	AZL_006630	AZOBR_40376	AZCBG_200006
AZOLI_0554	AZL_006640	AZOBR_40390	AZCBG_210005
AZOLI_0555	AZL_006650	AZOBR_40391	AZCBG_210006
AZOLI_0556	AZL_006660	AZOBR_10009	AZCBG_10065
AZOLI_0558	AZL_006680	AZOBR_10006	AZCBG_10062
AZOLI_0560	AZL_006690	AZOBR_10005	AZCBG_10061
AZOLI_0561	AZL_006700	AZOBR_10004	AZCBG_10060
AZOLI_0562	AZL_006710	AZOBR_10003	AZCBG_10059
AZOLI_0564	AZL_006730	AZOBR_10046	AZCBG_20025
AZOLI_0565	AZL_006740	AZOBR_10045	AZCBG_20024
AZOLI_0567	AZL_006760	AZOBR_40044	AZCBG_160006
AZOLI_0570	AZL_006780	AZOBR_40047	AZCBG_160009
AZOLI_0574	AZL_006820	AZOBR_200218	AZCBG_10048
AZOLI_0575	AZL_006830	AZOBR_200217	AZCBG_10047
AZOLI_0576	AZL_006840	AZOBR_200216	AZCBG_10046
AZOLI_0578	AZL_006860	AZOBR_70039	AZCBG_230055
AZOLI_0588	AZL_006890	AZOBR_70042	AZCBG_230058
AZOLI_0589	AZL_006900	AZOBR_70043	AZCBG_230059
AZOLI_0590	AZL_006910	AZOBR_70044	AZCBG_230060
AZOLI_0591	AZL_006920	AZOBR_70045	AZCBG_230061

AZOLI_0593	AZL_006940	AZOBR_180150	AZCBG_490059
AZOLI_0597	AZL_006970	AZOBR_180145	AZCBG_490056
AZOLI_0598	AZL_006980	AZOBR_180142	AZCBG_490054
AZOLI_0599	AZL_006990	AZOBR_180141	AZCBG_490053
AZOLI_0602	AZL_007010	AZOBR_70008	AZCBG_220082
AZOLI_0605	AZL_007020	AZOBR_70010	AZCBG_220084
AZOLI_0606	AZL_007030	AZOBR_70011	AZCBG_220085
AZOLI_0607	AZL_007040	AZOBR_70012	AZCBG_220086
AZOLI_0608	AZL_007050	AZOBR_70013	AZCBG_220087
AZOLI_0609	AZL_007060	AZOBR_70014	AZCBG_220088
AZOLI_0610	AZL_007090	AZOBR_p1120031	AZCBG_p140047
AZOLI_0611	AZL_007100	AZOBR_p1120032	AZCBG_p140048
AZOLI_0612	AZL_007110	AZOBR_p1120033	AZCBG_p140049
AZOLI_0613	AZL_007120	AZOBR_p1120034	AZCBG_p140050
AZOLI_0615	AZL_007130	AZOBR_70015	AZCBG_220089
AZOLI_0618	AZL_007160	AZOBR_70031	AZCBG_480057
AZOLI_0619	AZL_007170	AZOBR_70032	AZCBG_480056
AZOLI_0620	AZL_007180	AZOBR_70033	AZCBG_480055
AZOLI_0621	AZL_007190	AZOBR_70034	AZCBG_480054
AZOLI_0622	AZL_007200	AZOBR_70037	AZCBG_480051
AZOLI_0623	AZL_007210	AZOBR_180155	AZCBG_490063
AZOLI_0626	AZL_007240	AZOBR_70046	AZCBG_230062
AZOLI_0627	AZL_007250	AZOBR_70047	AZCBG_230063
AZOLI_0628	AZL_007260	AZOBR_70048	AZCBG_230064
AZOLI_0629	AZL_007270	AZOBR_70049	AZCBG_230065
AZOLI_0630	AZL_007280	AZOBR_70050	AZCBG_230066
AZOLI_0634	AZL_007310	AZOBR_70054	AZCBG_230070
AZOLI_0635	AZL_007320	AZOBR_70055	AZCBG_230071
AZOLI_0638	AZL_007340	AZOBR_70062	AZCBG_230076
AZOLI_0639	AZL_007350	AZOBR_70063	AZCBG_230077
AZOLI_0640	AZL_007360	AZOBR_70064	AZCBG_230078
AZOLI_0641	AZL_007370	AZOBR_70065	AZCBG_230079
AZOLI_0642	AZL_007380	AZOBR_70066	AZCBG_230080
AZOLI_0643	AZL_007390	AZOBR_70067	AZCBG_230081
AZOLI_0644	AZL_007400	AZOBR_70068	AZCBG_230082
AZOLI_0647	AZL_007420	AZOBR_70071	AZCBG_230084
AZOLI_0648	AZL_007430	AZOBR_70073	AZCBG_230086
AZOLI_0649	AZL_007440	AZOBR_70074	AZCBG_230087
AZOLI_0652	AZL_007450	AZOBR_70077	AZCBG_230088
AZOLI_0653	AZL_007460	AZOBR_70078	AZCBG_230089
AZOLI_0656	AZL_007490	AZOBR_70082	AZCBG_230092
AZOLI_0657	AZL_007500	AZOBR_70083	AZCBG_230093
AZOLI_0658	AZL_007510	AZOBR_70085	AZCBG_230094
AZOLI_0659	AZL_007520	AZOBR_70086	AZCBG_230095
AZOLI_0661	AZL_007540	AZOBR_70091	AZCBG_230100
AZOLI_0662	AZL_007550	AZOBR_180007	AZCBG_480032
AZOLI_0663	AZL_007560	AZOBR_180006	AZCBG_480031
AZOLI_0664	AZL_007570	AZOBR_180005	AZCBG_480030
AZOLI_0666	AZL_007590	AZOBR_180004	AZCBG_480029
AZOLI_0667	AZL_007600	AZOBR_180003	AZCBG_480028
AZOLI_0669	AZL_007610	AZOBR_70117	AZCBG_230126
AZOLI_0671	AZL_007630	AZOBR_70119	AZCBG_230128
AZOLI_0672	AZL_007640	AZOBR_70120	AZCBG_230129
AZOLI_0673	AZL_007650	AZOBR_70121	AZCBG_230130
AZOLI_0674	AZL_007660	AZOBR_70122	AZCBG_230131
AZOLI_0679	AZL_007690	AZOBR_70126	AZCBG_230135
AZOLI_0680	AZL_007700	AZOBR_70127	AZCBG_230136
AZOLI_0681	AZL_007710	AZOBR_70128	AZCBG_230137
AZOLI_0683	AZL_007720	AZOBR_70130	AZCBG_230138

AZOLI_0684	AZL_007730	AZOBR_70132	AZCBG_230139
AZOLI_0685	AZL_007740	AZOBR_70133	AZCBG_230140
AZOLI_0686	AZL_007750	AZOBR_70134	AZCBG_230141
AZOLI_0687	AZL_007760	AZOBR_70135	AZCBG_230142
AZOLI_0688	AZL_007770	AZOBR_70136	AZCBG_230143
AZOLI_0689	AZL_007780	AZOBR_200189	AZCBG_10021
AZOLI_0691	AZL_008840	AZOBR_200187	AZCBG_10019
AZOLI_0694	AZL_008850	AZOBR_200186	AZCBG_10018
AZOLI_0695	AZL_008860	AZOBR_200184	AZCBG_10016
AZOLI_0696	AZL_008870	AZOBR_200183	AZCBG_10015
AZOLI_0698	AZL_008890	AZOBR_200181	AZCBG_10013
AZOLI_0699	AZL_008900	AZOBR_200179	AZCBG_10011
AZOLI_0705	AZL_008950	AZOBR_200175	AZCBG_10007
AZOLI_0706	AZL_008960	AZOBR_200168	AZCBG_10002
AZOLI_0707	AZL_008970	AZOBR_200172	AZCBG_10004
AZOLI_0730	AZL_009010	AZOBR_p250007	AZCBG_p2140007
AZOLI_0731	AZL_009030	AZOBR_50039	AZCBG_p360082
AZOLI_0733	AZL_009050	AZOBR_50037	AZCBG_p360081
AZOLI_0734	AZL_009060	AZOBR_50036	AZCBG_p360080
AZOLI_0735	AZL_009070	AZOBR_50035	AZCBG_p360079
AZOLI_0736	AZL_009080	AZOBR_50034	AZCBG_p360078
AZOLI_0737	AZL_009090	AZOBR_50033	AZCBG_p360077
AZOLI_0739	AZL_009100	AZOBR_180081	AZCBG_230001
AZOLI_0744	AZL_009120	AZOBR_140120	AZCBG_350103
AZOLI_0746	AZL_009130	AZOBR_140121	AZCBG_350104
AZOLI_0750	AZL_009140	AZOBR_140122	AZCBG_350105
AZOLI_0752	AZL_009160	AZOBR_140123	AZCBG_350106
AZOLI_0753	AZL_009170	AZOBR_140124	AZCBG_350107
AZOLI_0754	AZL_009180	AZOBR_140125	AZCBG_350108
AZOLI_0755	AZL_009190	AZOBR_140126	AZCBG_350109
AZOLI_0758	AZL_009220	AZOBR_140129	AZCBG_350111
AZOLI_0762	AZL_009260	AZOBR_140131	AZCBG_350113
AZOLI_0763	AZL_009270	AZOBR_140132	AZCBG_350114
AZOLI_0764	AZL_009280	AZOBR_140133	AZCBG_350115
AZOLI_0770	AZL_009330	AZOBR_140267	AZCBG_370066
AZOLI_0771	AZL_009340	AZOBR_140266	AZCBG_370065
AZOLI_0773	AZL_009350	AZOBR_140265	AZCBG_370063
AZOLI_0774	AZL_009360	AZOBR_140263	AZCBG_370061
AZOLI_0775	AZL_009370	AZOBR_140262	AZCBG_370060
AZOLI_0776	AZL_009390	AZOBR_140261	AZCBG_370059
AZOLI_0781	AZL_009410	AZOBR_140156	AZCBG_350136
AZOLI_0782	AZL_009420	AZOBR_140145	AZCBG_350125
AZOLI_0783	AZL_009430	AZOBR_140144	AZCBG_350124
AZOLI_0785	AZL_009450	AZOBR_140140	AZCBG_350122
AZOLI_0786	AZL_009460	AZOBR_140139	AZCBG_350121
AZOLI_0788	AZL_009480	AZOBR_140134	AZCBG_350116
AZOLI_0793	AZL_009510	AZOBR_p130078	AZCBG_p160453
AZOLI_0796	AZL_009530	AZOBR_150048	AZCBG_410007
AZOLI_0797	AZL_009540	AZOBR_150047	AZCBG_410006
AZOLI_0798	AZL_009550	AZOBR_150046	AZCBG_410005
AZOLI_0799	AZL_009560	AZOBR_150027	AZCBG_390015
AZOLI_0800	AZL_009570	AZOBR_150026	AZCBG_390014
AZOLI_0802	AZL_009590	AZOBR_150023	AZCBG_390012
AZOLI_0803	AZL_009600	AZOBR_150022	AZCBG_390011
AZOLI_0805	AZL_009610	AZOBR_150021	AZCBG_390010
AZOLI_0807	AZL_009620	AZOBR_150019	AZCBG_390008
AZOLI_0808	AZL_009630	AZOBR_150018	AZCBG_390007
AZOLI_0809	AZL_009640	AZOBR_150017	AZCBG_390006
AZOLI_0810	AZL_009650	AZOBR_150016	AZCBG_390005

AZOLI_0813	AZL_009680	AZOBR_150012	AZCBG_390002
AZOLI_0816	AZL_009720	AZOBR_140002	AZCBG_340002
AZOLI_0820	AZL_1085	AZOBR_140004	AZCBG_340003
AZOLI_0821	AZL_009730	AZOBR_140005	AZCBG_340004
AZOLI_0822	AZL_009740	AZOBR_140006	AZCBG_340005
AZOLI_0823	AZL_009750	AZOBR_140007	AZCBG_340006
AZOLI_0824	AZL_009760	AZOBR_140009	AZCBG_340007
AZOLI_0826	AZL_009780	AZOBR_140011	AZCBG_350002
AZOLI_0827	AZL_009790	AZOBR_140012	AZCBG_350003
AZOLI_0828	AZL_009800	AZOBR_140013	AZCBG_350004
AZOLI_0829	AZL_009810	AZOBR_140014	AZCBG_350005
AZOLI_0832	AZL_009840	AZOBR_140017	AZCBG_350008
AZOLI_0833	AZL_009850	AZOBR_140018	AZCBG_350009
AZOLI_0835	AZL_009860	AZOBR_100326	AZCBG_320006
AZOLI_0837	AZL_009880	AZOBR_100329	AZCBG_320008
AZOLI_0838	AZL_009900	AZOBR_150089	AZCBG_420013
AZOLI_0839	AZL_009910	AZOBR_150088	AZCBG_420011
AZOLI_0840	AZL_009920	AZOBR_150087	AZCBG_420010
AZOLI_0845	AZL_009950	AZOBR_140052	AZCBG_350040
AZOLI_0846	AZL_009960	AZOBR_140053	AZCBG_350041
AZOLI_0847	AZL_009970	AZOBR_140054	AZCBG_350042
AZOLI_0848	AZL_022850	AZOBR_140057	AZCBG_350045
AZOLI_0849	AZL_022840	AZOBR_140058	AZCBG_350046
AZOLI_0851	AZL_009980	AZOBR_p1130023	AZCBG_p150058
AZOLI_0852	AZL_009990	AZOBR_p1170073	AZCBG_p160128
AZOLI_0853	AZL_010010	AZOBR_140228	AZCBG_370029
AZOLI_0854	AZL_010020	AZOBR_140230	AZCBG_370031
AZOLI_0856	AZL_010040	AZOBR_140232	AZCBG_370033
AZOLI_0859	AZL_010070	AZOBR_140235	AZCBG_370036
AZOLI_0861	AZL_010080	AZOBR_140238	AZCBG_370037
AZOLI_0862	AZL_010090	AZOBR_140239	AZCBG_370038
AZOLI_0863	AZL_010100	AZOBR_140240	AZCBG_370039
AZOLI_0864	AZL_010110	AZOBR_140248	AZCBG_370047
AZOLI_0867	AZL_010130	AZOBR_140246	AZCBG_370045
AZOLI_0869	AZL_010150	AZOBR_140244	AZCBG_370043
AZOLI_0870	AZL_010160	AZOBR_140243	AZCBG_370042
AZOLI_0871	AZL_010170	AZOBR_140242	AZCBG_370041
AZOLI_0872	AZL_010180	AZOBR_140241	AZCBG_370040
AZOLI_0873	AZL_010190	AZOBR_140249	AZCBG_370048
AZOLI_0874	AZL_010200	AZOBR_140250	AZCBG_370049
AZOLI_0876	AZL_010220	AZOBR_140253	AZCBG_370051
AZOLI_0877	AZL_010230	AZOBR_140254	AZCBG_370052
AZOLI_0878	AZL_010240	AZOBR_140255	AZCBG_370053
AZOLI_0880	AZL_010250	AZOBR_140256	AZCBG_370054
AZOLI_0918	AZL_010450	AZOBR_p220076	AZCBG_p2100004
AZOLI_0919	AZL_010460	AZOBR_p220075	AZCBG_p2100003
AZOLI_0920	AZL_010470	AZOBR_150141	AZCBG_420060
AZOLI_0922	AZL_010480	AZOBR_150140	AZCBG_420059
AZOLI_0923	AZL_010490	AZOBR_150139	AZCBG_420058
AZOLI_0924	AZL_010500	AZOBR_150137	AZCBG_420057
AZOLI_0928	AZL_010540	AZOBR_150132	AZCBG_420052
AZOLI_0929	AZL_010550	AZOBR_150130	AZCBG_420051
AZOLI_0946	AZL_010590	AZOBR_100064	AZCBG_270077
AZOLI_0948	AZL_010610	AZOBR_180059	AZCBG_230022
AZOLI_0951	AZL_010630	AZOBR_180055	AZCBG_230025
AZOLI_0966	AZL_010720	AZOBR_180034	AZCBG_230044
AZOLI_0968	AZL_010730	AZOBR_180032	AZCBG_230046
AZOLI_0969	AZL_010740	AZOBR_180031	AZCBG_230047
AZOLI_0970	AZL_010750	AZOBR_180030	AZCBG_230048

AZOLI_0979	AZL_1202	AZOBR_90007	AZCBG_270007
AZOLI_0981	AZL_010790	AZOBR_90009	AZCBG_270009
AZOLI_0982	AZL_010800	AZOBR_90010	AZCBG_270010
AZOLI_0984	AZL_010820	AZOBR_150105	AZCBG_420030
AZOLI_0986	AZL_010830	AZOBR_150104	AZCBG_420029
AZOLI_0987	AZL_010840	AZOBR_150102	AZCBG_420028
AZOLI_0988	AZL_010850	AZOBR_150101	AZCBG_420027
AZOLI_0990	AZL_018050	AZOBR_p130163	AZCBG_p160548
AZOLI_0991	AZL_018040	AZOBR_p130162	AZCBG_p160547
AZOLI_0993	AZL_018020	AZOBR_100360	AZCBG_320035
AZOLI_1015	AZL_017820	AZOBR_p1140127	AZCBG_p150251
AZOLI_1034	AZL_017620	AZOBR_p110065	AZCBG_p160245
AZOLI_1036	AZL_012360	AZOBR_100311	AZCBG_310040
AZOLI_1037	AZL_012370	AZOBR_100312	AZCBG_310041
AZOLI_1046	AZL_012420	AZOBR_p1170039	AZCBG_p160097
AZOLI_1047	AZL_012430	AZOBR_40107	AZCBG_160066
AZOLI_1048	AZL_012440	AZOBR_p120110	AZCBG_p160362
AZOLI_1050	AZL_012460	AZOBR_p1170045	AZCBG_p160103
AZOLI_1053	AZL_012490	AZOBR_p1170040	AZCBG_p160098
AZOLI_1054	AZL_012500	AZOBR_40105	AZCBG_160064
AZOLI_1055	AZL_012510	AZOBR_140217	AZCBG_370019
AZOLI_1056	AZL_012520	AZOBR_140219	AZCBG_370020
AZOLI_1057	AZL_012530	AZOBR_140220	AZCBG_370021
AZOLI_1059	AZL_016350	AZOBR_100156	AZCBG_300028
AZOLI_1063	AZL_016320	AZOBR_p130170	AZCBG_p160555
AZOLI_1064	AZL_016310	AZOBR_p130169	AZCBG_p160554
AZOLI_1065	AZL_016300	AZOBR_p130168	AZCBG_p160553
AZOLI_1066	AZL_016290	AZOBR_p130145	AZCBG_p160534
AZOLI_1068	AZL_016280	AZOBR_p130144	AZCBG_p160533
AZOLI_1069	AZL_016270	AZOBR_p130143	AZCBG_p160532
AZOLI_1070	AZL_016260	AZOBR_100274	AZCBG_310007
AZOLI_1072	AZL_016240	AZOBR_100277	AZCBG_310009
AZOLI_1073	AZL_016230	AZOBR_100278	AZCBG_310010
AZOLI_1074	AZL_016220	AZOBR_100279	AZCBG_310011
AZOLI_1075	AZL_016210	AZOBR_100280	AZCBG_310012
AZOLI_1076	AZL_016200	AZOBR_100281	AZCBG_310013
AZOLI_1077	AZL_016190	AZOBR_100282	AZCBG_310014
AZOLI_1078	AZL_016180	AZOBR_100283	AZCBG_310015
AZOLI_1080	AZL_016160	AZOBR_100285	AZCBG_310017
AZOLI_1081	AZL_016150	AZOBR_100286	AZCBG_310018
AZOLI_1082	AZL_016140	AZOBR_100287	AZCBG_310019
AZOLI_1083	AZL_016130	AZOBR_100288	AZCBG_310020
AZOLI_1084	AZL_016120	AZOBR_100289	AZCBG_310021
AZOLI_1089	AZL_016080	AZOBR_100296	AZCBG_310027
AZOLI_1091	AZL_016060	AZOBR_100299	AZCBG_310029
AZOLI_1092	AZL_016050	AZOBR_100300	AZCBG_310030
AZOLI_1093	AZL_016040	AZOBR_100301	AZCBG_310031
AZOLI_1094	AZL_016030	AZOBR_100302	AZCBG_310032
AZOLI_1095	AZL_016020	AZOBR_100303	AZCBG_310033
AZOLI_1096	AZL_016010	AZOBR_100304	AZCBG_310034
AZOLI_1097	AZL_016000	AZOBR_100305	AZCBG_310035
AZOLI_1098	AZL_015990	AZOBR_100306	AZCBG_310036
AZOLI_1101	AZL_015960	AZOBR_110020	AZCBG_330019
AZOLI_1102	AZL_013940	AZOBR_110113	AZCBG_330107
AZOLI_1103	AZL_013950	AZOBR_110114	AZCBG_330108
AZOLI_1104	AZL_013960	AZOBR_110115	AZCBG_330109
AZOLI_1108	AZL_014000	AZOBR_110118	AZCBG_330111
AZOLI_1109	AZL_014010	AZOBR_110119	AZCBG_330112
AZOLI_1110	AZL_014030	AZOBR_110120	AZCBG_330113

AZOLI_1111	AZL_014040	AZOBR_110121	AZCBG_330114
AZOLI_1112	AZL_014050	AZOBR_110122	AZCBG_330115
AZOLI_1115	AZL_014060	AZOBR_110123	AZCBG_330116
AZOLI_1116	AZL_014070	AZOBR_110125	AZCBG_330117
AZOLI_1119	AZL_014080	AZOBR_110128	AZCBG_330120
AZOLI_1123	AZL_014120	AZOBR_p1170052	AZCBG_p160110
AZOLI_1126	AZL_014140	AZOBR_p1170055	AZCBG_p160112
AZOLI_1128	AZL_014150	AZOBR_p110089	AZCBG_p160267
AZOLI_1129	AZL_1593	AZOBR_p110088	AZCBG_p160266
AZOLI_1131	AZL_014190	AZOBR_p110086	AZCBG_p160265
AZOLI_1134	AZL_014210	AZOBR_p110080	AZCBG_p160261
AZOLI_1135	AZL_014220	AZOBR_p110079	AZCBG_p160260
AZOLI_1137	AZL_014230	AZOBR_p110077	AZCBG_p160258
AZOLI_1138	AZL_014240	AZOBR_p110076	AZCBG_p160257
AZOLI_1141	AZL_014270	AZOBR_p1180013	AZCBG_p160162
AZOLI_1143	AZL_014300	AZOBR_p1180014	AZCBG_p160164
AZOLI_1144	AZL_014310	AZOBR_p1180015	AZCBG_p160165
AZOLI_1145	AZL_014320	AZOBR_p1180016	AZCBG_p160166
AZOLI_1146	AZL_d00940	AZOBR_p330005	AZCBG_p360059
AZOLI_1150	AZL_014340	AZOBR_p1180018	AZCBG_p160168
AZOLI_1151	AZL_014350	AZOBR_p1180019	AZCBG_p160169
AZOLI_1155	AZL_014390	AZOBR_p1180023	AZCBG_p160173
AZOLI_1156	AZL_014400	AZOBR_p1180024	AZCBG_p160174
AZOLI_1157	AZL_014420	AZOBR_p1180025	AZCBG_p160175
AZOLI_1158	AZL_014430	AZOBR_140285	AZCBG_370083
AZOLI_1160	AZL_014440	AZOBR_140284	AZCBG_370082
AZOLI_1161	AZL_014450	AZOBR_140281	AZCBG_370079
AZOLI_1162	AZL_014460	AZOBR_140280	AZCBG_370078
AZOLI_1165	AZL_017100	AZOBR_140308	AZCBG_370103
AZOLI_1166	AZL_017090	AZOBR_140309	AZCBG_370104
AZOLI_1182	AZL_017060	AZOBR_110094	AZCBG_330090
AZOLI_1183	AZL_017050	AZOBR_110093	AZCBG_330089
AZOLI_1185	AZL_017020	AZOBR_110092	AZCBG_330088
AZOLI_1186	AZL_017010	AZOBR_110091	AZCBG_330087
AZOLI_1187	AZL_017000	AZOBR_110090	AZCBG_330086
AZOLI_1188	AZL_016990	AZOBR_110089	AZCBG_330085
AZOLI_1189	AZL_1464	AZOBR_p130161	AZCBG_p160546
AZOLI_1190	AZL_013090	AZOBR_p130160	AZCBG_p160545
AZOLI_1191	AZL_013100	AZOBR_p130159	AZCBG_p160544
AZOLI_1193	AZL_013120	AZOBR_p130157	AZCBG_p160542
AZOLI_1194	AZL_013130	AZOBR_p130156	AZCBG_p160541
AZOLI_1195	AZL_013140	AZOBR_p130155	AZCBG_p160540
AZOLI_1196	AZL_013150	AZOBR_p130154	AZCBG_p160539
AZOLI_1197	AZL_013160	AZOBR_140078	AZCBG_350069
AZOLI_1200	AZL_013180	AZOBR_110044	AZCBG_330041
AZOLI_1204	AZL_013210	AZOBR_110049	AZCBG_330046
AZOLI_1206	AZL_013230	AZOBR_110051	AZCBG_330048
AZOLI_1208	AZL_013250	AZOBR_110053	AZCBG_330050
AZOLI_1209	AZL_013260	AZOBR_110054	AZCBG_330051
AZOLI_1212	AZL_013290	AZOBR_110057	AZCBG_330053
AZOLI_1215	AZL_013310	AZOBR_110060	AZCBG_330055
AZOLI_1216	AZL_013320	AZOBR_110061	AZCBG_330056
AZOLI_1217	AZL_013330	AZOBR_100078	AZCBG_270088
AZOLI_1218	AZL_013630	AZOBR_p130198	AZCBG_p160584
AZOLI_1219	AZL_013640	AZOBR_p130197	AZCBG_p160583
AZOLI_1221	AZL_013660	AZOBR_p130194	AZCBG_p160578
AZOLI_1224	AZL_013700	AZOBR_100316	AZCBG_310044
AZOLI_1225	AZL_013720	AZOBR_100317	AZCBG_310045
AZOLI_1226	AZL_013740	AZOBR_100318	AZCBG_310046

AZOLI_1232	AZL_013800	AZOBR_120036	AZCBG_330147
AZOLI_1234	AZL_013910	AZOBR_p120068	AZCBG_p160325
AZOLI_1235	AZL_013920	AZOBR_p120069	AZCBG_p160326
AZOLI_1236	AZL_013930	AZOBR_p120071	AZCBG_p160327
AZOLI_1239	AZL_015950	AZOBR_100362	AZCBG_320037
AZOLI_1241	AZL_015940	AZOBR_100364	AZCBG_320039
AZOLI_1242	AZL_015930	AZOBR_140190	AZCBG_360020
AZOLI_1243	AZL_015920	AZOBR_140191	AZCBG_360021
AZOLI_1244	AZL_015910	AZOBR_140192	AZCBG_360022
AZOLI_1245	AZL_015900	AZOBR_150037	AZCBG_400008
AZOLI_1246	AZL_015890	AZOBR_150035	AZCBG_400007
AZOLI_1252	AZL_015880	AZOBR_150031	AZCBG_400004
AZOLI_1253	AZL_015870	AZOBR_150030	AZCBG_400003
AZOLI_1254	AZL_015860	AZOBR_150029	AZCBG_400002
AZOLI_1255	AZL_015850	AZOBR_150028	AZCBG_400001
AZOLI_1257	AZL_015840	AZOBR_150118	AZCBG_420041
AZOLI_1258	AZL_015830	AZOBR_140099	AZCBG_350086
AZOLI_1261	AZL_015800	AZOBR_p130191	AZCBG_p160573
AZOLI_1263	AZL_015780	AZOBR_p130173	AZCBG_p160558
AZOLI_1264	AZL_015770	AZOBR_130034	AZCBG_330212
AZOLI_1266	AZL_015750	AZOBR_130032	AZCBG_330209
AZOLI_1271	AZL_015730	AZOBR_130029	AZCBG_330206
AZOLI_1274	AZL_015720	AZOBR_130027	AZCBG_330203
AZOLI_1275	AZL_015710	AZOBR_130026	AZCBG_330202
AZOLI_1276	AZL_015700	AZOBR_130025	AZCBG_330201
AZOLI_1277	AZL_015690	AZOBR_130024	AZCBG_330200
AZOLI_1278	AZL_015680	AZOBR_p140024	AZCBG_p170081
AZOLI_1280	AZL_015640	AZOBR_p1130183	AZCBG_p150208
AZOLI_1281	AZL_015630	AZOBR_p1130184	AZCBG_p150209
AZOLI_1282	AZL_015620	AZOBR_p1130187	AZCBG_p150212
AZOLI_1289	AZL_015550	AZOBR_100097	AZCBG_290002
AZOLI_1290	AZL_015540	AZOBR_100096	AZCBG_290001
AZOLI_1291	AZL_015530	AZOBR_100095	AZCBG_280012
AZOLI_1292	AZL_015520	AZOBR_100093	AZCBG_280010
AZOLI_1296	AZL_015510	AZOBR_130045	AZCBG_330222
AZOLI_1297	AZL_015500	AZOBR_130044	AZCBG_330221
AZOLI_1298	AZL_015490	AZOBR_130042	AZCBG_330220
AZOLI_1299	AZL_015480	AZOBR_130041	AZCBG_330219
AZOLI_1300	AZL_015470	AZOBR_130040	AZCBG_330218
AZOLI_1302	AZL_015450	AZOBR_130039	AZCBG_330217
AZOLI_1303	AZL_015440	AZOBR_130038	AZCBG_330216
AZOLI_1304	AZL_015430	AZOBR_130037	AZCBG_330215
AZOLI_1305	AZL_015420	AZOBR_150044	AZCBG_410003
AZOLI_1306	AZL_015410	AZOBR_150043	AZCBG_410002
AZOLI_1307	AZL_015400	AZOBR_140137	AZCBG_350119
AZOLI_1308	AZL_015390	AZOBR_150042	AZCBG_410001
AZOLI_1310	AZL_015380	AZOBR_150040	AZCBG_400010
AZOLI_1311	AZL_015370	AZOBR_150039	AZCBG_400009
AZOLI_1312	AZL_015360	AZOBR_140102	AZCBG_350088
AZOLI_1313	AZL_015350	AZOBR_140103	AZCBG_350089
AZOLI_1314	AZL_015340	AZOBR_140104	AZCBG_350090
AZOLI_1315	AZL_015330	AZOBR_100148	AZCBG_300021
AZOLI_1316	AZL_015320	AZOBR_100149	AZCBG_300022
AZOLI_1317	AZL_015310	AZOBR_100150	AZCBG_300023
AZOLI_1320	AZL_015290	AZOBR_100152	AZCBG_300025
AZOLI_1321	AZL_015280	AZOBR_100153	AZCBG_300026
AZOLI_1322	AZL_015270	AZOBR_100154	AZCBG_300027
AZOLI_1325	AZL_015240	AZOBR_p1150019	AZCBG_p150278
AZOLI_1328	AZL_015220	AZOBR_p1180035	AZCBG_p160183

AZOLI_1329	AZL_015210	AZOBR_p1180036	AZCBG_p160184
AZOLI_1330	AZL_015200	AZOBR_p1180037	AZCBG_p160185
AZOLI_1333	AZL_015170	AZOBR_p110002	AZCBG_p160187
AZOLI_1335	AZL_015150	AZOBR_p110003	AZCBG_p160188
AZOLI_1336	AZL_015140	AZOBR_p110004	AZCBG_p160189
AZOLI_1337	AZL_015130	AZOBR_p1100049	AZCBG_p1110058
AZOLI_1338	AZL_015120	AZOBR_p110005	AZCBG_p160190
AZOLI_1339	AZL_015110	AZOBR_p110006	AZCBG_p160191
AZOLI_1340	AZL_015100	AZOBR_p110007	AZCBG_p160192
AZOLI_1341	AZL_015090	AZOBR_p110008	AZCBG_p160193
AZOLI_1342	AZL_015080	AZOBR_p110009	AZCBG_p160194
AZOLI_1343	AZL_015070	AZOBR_p110010	AZCBG_p160195
AZOLI_1344	AZL_015060	AZOBR_p110011	AZCBG_p160196
AZOLI_1345	AZL_015050	AZOBR_p110013	AZCBG_p160197
AZOLI_1348	AZL_015020	AZOBR_p110018	AZCBG_p160201
AZOLI_1349	AZL_015010	AZOBR_p110019	AZCBG_p160202
AZOLI_1350	AZL_015000	AZOBR_p110020	AZCBG_p160203
AZOLI_1351	AZL_1688	AZOBR_p110022	AZCBG_p160204
AZOLI_1353	AZL_014980	AZOBR_p110024	AZCBG_p160206
AZOLI_1361	AZL_014900	AZOBR_120033	AZCBG_330144
AZOLI_1366	AZL_014630	AZOBR_p1120067	AZCBG_p140082
AZOLI_1370	AZL_014730	AZOBR_p1130207	AZCBG_p360029
AZOLI_1372	AZL_014750	AZOBR_p1140002	AZCBG_p360031
AZOLI_1375	AZL_014770	AZOBR_p1140004	AZCBG_p360033
AZOLI_1377	AZL_014790	AZOBR_p1140007	AZCBG_p360036
AZOLI_1380	AZL_014820	AZOBR_p130148	AZCBG_p160536
AZOLI_1381	AZL_014830	AZOBR_p130149	AZCBG_p160537
AZOLI_1382	AZL_014840	AZOBR_p130150	AZCBG_p160538
AZOLI_1383	AZL_014850	AZOBR_p120082	AZCBG_p160337
AZOLI_1385	AZL_014620	AZOBR_p120086	AZCBG_p160341
AZOLI_1386	AZL_014600	AZOBR_p120089	AZCBG_p160343
AZOLI_1387	AZL_014590	AZOBR_p120090	AZCBG_p160344
AZOLI_1388	AZL_014580	AZOBR_p120091	AZCBG_p160345
AZOLI_1389	AZL_014570	AZOBR_p120092	AZCBG_p160346
AZOLI_1390	AZL_014560	AZOBR_p120093	AZCBG_p160347
AZOLI_1393	AZL_014550	AZOBR_p120094	AZCBG_p160348
AZOLI_1394	AZL_014540	AZOBR_p120095	AZCBG_p160349
AZOLI_1395	AZL_014530	AZOBR_p120096	AZCBG_p160350
AZOLI_1397	AZL_014510	AZOBR_p120099	AZCBG_p160353
AZOLI_1399	AZL_014490	AZOBR_p120102	AZCBG_p160355
AZOLI_1400	AZL_014480	AZOBR_p120103	AZCBG_p160356
AZOLI_1401	AZL_014470	AZOBR_p120104	AZCBG_p160357
AZOLI_1403	AZL_017170	AZOBR_140305	AZCBG_370100
AZOLI_1406	AZL_017180	AZOBR_140303	AZCBG_370098
AZOLI_1408	AZL_017200	AZOBR_p110145	AZCBG_p160313
AZOLI_1409	AZL_017250	AZOBR_p110146	AZCBG_p160314
AZOLI_1412	AZL_017270	AZOBR_100038	AZCBG_270056
AZOLI_1414	AZL_017300	AZOBR_100036	AZCBG_270054
AZOLI_1418	AZL_017340	AZOBR_100029	AZCBG_270050
AZOLI_1420	AZL_017360	AZOBR_100027	AZCBG_270048
AZOLI_1421	AZL_017370	AZOBR_100025	AZCBG_270046
AZOLI_1422	AZL_017380	AZOBR_100024	AZCBG_270045
AZOLI_1423	AZL_017390	AZOBR_100023	AZCBG_270044
AZOLI_1427	AZL_017420	AZOBR_100018	AZCBG_270038
AZOLI_1428	AZL_017430	AZOBR_100017	AZCBG_270037
AZOLI_1430	AZL_017440	AZOBR_100016	AZCBG_270036
AZOLI_1431	AZL_017450	AZOBR_p270164	AZCBG_p2200016
AZOLI_1432	AZL_017460	AZOBR_100015	AZCBG_270035
AZOLI_1433	AZL_017470	AZOBR_100014	AZCBG_270034

AZOLI_1434	AZL_017480	AZOBR_100013	AZCBG_270032
AZOLI_1437	AZL_017490	AZOBR_100011	AZCBG_270030
AZOLI_1438	AZL_017500	AZOBR_100010	AZCBG_270029
AZOLI_1439	AZL_017510	AZOBR_100009	AZCBG_270028
AZOLI_1442	AZL_017530	AZOBR_100006	AZCBG_270025
AZOLI_1444	AZL_017550	AZOBR_90012	AZCBG_270012
AZOLI_1452	AZL_012340	AZOBR_150053	AZCBG_410011
AZOLI_1453	AZL_012330	AZOBR_150054	AZCBG_410012
AZOLI_1454	AZL_012320	AZOBR_150055	AZCBG_410013
AZOLI_1457	AZL_012300	AZOBR_150058	AZCBG_410016
AZOLI_1458	AZL_012290	AZOBR_150059	AZCBG_410017
AZOLI_1459	AZL_012280	AZOBR_150060	AZCBG_410018
AZOLI_1460	AZL_012270	AZOBR_150061	AZCBG_410019
AZOLI_1461	AZL_012260	AZOBR_150062	AZCBG_410020
AZOLI_1464	AZL_012230	AZOBR_150063	AZCBG_410021
AZOLI_1465	AZL_012220	AZOBR_140035	AZCBG_350025
AZOLI_1467	AZL_012200	AZOBR_140180	AZCBG_360010
AZOLI_1474	AZL_012170	AZOBR_140213	AZCBG_370014
AZOLI_1475	AZL_012160	AZOBR_140212	AZCBG_370013
AZOLI_1476	AZL_012150	AZOBR_140177	AZCBG_360007
AZOLI_1478	AZL_012130	AZOBR_150173	AZCBG_420090
AZOLI_1479	AZL_012120	AZOBR_150176	AZCBG_420092
AZOLI_1480	AZL_012110	AZOBR_150177	AZCBG_420093
AZOLI_1481	AZL_012100	AZOBR_150178	AZCBG_420094
AZOLI_1482	AZL_012090	AZOBR_150179	AZCBG_420095
AZOLI_1483	AZL_012080	AZOBR_150180	AZCBG_420096
AZOLI_1485	AZL_012060	AZOBR_150183	AZCBG_420098
AZOLI_1488	AZL_012040	AZOBR_150187	AZCBG_420103
AZOLI_1489	AZL_012030	AZOBR_150188	AZCBG_420104
AZOLI_1490	AZL_012020	AZOBR_150189	AZCBG_420105
AZOLI_1493	AZL_011990	AZOBR_150192	AZCBG_420107
AZOLI_1495	AZL_011970	AZOBR_150194	AZCBG_420109
AZOLI_1497	AZL_011960	AZOBR_140151	AZCBG_350131
AZOLI_1499	AZL_011860	AZOBR_140150	AZCBG_350130
AZOLI_1503	AZL_011830	AZOBR_p1170093	AZCBG_p160148
AZOLI_1504	AZL_011820	AZOBR_p1170092	AZCBG_p160147
AZOLI_1505	AZL_011810	AZOBR_p1170091	AZCBG_p160146
AZOLI_1506	AZL_011800	AZOBR_p1170089	AZCBG_p160145
AZOLI_1507	AZL_011790	AZOBR_p1170088	AZCBG_p160144
AZOLI_1508	AZL_011780	AZOBR_p1170086	AZCBG_p160142
AZOLI_1510	AZL_011760	AZOBR_p1170084	AZCBG_p160140
AZOLI_1511	AZL_011750	AZOBR_p130179	AZCBG_p160563
AZOLI_1512	AZL_011740	AZOBR_p130180	AZCBG_p160564
AZOLI_1513	AZL_011730	AZOBR_p130181	AZCBG_p160565
AZOLI_1515	AZL_011720	AZOBR_p130109	AZCBG_660010
AZOLI_1516	AZL_011710	AZOBR_p130108	AZCBG_660011
AZOLI_1521	AZL_011110	AZOBR_p110061	AZCBG_p160241
AZOLI_1522	AZL_011100	AZOBR_p110062	AZCBG_p160242
AZOLI_1524	AZL_011080	AZOBR_p440185	AZCBG_p4140006
AZOLI_1525	AZL_011070	AZOBR_p150076	AZCBG_p150003
AZOLI_1527	AZL_011050	AZOBR_p150053	AZCBG_p150021
AZOLI_1528	AZL_011040	AZOBR_p440175	AZCBG_p4130005
AZOLI_1538	AZL_010980	AZOBR_p130019	AZCBG_p160396
AZOLI_1545	AZL_010940	AZOBR_100390	AZCBG_320067
AZOLI_1546	AZL_010930	AZOBR_100389	AZCBG_320066
AZOLI_1547	AZL_010920	AZOBR_100388	AZCBG_320065
AZOLI_1551	AZL_010880	AZOBR_150092	AZCBG_420016
AZOLI_1552	AZL_010870	AZOBR_150093	AZCBG_420017
AZOLI_1554	AZL_018090	AZOBR_p130164	AZCBG_p160549

AZOLI_1555	AZL_018100	AZOBR_p130166	AZCBG_p160550
AZOLI_1556	AZL_018110	AZOBR_p130167	AZCBG_p160552
AZOLI_1557	AZL_017130	AZOBR_10468	AZCBG_100038
AZOLI_1558	AZL_017120	AZOBR_10469	AZCBG_100039
AZOLI_1559	AZL_013080	AZOBR_120063	AZCBG_330175
AZOLI_1562	AZL_013050	AZOBR_120058	AZCBG_330172
AZOLI_1563	AZL_013040	AZOBR_120057	AZCBG_330171
AZOLI_1568	AZL_1457	AZOBR_120054	AZCBG_330168
AZOLI_1570	AZL_013010	AZOBR_120052	AZCBG_330166
AZOLI_1571	AZL_013000	AZOBR_120051	AZCBG_330165
AZOLI_1572	AZL_012990	AZOBR_120050	AZCBG_330164
AZOLI_1573	AZL_012980	AZOBR_120049	AZCBG_330163
AZOLI_1574	AZL_012970	AZOBR_120048	AZCBG_330162
AZOLI_1575	AZL_012960	AZOBR_120047	AZCBG_330161
AZOLI_1576	AZL_012950	AZOBR_120046	AZCBG_330160
AZOLI_1577	AZL_1448	AZOBR_120037	AZCBG_330149
AZOLI_1579	AZL_012940	AZOBR_120044	AZCBG_330158
AZOLI_1580	AZL_012930	AZOBR_120043	AZCBG_330157
AZOLI_1581	AZL_012920	AZOBR_120042	AZCBG_330156
AZOLI_1582	AZL_012910	AZOBR_120041	AZCBG_330155
AZOLI_1583	AZL_012900	AZOBR_120040	AZCBG_330154
AZOLI_1584	AZL_012890	AZOBR_120039	AZCBG_330152
AZOLI_1585	AZL_012880	AZOBR_120038	AZCBG_330151
AZOLI_1591	AZL_012850	AZOBR_p1130058	AZCBG_p150092
AZOLI_1592	AZL_012840	AZOBR_p1130059	AZCBG_p150093
AZOLI_1593	AZL_012820	AZOBR_p1130060	AZCBG_p150094
AZOLI_1595	AZL_012810	AZOBR_p1130061	AZCBG_p150095
AZOLI_1596	AZL_012800	AZOBR_p1130062	AZCBG_p150096
AZOLI_1597	AZL_012790	AZOBR_p1130063	AZCBG_p150097
AZOLI_1598	AZL_012780	AZOBR_p1130064	AZCBG_p150098
AZOLI_1599	AZL_012770	AZOBR_140272	AZCBG_370071
AZOLI_1600	AZL_012760	AZOBR_140273	AZCBG_370072
AZOLI_1601	AZL_012750	AZOBR_140274	AZCBG_370073
AZOLI_1608	AZL_012690	AZOBR_140279	AZCBG_370077
AZOLI_1610	AZL_012670	AZOBR_10460	AZCBG_100030
AZOLI_1611	AZL_012660	AZOBR_10459	AZCBG_100029
AZOLI_1613	AZL_012640	AZOBR_10473	AZCBG_100042
AZOLI_1614	AZL_012630	AZOBR_10474	AZCBG_100043
AZOLI_1616	AZL_012610	AZOBR_140033	AZCBG_350023
AZOLI_1617	AZL_012600	AZOBR_150064	AZCBG_410022
AZOLI_1622	AZL_012570	AZOBR_150069	AZCBG_410027
AZOLI_1623	AZL_012560	AZOBR_140074	AZCBG_350066
AZOLI_1624	AZL_012550	AZOBR_140073	AZCBG_350065
AZOLI_1625	AZL_012540	AZOBR_140072	AZCBG_350064
AZOLI_1626	AZL_016360	AZOBR_p1180026	AZCBG_p160176
AZOLI_1628	AZL_016380	AZOBR_p1180028	AZCBG_p160178
AZOLI_1629	AZL_016390	AZOBR_p1180029	AZCBG_p160179
AZOLI_1631	AZL_016410	AZOBR_p1180032	AZCBG_p160181
AZOLI_1632	AZL_016420	AZOBR_p1180033	AZCBG_p160182
AZOLI_1633	AZL_016430	AZOBR_100354	AZCBG_320031
AZOLI_1634	AZL_016440	AZOBR_100353	AZCBG_320030
AZOLI_1635	AZL_016450	AZOBR_100352	AZCBG_320029
AZOLI_1638	AZL_016470	AZOBR_140048	AZCBG_350037
AZOLI_1639	AZL_016480	AZOBR_140047	AZCBG_350036
AZOLI_1640	AZL_016490	AZOBR_140046	AZCBG_350035
AZOLI_1641	AZL_016500	AZOBR_140045	AZCBG_350034
AZOLI_1642	AZL_016510	AZOBR_140044	AZCBG_350033
AZOLI_1643	AZL_016520	AZOBR_140043	AZCBG_350032
AZOLI_1644	AZL_016530	AZOBR_140042	AZCBG_350031

AZOLI_1645	AZL_016540	AZOBR_140040	AZCBG_350030
AZOLI_1657	AZL_016610	AZOBR_140201	AZCBG_370004
AZOLI_1658	AZL_016620	AZOBR_140194	AZCBG_360023
AZOLI_1669	AZL_016710	AZOBR_110110	AZCBG_330105
AZOLI_1670	AZL_016720	AZOBR_110109	AZCBG_330104
AZOLI_1671	AZL_016730	AZOBR_110108	AZCBG_330103
AZOLI_1674	AZL_016750	AZOBR_110106	AZCBG_330101
AZOLI_1677	AZL_016780	AZOBR_110104	AZCBG_330099
AZOLI_1678	AZL_016790	AZOBR_110103	AZCBG_330098
AZOLI_1679	AZL_016800	AZOBR_110102	AZCBG_330097
AZOLI_1681	AZL_016830	AZOBR_150150	AZCBG_420068
AZOLI_1682	AZL_016840	AZOBR_150151	AZCBG_420069
AZOLI_1687	AZL_016870	AZOBR_150153	AZCBG_420071
AZOLI_1689	AZL_016890	AZOBR_150155	AZCBG_420073
AZOLI_1690	AZL_016900	AZOBR_150156	AZCBG_420074
AZOLI_1703	AZL_016960	AZOBR_150163	AZCBG_420080
AZOLI_1704	AZL_016970	AZOBR_150164	AZCBG_420081
AZOLI_1705	AZL_016980	AZOBR_150165	AZCBG_420082
AZOLI_1713	AZL_017160	AZOBR_10466	AZCBG_100035
AZOLI_1718	AZL_018130	AZOBR_120065	AZCBG_330177
AZOLI_1722	AZL_018160	AZOBR_130003	AZCBG_330180
AZOLI_1723	AZL_018170	AZOBR_130004	AZCBG_330181
AZOLI_1724	AZL_018180	AZOBR_130005	AZCBG_330182
AZOLI_1725	AZL_018190	AZOBR_130006	AZCBG_330183
AZOLI_1726	AZL_018200	AZOBR_130007	AZCBG_330184
AZOLI_1727	AZL_018210	AZOBR_p1130057	AZCBG_p150091
AZOLI_1728	AZL_018250	AZOBR_p1130051	AZCBG_p150087
AZOLI_1729	AZL_018260	AZOBR_p1130052	AZCBG_p150088
AZOLI_1731	AZL_018270	AZOBR_p1130054	AZCBG_p150089
AZOLI_1732	AZL_018280	AZOBR_p1130056	AZCBG_p150090
AZOLI_1735	AZL_018310	AZOBR_150198	AZCBG_420113
AZOLI_1736	AZL_018320	AZOBR_150199	AZCBG_420114
AZOLI_1738	AZL_018330	AZOBR_150201	AZCBG_420116
AZOLI_1739	AZL_018340	AZOBR_150202	AZCBG_420117
AZOLI_1740	AZL_018350	AZOBR_150203	AZCBG_420118
AZOLI_1741	AZL_018360	AZOBR_150204	AZCBG_420119
AZOLI_1798	AZL_018380	AZOBR_150207	AZCBG_420123
AZOLI_1799	AZL_018390	AZOBR_150208	AZCBG_420125
AZOLI_1800	AZL_018400	AZOBR_150209	AZCBG_420126
AZOLI_1802	AZL_018420	AZOBR_150211	AZCBG_420128
AZOLI_1804	AZL_018440	AZOBR_150213	AZCBG_420130
AZOLI_1805	AZL_018450	AZOBR_150214	AZCBG_420131
AZOLI_1814	AZL_018520	AZOBR_p130067	AZCBG_p160443
AZOLI_1815	AZL_018530	AZOBR_p130068	AZCBG_p160444
AZOLI_1817	AZL_018540	AZOBR_100375	AZCBG_320050
AZOLI_1818	AZL_018550	AZOBR_100374	AZCBG_320049
AZOLI_1819	AZL_018560	AZOBR_100373	AZCBG_320048
AZOLI_1822	AZL_018570	AZOBR_100371	AZCBG_320046
AZOLI_1823	AZL_018590	AZOBR_140184	AZCBG_360014
AZOLI_1824	AZL_018600	AZOBR_p130171	AZCBG_p160556
AZOLI_1830	AZL_018640	AZOBR_40282	AZCBG_190005
AZOLI_1831	AZL_018660	AZOBR_150123	AZCBG_420045
AZOLI_1833	AZL_018680	AZOBR_p130136	AZCBG_p160524
AZOLI_1837	AZL_018720	AZOBR_p130141	AZCBG_p160530
AZOLI_1838	AZL_2092	AZOBR_p130142	AZCBG_p160531
AZOLI_1839	AZL_018730	AZOBR_100183	AZCBG_300053
AZOLI_1842	AZL_018740	AZOBR_100180	AZCBG_300050
AZOLI_1843	AZL_018750	AZOBR_100179	AZCBG_300049
AZOLI_1844	AZL_018760	AZOBR_100178	AZCBG_300048

AZOLI_1845	AZL_018770	AZOBR_100177	AZCBG_300047
AZOLI_1846	AZL_018780	AZOBR_100176	AZCBG_300046
AZOLI_1849	AZL_018790	AZOBR_150109	AZCBG_420033
AZOLI_1851	AZL_018800	AZOBR_150110	AZCBG_420034
AZOLI_1852	AZL_018810	AZOBR_150112	AZCBG_420035
AZOLI_1854	AZL_018830	AZOBR_150114	AZCBG_420037
AZOLI_1856	AZL_019130	AZOBR_100241	AZCBG_300106
AZOLI_1857	AZL_019140	AZOBR_100239	AZCBG_300105
AZOLI_1858	AZL_019160	AZOBR_100237	AZCBG_300104
AZOLI_1860	AZL_019170	AZOBR_100236	AZCBG_300103
AZOLI_1861	AZL_019180	AZOBR_100235	AZCBG_300102
AZOLI_1863	AZL_019190	AZOBR_100234	AZCBG_300100
AZOLI_1864	AZL_019200	AZOBR_100233	AZCBG_300099
AZOLI_1865	AZL_019210	AZOBR_100230	AZCBG_300098
AZOLI_1867	AZL_019220	AZOBR_100228	AZCBG_300096
AZOLI_1872	AZL_019250	AZOBR_p1130198	AZCBG_p150221
AZOLI_1873	AZL_019260	AZOBR_p1130197	AZCBG_p150220
AZOLI_1875	AZL_019280	AZOBR_p1130195	AZCBG_p150219
AZOLI_1876	AZL_019290	AZOBR_p1130194	AZCBG_p150218
AZOLI_1878	AZL_019310	AZOBR_100091	AZCBG_280008
AZOLI_1881	AZL_019320	AZOBR_100090	AZCBG_280007
AZOLI_1883	AZL_019340	AZOBR_100088	AZCBG_280005
AZOLI_1884	AZL_019350	AZOBR_100087	AZCBG_280004
AZOLI_1885	AZL_019360	AZOBR_100085	AZCBG_280003
AZOLI_1886	AZL_019370	AZOBR_100084	AZCBG_280002
AZOLI_1889	AZL_019380	AZOBR_140068	AZCBG_350055
AZOLI_1891	AZL_019390	AZOBR_140067	AZCBG_350054
AZOLI_1892	AZL_019400	AZOBR_140065	AZCBG_350052
AZOLI_1893	AZL_019410	AZOBR_140062	AZCBG_350050
AZOLI_1895	AZL_019430	AZOBR_p1130021	AZCBG_p150056
AZOLI_1897	AZL_019440	AZOBR_p150009	AZCBG_p150055
AZOLI_1898	AZL_019450	AZOBR_p150010	AZCBG_p150054
AZOLI_1900	AZL_019470	AZOBR_p130184	AZCBG_p160568
AZOLI_1901	AZL_019480	AZOBR_p130183	AZCBG_p160567
AZOLI_1902	AZL_019490	AZOBR_p130182	AZCBG_p160566
AZOLI_1905	AZL_019510	AZOBR_p130117	AZCBG_660004
AZOLI_1907	AZL_019530	AZOBR_p130121	AZCBG_p350027
AZOLI_1909	AZL_019580	AZOBR_180116	AZCBG_490031
AZOLI_1910	AZL_019590	AZOBR_180117	AZCBG_490032
AZOLI_1912	AZL_019600	AZOBR_180118	AZCBG_490033
AZOLI_1914	AZL_019620	AZOBR_180120	AZCBG_490035
AZOLI_1915	AZL_019630	AZOBR_180121	AZCBG_490036
AZOLI_1916	AZL_019640	AZOBR_180122	AZCBG_490037
AZOLI_1917	AZL_019650	AZOBR_180123	AZCBG_490038
AZOLI_1918	AZL_019660	AZOBR_180126	AZCBG_490040
AZOLI_1919	AZL_019670	AZOBR_180127	AZCBG_490041
AZOLI_1921	AZL_019680	AZOBR_180128	AZCBG_490043
AZOLI_1922	AZL_019690	AZOBR_180129	AZCBG_490044
AZOLI_1926	AZL_019710	AZOBR_180131	AZCBG_490047
AZOLI_1927	AZL_019720	AZOBR_180133	AZCBG_490048
AZOLI_1931	AZL_019750	AZOBR_70002	AZCBG_220074
AZOLI_1944	AZL_019790	AZOBR_p220002	AZCBG_p280016
AZOLI_1946	AZL_019800	AZOBR_40415	AZCBG_220010
AZOLI_1951	AZL_019850	AZOBR_40409	AZCBG_220005
AZOLI_1952	AZL_019860	AZOBR_40408	AZCBG_220004
AZOLI_1955	AZL_019880	AZOBR_40407	AZCBG_220002
AZOLI_1957	AZL_019900	AZOBR_40406	AZCBG_220001
AZOLI_1958	AZL_019920	AZOBR_40404	AZCBG_210017
AZOLI_1960	AZL_019930	AZOBR_p440153	AZCBG_p4110017

AZOLI_1962	AZL_019980	AZOBR_p440026	AZCBG_p480018
AZOLI_1964	AZL_019990	AZOBR_p440025	AZCBG_p480017
AZOLI_1965	AZL_020000	AZOBR_p440024	AZCBG_p480016
AZOLI_1966	AZL_020010	AZOBR_p440023	AZCBG_p480015
AZOLI_1967	AZL_020020	AZOBR_p440022	AZCBG_p480014
AZOLI_1969	AZL_020030	AZOBR_180024	AZCBG_480047
AZOLI_1970	AZL_020040	AZOBR_180025	AZCBG_480048
AZOLI_1973	AZL_020060	AZOBR_70183	AZCBG_260003
AZOLI_1974	AZL_020070	AZOBR_70184	AZCBG_260004
AZOLI_1975	AZL_020080	AZOBR_70185	AZCBG_260005
AZOLI_1976	AZL_020090	AZOBR_70186	AZCBG_260006
AZOLI_2108	AZL_020630	AZOBR_p120124	AZCBG_p160373
AZOLI_2109	AZL_020640	AZOBR_50032	AZCBG_p360076
AZOLI_2110	AZL_020650	AZOBR_180083	AZCBG_490001
AZOLI_2115	AZL_020690	AZOBR_180086	AZCBG_490004
AZOLI_2116	AZL_020700	AZOBR_180087	AZCBG_490005
AZOLI_2117	AZL_020710	AZOBR_180088	AZCBG_490006
AZOLI_2121	AZL_020730	AZOBR_180090	AZCBG_490007
AZOLI_2122	AZL_020740	AZOBR_180091	AZCBG_490008
AZOLI_2124	AZL_020760	AZOBR_180093	AZCBG_490010
AZOLI_2125	AZL_020770	AZOBR_180094	AZCBG_490011
AZOLI_2126	AZL_020780	AZOBR_180095	AZCBG_490012
AZOLI_2127	AZL_020790	AZOBR_180096	AZCBG_490013
AZOLI_2128	AZL_020800	AZOBR_180097	AZCBG_490014
AZOLI_2129	AZL_020810	AZOBR_180098	AZCBG_490015
AZOLI_2132	AZL_020830	AZOBR_180103	AZCBG_490018
AZOLI_2133	AZL_020840	AZOBR_180104	AZCBG_490019
AZOLI_2134	AZL_020850	AZOBR_180105	AZCBG_490020
AZOLI_2135	AZL_020860	AZOBR_180106	AZCBG_490021
AZOLI_2136	AZL_020870	AZOBR_180107	AZCBG_490022
AZOLI_2140	AZL_020890	AZOBR_180110	AZCBG_490024
AZOLI_2143	AZL_020910	AZOBR_180112	AZCBG_490027
AZOLI_2144	AZL_020920	AZOBR_180113	AZCBG_490028
AZOLI_2145	AZL_020930	AZOBR_p130122	AZCBG_p350026
AZOLI_2146	AZL_020940	AZOBR_p130123	AZCBG_p350025
AZOLI_2147	AZL_020950	AZOBR_p130124	AZCBG_p350024
AZOLI_2149	AZL_020960	AZOBR_100050	AZCBG_270068
AZOLI_2150	AZL_020970	AZOBR_100049	AZCBG_270067
AZOLI_2151	AZL_020980	AZOBR_100048	AZCBG_270066
AZOLI_2153	AZL_021000	AZOBR_100045	AZCBG_270064
AZOLI_2155	AZL_021010	AZOBR_100040	AZCBG_270058
AZOLI_2156	AZL_021020	AZOBR_100041	AZCBG_270059
AZOLI_2157	AZL_021030	AZOBR_p1160011	AZCBG_p160012
AZOLI_2161	AZL_021060	AZOBR_150144	AZCBG_420062
AZOLI_2162	AZL_021070	AZOBR_150145	AZCBG_420063
AZOLI_2163	AZL_021080	AZOBR_150146	AZCBG_420064
AZOLI_2165	AZL_021090	AZOBR_100201	AZCBG_300069
AZOLI_2167	AZL_021110	AZOBR_100199	AZCBG_300067
AZOLI_2168	AZL_021120	AZOBR_100198	AZCBG_300066
AZOLI_2169	AZL_021130	AZOBR_100195	AZCBG_300064
AZOLI_2171	AZL_021150	AZOBR_100191	AZCBG_300060
AZOLI_2172	AZL_021160	AZOBR_100190	AZCBG_300059
AZOLI_2173	AZL_021170	AZOBR_100187	AZCBG_300056
AZOLI_2174	AZL_021180	AZOBR_100186	AZCBG_300055
AZOLI_2176	AZL_021200	AZOBR_100268	AZCBG_310003
AZOLI_2177	AZL_021210	AZOBR_100267	AZCBG_310002
AZOLI_2178	AZL_021220	AZOBR_100266	AZCBG_310001
AZOLI_2180	AZL_021250	AZOBR_100265	AZCBG_300129
AZOLI_2186	AZL_021290	AZOBR_100262	AZCBG_300126

AZOLI_2188	AZL_021310	AZOBR_100260	AZCBG_300124
AZOLI_2190	AZL_021320	AZOBR_100259	AZCBG_300123
AZOLI_2192	AZL_021340	AZOBR_100257	AZCBG_300121
AZOLI_2193	AZL_021350	AZOBR_100256	AZCBG_300120
AZOLI_2194	AZL_021360	AZOBR_100255	AZCBG_300119
AZOLI_2195	AZL_021370	AZOBR_100254	AZCBG_300118
AZOLI_2196	AZL_021380	AZOBR_100253	AZCBG_300117
AZOLI_2197	AZL_021390	AZOBR_100252	AZCBG_300116
AZOLI_2198	AZL_021400	AZOBR_100251	AZCBG_300115
AZOLI_2199	AZL_021410	AZOBR_100250	AZCBG_300114
AZOLI_2200	AZL_021420	AZOBR_100249	AZCBG_300113
AZOLI_2201	AZL_021430	AZOBR_100248	AZCBG_300112
AZOLI_2202	AZL_021440	AZOBR_100247	AZCBG_300111
AZOLI_2203	AZL_021450	AZOBR_100246	AZCBG_300110
AZOLI_2204	AZL_021460	AZOBR_100245	AZCBG_300109
AZOLI_2205	AZL_021470	AZOBR_100244	AZCBG_300108
AZOLI_2206	AZL_021480	AZOBR_100243	AZCBG_300107
AZOLI_2212	AZL_021510	AZOBR_140186	AZCBG_360016
AZOLI_2213	AZL_021520	AZOBR_140185	AZCBG_360015
AZOLI_2214	AZL_021530	AZOBR_100059	AZCBG_270075
AZOLI_2217	AZL_021560	AZOBR_10355	AZCBG_70074
AZOLI_2218	AZL_021570	AZOBR_10354	AZCBG_70073
AZOLI_2219	AZL_021580	AZOBR_100055	AZCBG_270072
AZOLI_2224	AZL_021620	AZOBR_100113	AZCBG_290017
AZOLI_2226	AZL_021630	AZOBR_100114	AZCBG_290018
AZOLI_2228	AZL_021640	AZOBR_100116	AZCBG_290019
AZOLI_2229	AZL_021650	AZOBR_100117	AZCBG_290020
AZOLI_2230	AZL_021660	AZOBR_100118	AZCBG_290021
AZOLI_2231	AZL_021670	AZOBR_100119	AZCBG_290022
AZOLI_2232	AZL_021700	AZOBR_100112	AZCBG_290016
AZOLI_2236	AZL_021730	AZOBR_100111	AZCBG_290015
AZOLI_2237	AZL_021740	AZOBR_100110	AZCBG_290014
AZOLI_2238	AZL_021750	AZOBR_100109	AZCBG_290013
AZOLI_2239	AZL_021760	AZOBR_100108	AZCBG_290012
AZOLI_2240	AZL_021770	AZOBR_100106	AZCBG_290010
AZOLI_2242	AZL_021790	AZOBR_110041	AZCBG_330039
AZOLI_2246	AZL_021820	AZOBR_110037	AZCBG_330036
AZOLI_2247	AZL_021830	AZOBR_110036	AZCBG_330035
AZOLI_2249	AZL_021850	AZOBR_110034	AZCBG_330033
AZOLI_2250	AZL_021860	AZOBR_110033	AZCBG_330032
AZOLI_2251	AZL_021870	AZOBR_110032	AZCBG_330031
AZOLI_2252	AZL_021880	AZOBR_110031	AZCBG_330030
AZOLI_2253	AZL_021890	AZOBR_110030	AZCBG_330029
AZOLI_2254	AZL_021900	AZOBR_110027	AZCBG_330026
AZOLI_2256	AZL_021910	AZOBR_110026	AZCBG_330024
AZOLI_2257	AZL_021920	AZOBR_110025	AZCBG_330023
AZOLI_2260	AZL_021950	AZOBR_140031	AZCBG_350021
AZOLI_2262	AZL_021970	AZOBR_140030	AZCBG_350020
AZOLI_2265	AZL_021990	AZOBR_140024	AZCBG_350015
AZOLI_2269	AZL_022200	AZOBR_140021	AZCBG_350012
AZOLI_2270	AZL_022210	AZOBR_140020	AZCBG_350010
AZOLI_2271	AZL_022220	AZOBR_150090	AZCBG_420014
AZOLI_2272	AZL_2508	AZOBR_150091	AZCBG_420015
AZOLI_2273	AZL_022230	AZOBR_p130133	AZCBG_p160521
AZOLI_2274	AZL_022240	AZOBR_p130134	AZCBG_p160522
AZOLI_2275	AZL_022250	AZOBR_p130135	AZCBG_p160523
AZOLI_2280	AZL_022270	AZOBR_140116	AZCBG_350099
AZOLI_2282	AZL_022290	AZOBR_180061	AZCBG_230020
AZOLI_2284	AZL_022300	AZOBR_180063	AZCBG_230019

AZOLI_2285	AZL_022310	AZOBR_p440146	AZCBG_p4110011
AZOLI_2286	AZL_022320	AZOBR_p440147	AZCBG_p4110012
AZOLI_2287	AZL_022330	AZOBR_p440148	AZCBG_p4110013
AZOLI_2288	AZL_022340	AZOBR_p440149	AZCBG_p4110014
AZOLI_2290	AZL_022350	AZOBR_p440152	AZCBG_p4110016
AZOLI_2294	AZL_022380	AZOBR_180074	AZCBG_230008
AZOLI_2303	AZL_022430	AZOBR_70156	AZCBG_240018
AZOLI_2304	AZL_022440	AZOBR_70155	AZCBG_240017
AZOLI_2309	AZL_022460	AZOBR_70148	AZCBG_240012
AZOLI_2311	AZL_022470	AZOBR_70147	AZCBG_240011
AZOLI_2312	AZL_022480	AZOBR_70146	AZCBG_240010
AZOLI_2313	AZL_022490	AZOBR_70145	AZCBG_240009
AZOLI_2314	AZL_022500	AZOBR_70144	AZCBG_240008
AZOLI_2315	AZL_022510	AZOBR_70143	AZCBG_240007
AZOLI_2316	AZL_022520	AZOBR_70142	AZCBG_240006
AZOLI_2319	AZL_022550	AZOBR_70139	AZCBG_240003
AZOLI_2321	AZL_022570	AZOBR_70137	AZCBG_240001
AZOLI_2323	AZL_022590	AZOBR_200192	AZCBG_10023
AZOLI_2327	AZL_022630	AZOBR_200196	AZCBG_10027
AZOLI_2342	AZL_2567	AZOBR_180066	AZCBG_230016
AZOLI_2343	AZL_022760	AZOBR_180065	AZCBG_230017
AZOLI_2344	AZL_022770	AZOBR_180064	AZCBG_230018
AZOLI_2345	AZL_022890	AZOBR_180012	AZCBG_480037
AZOLI_2348	AZL_022870	AZOBR_40379	AZCBG_200009
AZOLI_2349	AZL_022860	AZOBR_40378	AZCBG_200008
AZOLI_2351	AZL_022810	AZOBR_200214	AZCBG_10044
AZOLI_2352	AZL_022800	AZOBR_200213	AZCBG_10043
AZOLI_2355	AZL_022900	AZOBR_180011	AZCBG_480036
AZOLI_2357	AZL_022910	AZOBR_40357	AZCBG_190069
AZOLI_2358	AZL_022920	AZOBR_40356	AZCBG_190068
AZOLI_2362	AZL_022940	AZOBR_40352	AZCBG_190066
AZOLI_2363	AZL_2589	AZOBR_40350	AZCBG_190064
AZOLI_2366	AZL_022960	AZOBR_40347	AZCBG_190061
AZOLI_2367	AZL_022970	AZOBR_40346	AZCBG_190060
AZOLI_2368	AZL_022980	AZOBR_40345	AZCBG_190059
AZOLI_2369	AZL_022990	AZOBR_40344	AZCBG_190058
AZOLI_2370	AZL_023000	AZOBR_40343	AZCBG_190057
AZOLI_2372	AZL_023020	AZOBR_40340	AZCBG_190055
AZOLI_2375	AZL_023040	AZOBR_10025	AZCBG_20008
AZOLI_2376	AZL_023050	AZOBR_10024	AZCBG_20007
AZOLI_2380	AZL_023060	AZOBR_10023	AZCBG_20006
AZOLI_2381	AZL_023070	AZOBR_10022	AZCBG_20005
AZOLI_2382	AZL_023080	AZOBR_10020	AZCBG_20004
AZOLI_2383	AZL_023090	AZOBR_10019	AZCBG_20003
AZOLI_2384	AZL_023100	AZOBR_10018	AZCBG_20002
AZOLI_2385	AZL_023110	AZOBR_180009	AZCBG_480034
AZOLI_2388	AZL_023130	AZOBR_p440018	AZCBG_p480011
AZOLI_2389	AZL_023140	AZOBR_p440016	AZCBG_p480010
AZOLI_2391	AZL_023160	AZOBR_p440014	AZCBG_p480007
AZOLI_2393	AZL_023170	AZOBR_p440012	AZCBG_p480005
AZOLI_2395	AZL_023190	AZOBR_70159	AZCBG_240022
AZOLI_2396	AZL_023200	AZOBR_70158	AZCBG_240021
AZOLI_2398	AZL_023210	AZOBR_70157	AZCBG_240020
AZOLI_2402	AZL_023230	AZOBR_150224	AZCBG_420137
AZOLI_2403	AZL_023240	AZOBR_150225	AZCBG_420138
AZOLI_2404	AZL_023250	AZOBR_150226	AZCBG_420139
AZOLI_2408	AZL_023260	AZOBR_40326	AZCBG_190044
AZOLI_2409	AZL_023270	AZOBR_40325	AZCBG_190043
AZOLI_2411	AZL_023280	AZOBR_40324	AZCBG_190042

AZOLI_2412	AZL_023290	AZOBR_40323	AZCBG_190041
AZOLI_2413	AZL_023300	AZOBR_40322	AZCBG_190040
AZOLI_2417	AZL_023340	AZOBR_40314	AZCBG_190031
AZOLI_2418	AZL_023350	AZOBR_40316	AZCBG_190032
AZOLI_2419	AZL_023360	AZOBR_40317	AZCBG_190033
AZOLI_2420	AZL_023370	AZOBR_40318	AZCBG_190034
AZOLI_2421	AZL_023380	AZOBR_40319	AZCBG_190035
AZOLI_2422	AZL_023390	AZOBR_40321	AZCBG_190037
AZOLI_2423	AZL_023400	AZOBR_40309	AZCBG_190027
AZOLI_2424	AZL_2643	AZOBR_200199	AZCBG_10030
AZOLI_2425	AZL_023410	AZOBR_200200	AZCBG_10031
AZOLI_2427	AZL_023430	AZOBR_200203	AZCBG_10033
AZOLI_2429	AZL_023450	AZOBR_200205	AZCBG_10035
AZOLI_2431	AZL_023470	AZOBR_200207	AZCBG_10037
AZOLI_2432	AZL_023480	AZOBR_200208	AZCBG_10038
AZOLI_2433	AZL_023490	AZOBR_200209	AZCBG_10039
AZOLI_2434	AZL_023500	AZOBR_200210	AZCBG_10040
AZOLI_2435	AZL_023510	AZOBR_200211	AZCBG_10041
AZOLI_2436	AZL_023520	AZOBR_200212	AZCBG_10042
AZOLI_2437	AZL_023540	AZOBR_40368	AZCBG_190079
AZOLI_2438	AZL_023560	AZOBR_p60027	AZCBG_p60031
AZOLI_2439	AZL_023570	AZOBR_40075	AZCBG_160037
AZOLI_2441	AZL_023590	AZOBR_40072	AZCBG_160034
AZOLI_2444	AZL_2665	AZOBR_40193	AZCBG_160139
AZOLI_2445	AZL_023620	AZOBR_40205	AZCBG_160150
AZOLI_2446	AZL_023630	AZOBR_40206	AZCBG_160151
AZOLI_2447	AZL_023640	AZOBR_40207	AZCBG_160152
AZOLI_2449	AZL_023650	AZOBR_40208	AZCBG_160153
AZOLI_2450	AZL_023660	AZOBR_180002	AZCBG_480027
AZOLI_2452	AZL_023680	AZOBR_170010	AZCBG_480025
AZOLI_2453	AZL_023690	AZOBR_10405	AZCBG_80038
AZOLI_2454	AZL_023700	AZOBR_170009	AZCBG_480024
AZOLI_2455	AZL_023710	AZOBR_170008	AZCBG_480023
AZOLI_2459	AZL_023750	AZOBR_70098	AZCBG_230109
AZOLI_2463	AZL_023760	AZOBR_180029	AZCBG_230049
AZOLI_2464	AZL_023770	AZOBR_180028	AZCBG_230050
AZOLI_2465	AZL_023790	AZOBR_70182	AZCBG_260002
AZOLI_2468	AZL_023810	AZOBR_70176	AZCBG_250011
AZOLI_2470	AZL_023830	AZOBR_70179	AZCBG_250013
AZOLI_2471	AZL_023840	AZOBR_70181	AZCBG_260001
AZOLI_2476	AZL_023880	AZOBR_70173	AZCBG_250008
AZOLI_2478	AZL_023910	AZOBR_70172	AZCBG_250007
AZOLI_2479	AZL_023920	AZOBR_40194	AZCBG_160140
AZOLI_2484	AZL_023980	AZOBR_200139	AZCBG_510047
AZOLI_2485	AZL_023990	AZOBR_30007	AZCBG_150004
AZOLI_2490	AZL_024030	AZOBR_40199	AZCBG_160145
AZOLI_2491	AZL_024040	AZOBR_40200	AZCBG_160146
AZOLI_2492	AZL_024050	AZOBR_40201	AZCBG_160147
AZOLI_2493	AZL_024060	AZOBR_40174	AZCBG_160122
AZOLI_2494	AZL_024070	AZOBR_40173	AZCBG_160121
AZOLI_2495	AZL_024080	AZOBR_40172	AZCBG_160120
AZOLI_2498	AZL_024220	AZOBR_40359	AZCBG_190071
AZOLI_2501	AZL_024210	AZOBR_40362	AZCBG_190074
AZOLI_2504	AZL_024190	AZOBR_200143	AZCBG_510050
AZOLI_2505	AZL_024180	AZOBR_200144	AZCBG_510051
AZOLI_2510	AZL_024230	AZOBR_10169	AZCBG_40056
AZOLI_2511	AZL_024240	AZOBR_10168	AZCBG_40055
AZOLI_2512	AZL_024250	AZOBR_40085	AZCBG_160045
AZOLI_2513	AZL_024260	AZOBR_40084	AZCBG_160044

AZOLI_2514	AZL_024270	AZOBR_40083	AZCBG_160043
AZOLI_2517	AZL_024300	AZOBR_40367	AZCBG_190078
AZOLI_2518	AZL_024310	AZOBR_40364	AZCBG_190076
AZOLI_2519	AZL_024320	AZOBR_40363	AZCBG_190075
AZOLI_2520	AZL_024330	AZOBR_40170	AZCBG_160119
AZOLI_2523	AZL_024360	AZOBR_30029	AZCBG_140010
AZOLI_2526	AZL_024380	AZOBR_10049	AZCBG_20029
AZOLI_2528	AZL_024390	AZOBR_10051	AZCBG_20031
AZOLI_2530	AZL_024410	AZOBR_40063	AZCBG_160026
AZOLI_2531	AZL_024420	AZOBR_40064	AZCBG_160027
AZOLI_2533	AZL_024440	AZOBR_40067	AZCBG_160030
AZOLI_2537	AZL_024460	AZOBR_10076	AZCBG_30008
AZOLI_2538	AZL_024470	AZOBR_10075	AZCBG_30007
AZOLI_2554	AZL_024570	AZOBR_p410011	AZCBG_p420008
AZOLI_2564	AZL_024650	AZOBR_p470021	AZCBG_p4200005
AZOLI_2574	AZL_024730	AZOBR_200034	AZCBG_490211
AZOLI_2575	AZL_024740	AZOBR_200035	AZCBG_490212
AZOLI_2579	AZL_024760	AZOBR_200038	AZCBG_490214
AZOLI_2580	AZL_024770	AZOBR_200039	AZCBG_490215
AZOLI_2583	AZL_2796	AZOBR_200041	AZCBG_490216
AZOLI_2585	AZL_024790	AZOBR_200044	AZCBG_490219
AZOLI_2586	AZL_024800	AZOBR_200045	AZCBG_490220
AZOLI_2588	AZL_024820	AZOBR_200047	AZCBG_490222
AZOLI_2589	AZL_024830	AZOBR_200048	AZCBG_490223
AZOLI_2590	AZL_024840	AZOBR_200049	AZCBG_490224
AZOLI_2592	AZL_024850	AZOBR_200119	AZCBG_490225
AZOLI_2594	AZL_2806	AZOBR_200222	AZCBG_10052
AZOLI_2595	AZL_024860	AZOBR_200223	AZCBG_10053
AZOLI_2600	AZL_024900	AZOBR_40308	AZCBG_190026
AZOLI_2601	AZL_024910	AZOBR_40307	AZCBG_190025
AZOLI_2602	AZL_024920	AZOBR_40306	AZCBG_190024
AZOLI_2603	AZL_024930	AZOBR_40305	AZCBG_190023
AZOLI_2604	AZL_024940	AZOBR_40304	AZCBG_190022
AZOLI_2605	AZL_024950	AZOBR_40303	AZCBG_190021
AZOLI_2610	AZL_024970	AZOBR_40300	AZCBG_190018
AZOLI_2611	AZL_024980	AZOBR_40299	AZCBG_190017
AZOLI_2612	AZL_024990	AZOBR_40298	AZCBG_190016
AZOLI_2613	AZL_025000	AZOBR_40297	AZCBG_190015
AZOLI_2619	AZL_025020	AZOBR_40292	AZCBG_190014
AZOLI_2623	AZL_025050	AZOBR_40288	AZCBG_190010
AZOLI_2624	AZL_a09260	AZOBR_40287	AZCBG_190009
AZOLI_2625	AZL_025060	AZOBR_40285	AZCBG_190008
AZOLI_2628	AZL_025080	AZOBR_40280	AZCBG_190004
AZOLI_2629	AZL_025090	AZOBR_40279	AZCBG_190003
AZOLI_2630	AZL_025100	AZOBR_40277	AZCBG_190001
AZOLI_2633	AZL_025130	AZOBR_40274	AZCBG_180006
AZOLI_2635	AZL_025140	AZOBR_40275	AZCBG_180007
AZOLI_2637	AZL_025150	AZOBR_180195	AZCBG_490090
AZOLI_2638	AZL_2839	AZOBR_180196	AZCBG_490091
AZOLI_2639	AZL_025160	AZOBR_180197	AZCBG_490092
AZOLI_2641	AZL_003890	AZOBR_40130	AZCBG_160085
AZOLI_2647	AZL_003840	AZOBR_p1110031	AZCBG_p130033
AZOLI_2648	AZL_003830	AZOBR_p1110030	AZCBG_p130032
AZOLI_2650	AZL_003810	AZOBR_10215	AZCBG_40095
AZOLI_2652	AZL_003780	AZOBR_10218	AZCBG_40098
AZOLI_2654	AZL_003760	AZOBR_10221	AZCBG_40100
AZOLI_2655	AZL_003750	AZOBR_10222	AZCBG_40101
AZOLI_2658	AZL_003720	AZOBR_10240	AZCBG_40120
AZOLI_2659	AZL_003710	AZOBR_10239	AZCBG_40119

AZOLI_2660	AZL_003700	AZOBR_10238	AZCBG_40118
AZOLI_2661	AZL_003690	AZOBR_10237	AZCBG_40117
AZOLI_2663	AZL_003680	AZOBR_10236	AZCBG_40115
AZOLI_2664	AZL_003670	AZOBR_10235	AZCBG_40114
AZOLI_2668	AZL_003630	AZOBR_180258	AZCBG_490144
AZOLI_2670	AZL_003610	AZOBR_30009	AZCBG_150002
AZOLI_2671	AZL_003600	AZOBR_30010	AZCBG_150001
AZOLI_2672	AZL_003590	AZOBR_30011	AZCBG_140028
AZOLI_2675	AZL_003560	AZOBR_30001	AZCBG_150011
AZOLI_2676	AZL_003550	AZOBR_30002	AZCBG_150010
AZOLI_2682	AZL_003480	AZOBR_30032	AZCBG_140007
AZOLI_2685	AZL_003460	AZOBR_20016	AZCBG_120004
AZOLI_2686	AZL_003450	AZOBR_20015	AZCBG_120003
AZOLI_2690	AZL_003440	AZOBR_20012	AZCBG_120001
AZOLI_2691	AZL_003430	AZOBR_20008	AZCBG_110030
AZOLI_2694	AZL_003400	AZOBR_10253	AZCBG_60006
AZOLI_2695	AZL_003390	AZOBR_10254	AZCBG_60007
AZOLI_2697	AZL_003380	AZOBR_10256	AZCBG_60009
AZOLI_2698	AZL_003370	AZOBR_10257	AZCBG_60010
AZOLI_2699	AZL_003360	AZOBR_10258	AZCBG_60011
AZOLI_2700	AZL_003350	AZOBR_10259	AZCBG_60012
AZOLI_2702	AZL_0374	AZOBR_10261	AZCBG_60013
AZOLI_2703	AZL_003340	AZOBR_10262	AZCBG_60014
AZOLI_2705	AZL_003330	AZOBR_10263	AZCBG_60015
AZOLI_2706	AZL_003320	AZOBR_10266	AZCBG_60017
AZOLI_2707	AZL_0370	AZOBR_10267	AZCBG_60018
AZOLI_2708	AZL_003310	AZOBR_10269	AZCBG_60019
AZOLI_2709	AZL_003300	AZOBR_10270	AZCBG_60020
AZOLI_2710	AZL_003190	AZOBR_40209	AZCBG_160155
AZOLI_2711	AZL_003200	AZOBR_40210	AZCBG_160156
AZOLI_2715	AZL_003230	AZOBR_40103	AZCBG_160063
AZOLI_2716	AZL_003240	AZOBR_40102	AZCBG_160062
AZOLI_2717	AZL_003250	AZOBR_40101	AZCBG_160061
AZOLI_2718	AZL_003260	AZOBR_40100	AZCBG_160060
AZOLI_2719	AZL_003270	AZOBR_p1110072	AZCBG_p130068
AZOLI_2721	AZL_003290	AZOBR_180037	AZCBG_230041
AZOLI_2724	AZL_002540	AZOBR_40243	AZCBG_160183
AZOLI_2728	AZL_002520	AZOBR_40240	AZCBG_160181
AZOLI_2730	AZL_002500	AZOBR_p1110068	AZCBG_p130065
AZOLI_2732	AZL_002490	AZOBR_40234	AZCBG_160176
AZOLI_2736	AZL_002460	AZOBR_10210	AZCBG_40091
AZOLI_2740	AZL_002430	AZOBR_10204	AZCBG_40085
AZOLI_2742	AZL_002420	AZOBR_10202	AZCBG_40084
AZOLI_2743	AZL_002410	AZOBR_10201	AZCBG_40078
AZOLI_2744	AZL_002400	AZOBR_10198	AZCBG_40079
AZOLI_2745	AZL_002390	AZOBR_10040	AZCBG_20019
AZOLI_2746	AZL_002380	AZOBR_10041	AZCBG_20021
AZOLI_2747	AZL_002370	AZOBR_10043	AZCBG_20022
AZOLI_2749	AZL_002340	AZOBR_40226	AZCBG_160169
AZOLI_2751	AZL_002330	AZOBR_40223	AZCBG_160167
AZOLI_2754	AZL_002160	AZOBR_p1110064	AZCBG_p130062
AZOLI_2757	AZL_002190	AZOBR_10293	AZCBG_70019
AZOLI_2761	AZL_002110	AZOBR_10163	AZCBG_40050
AZOLI_2765	AZL_001920	AZOBR_p280024	AZCBG_p210034
AZOLI_2768	AZL_001940	AZOBR_p280144	AZCBG_730010
AZOLI_2770	AZL_001960	AZOBR_10158	AZCBG_40044
AZOLI_2772	AZL_001970	AZOBR_40096	AZCBG_160056
AZOLI_2774	AZL_001990	AZOBR_40094	AZCBG_160054
AZOLI_2775	AZL_002000	AZOBR_10061	AZCBG_20041

AZOLI_2777	AZL_002020	AZOBR_10063	AZCBG_20043
AZOLI_2784	AZL_002050	AZOBR_40061	AZCBG_160024
AZOLI_2785	AZL_002060	AZOBR_180260	AZCBG_490146
AZOLI_2787	AZL_002080	AZOBR_180261	AZCBG_490147
AZOLI_2788	AZL_002090	AZOBR_180263	AZCBG_490149
AZOLI_2793	AZL_001910	AZOBR_180265	AZCBG_490155
AZOLI_2794	AZL_001900	AZOBR_180266	AZCBG_490156
AZOLI_2795	AZL_001890	AZOBR_20019	AZCBG_120007
AZOLI_2796	AZL_001880	AZOBR_180267	AZCBG_490157
AZOLI_2797	AZL_001870	AZOBR_180269	AZCBG_490158
AZOLI_2798	AZL_001860	AZOBR_180270	AZCBG_490159
AZOLI_2799	AZL_001850	AZOBR_200121	AZCBG_490227
AZOLI_2802	AZL_001830	AZOBR_200123	AZCBG_490229
AZOLI_2804	AZL_001810	AZOBR_200126	AZCBG_510035
AZOLI_2805	AZL_001800	AZOBR_200127	AZCBG_510036
AZOLI_2807	AZL_001790	AZOBR_200128	AZCBG_510037
AZOLI_2809	AZL_001770	AZOBR_200131	AZCBG_510040
AZOLI_2811	AZL_001760	AZOBR_200133	AZCBG_510042
AZOLI_2813	AZL_001740	AZOBR_200136	AZCBG_510044
AZOLI_2814	AZL_001730	AZOBR_200137	AZCBG_510045
AZOLI_2817	AZL_0189	AZOBR_200138	AZCBG_510046
AZOLI_2819	AZL_001710	AZOBR_10166	AZCBG_40053
AZOLI_2820	AZL_001700	AZOBR_10167	AZCBG_40054
AZOLI_2821	AZL_001690	AZOBR_p1170037	AZCBG_p160095
AZOLI_2823	AZL_001670	AZOBR_p130119	AZCBG_660002
AZOLI_2824	AZL_001660	AZOBR_40233	AZCBG_160175
AZOLI_2826	AZL_001640	AZOBR_40232	AZCBG_160174
AZOLI_2827	AZL_001630	AZOBR_40216	AZCBG_160161
AZOLI_2832	AZL_001600	AZOBR_40048	AZCBG_160010
AZOLI_2833	AZL_001590	AZOBR_40049	AZCBG_160011
AZOLI_2834	AZL_0173	AZOBR_40050	AZCBG_160012
AZOLI_2835	AZL_001580	AZOBR_40051	AZCBG_160013
AZOLI_2836	AZL_001570	AZOBR_40052	AZCBG_160014
AZOLI_2837	AZL_001560	AZOBR_40053	AZCBG_160015
AZOLI_2839	AZL_001540	AZOBR_40059	AZCBG_160022
AZOLI_2842	AZL_001520	AZOBR_40057	AZCBG_160019
AZOLI_2844	AZL_001500	AZOBR_40054	AZCBG_160017
AZOLI_2845	AZL_001490	AZOBR_10172	AZCBG_40057
AZOLI_2847	AZL_001480	AZOBR_10174	AZCBG_40059
AZOLI_2848	AZL_001470	AZOBR_10175	AZCBG_40060
AZOLI_2849	AZL_b03720	AZOBR_40214	AZCBG_160159
AZOLI_2850	AZL_001460	AZOBR_p1110063	AZCBG_p130061
AZOLI_2852	AZL_001450	AZOBR_p1110061	AZCBG_p130060
AZOLI_2855	AZL_001420	AZOBR_p1110058	AZCBG_p130056
AZOLI_2857	AZL_001410	AZOBR_p1110056	AZCBG_p130054
AZOLI_2858	AZL_001400	AZOBR_p1110055	AZCBG_p130053
AZOLI_2859	AZL_001390	AZOBR_p1110054	AZCBG_p130052
AZOLI_2861	AZL_001160	AZOBR_p1110051	AZCBG_p130050
AZOLI_2862	AZL_001150	AZOBR_p1110050	AZCBG_p130049
AZOLI_2863	AZL_001140	AZOBR_p1110049	AZCBG_p130048
AZOLI_2864	AZL_001130	AZOBR_p1110048	AZCBG_p130047
AZOLI_2870	AZL_001110	AZOBR_p1110047	AZCBG_p130046
AZOLI_2871	AZL_001100	AZOBR_p1110046	AZCBG_p130045
AZOLI_2872	AZL_001090	AZOBR_p1110045	AZCBG_p130044
AZOLI_2873	AZL_001080	AZOBR_p1110044	AZCBG_p130043
AZOLI_2874	AZL_001070	AZOBR_p1110043	AZCBG_p130042
AZOLI_2876	AZL_001050	AZOBR_p1110041	AZCBG_p130040
AZOLI_2877	AZL_001040	AZOBR_p1110040	AZCBG_p130039
AZOLI_2881	AZL_001030	AZOBR_p1110039	AZCBG_p130038

AZOLI_2883	AZL_001020	AZOBR_p1110038	AZCBG_p130037
AZOLI_2886	AZL_001010	AZOBR_p1110037	AZCBG_p130036
AZOLI_2893	AZL_000980	AZOBR_40251	AZCBG_160189
AZOLI_2894	AZL_000960	AZOBR_40252	AZCBG_160190
AZOLI_2896	AZL_000950	AZOBR_40253	AZCBG_160192
AZOLI_2898	AZL_000930	AZOBR_10127	AZCBG_40034
AZOLI_2899	AZL_000920	AZOBR_10128	AZCBG_40033
AZOLI_2900	AZL_000910	AZOBR_10129	AZCBG_40032
AZOLI_2901	AZL_000900	AZOBR_10130	AZCBG_40031
AZOLI_2902	AZL_000890	AZOBR_10131	AZCBG_40030
AZOLI_2904	AZL_026840	AZOBR_10288	AZCBG_70013
AZOLI_2905	AZL_026850	AZOBR_10289	AZCBG_70014
AZOLI_2908	AZL_026880	AZOBR_10290	AZCBG_70016
AZOLI_2909	AZL_026900	AZOBR_10291	AZCBG_70017
AZOLI_2918	AZL_026910	AZOBR_10132	AZCBG_40029
AZOLI_2919	AZL_026920	AZOBR_10133	AZCBG_40028
AZOLI_2920	AZL_026930	AZOBR_10134	AZCBG_40027
AZOLI_2921	AZL_026940	AZOBR_10135	AZCBG_40026
AZOLI_2922	AZL_026950	AZOBR_10136	AZCBG_40025
AZOLI_2924	AZL_027040	AZOBR_10138	AZCBG_40022
AZOLI_2925	AZL_027050	AZOBR_10102	AZCBG_30080
AZOLI_2926	AZL_027060	AZOBR_10103	AZCBG_30081
AZOLI_2929	AZL_027070	AZOBR_10104	AZCBG_30082
AZOLI_2930	AZL_027080	AZOBR_10105	AZCBG_30083
AZOLI_2932	AZL_027100	AZOBR_10229	AZCBG_40110
AZOLI_2933	AZL_027110	AZOBR_10231	AZCBG_40111
AZOLI_2934	AZL_027120	AZOBR_10233	AZCBG_40112
AZOLI_2935	AZL_027130	AZOBR_10234	AZCBG_40113
AZOLI_2939	AZL_000880	AZOBR_20024	AZCBG_130005
AZOLI_2940	AZL_000870	AZOBR_40126	AZCBG_160080
AZOLI_2943	AZL_000840	AZOBR_40121	AZCBG_160077
AZOLI_2944	AZL_000830	AZOBR_40120	AZCBG_160076
AZOLI_2945	AZL_000820	AZOBR_p120085	AZCBG_p160340
AZOLI_2946	AZL_000810	AZOBR_40119	AZCBG_160075
AZOLI_2947	AZL_000800	AZOBR_40118	AZCBG_160074
AZOLI_2949	AZL_000780	AZOBR_40116	AZCBG_160072
AZOLI_2950	AZL_000770	AZOBR_40114	AZCBG_160071
AZOLI_2951	AZL_000760	AZOBR_40113	AZCBG_160070
AZOLI_2954	AZL_000740	AZOBR_10197	AZCBG_40080
AZOLI_2958	AZL_000710	AZOBR_p430050	AZCBG_p2210015
AZOLI_2959	AZL_000700	AZOBR_p430051	AZCBG_p2210014
AZOLI_2960	AZL_000690	AZOBR_p430053	AZCBG_p2210013
AZOLI_2961	AZL_000680	AZOBR_p430054	AZCBG_p2210012
AZOLI_2965	AZL_000650	AZOBR_p430057	AZCBG_p2210009
AZOLI_2966	AZL_000640	AZOBR_p430058	AZCBG_p2210008
AZOLI_2969	AZL_000620	AZOBR_p430062	AZCBG_p2210006
AZOLI_2980	AZL_000500	AZOBR_10330	AZCBG_70050
AZOLI_2982	AZL_000490	AZOBR_10331	AZCBG_70051
AZOLI_2983	AZL_000480	AZOBR_10333	AZCBG_70052
AZOLI_2984	AZL_000470	AZOBR_10334	AZCBG_70053
AZOLI_2986	AZL_000450	AZOBR_10336	AZCBG_70055
AZOLI_2987	AZL_000440	AZOBR_10337	AZCBG_70056
AZOLI_2988	AZL_000430	AZOBR_10491	AZCBG_100062
AZOLI_2989	AZL_000420	AZOBR_10492	AZCBG_100063
AZOLI_2990	AZL_000410	AZOBR_10493	AZCBG_100064
AZOLI_2991	AZL_000400	AZOBR_10494	AZCBG_100065
AZOLI_2992	AZL_000390	AZOBR_10495	AZCBG_100066
AZOLI_2995	AZL_000360	AZOBR_10501	AZCBG_100072
AZOLI_2997	AZL_000350	AZOBR_10500	AZCBG_100071

AZOLI_2998	AZL_000340	AZOBR_10499	AZCBG_100070
AZOLI_2999	AZL_000330	AZOBR_10498	AZCBG_100069
AZOLI_3000	AZL_000320	AZOBR_10497	AZCBG_100068
AZOLI_3001	AZL_000310	AZOBR_10496	AZCBG_100067
AZOLI_3002	AZL_000300	AZOBR_100221	AZCBG_300089
AZOLI_3003	AZL_000290	AZOBR_10223	AZCBG_40102
AZOLI_3004	AZL_000280	AZOBR_10241	AZCBG_50001
AZOLI_3005	AZL_000270	AZOBR_10243	AZCBG_50003
AZOLI_3006	AZL_000260	AZOBR_10244	AZCBG_50004
AZOLI_3007	AZL_000250	AZOBR_10245	AZCBG_50005
AZOLI_3008	AZL_000240	AZOBR_10246	AZCBG_60001
AZOLI_3014	AZL_000200	AZOBR_10249	AZCBG_60003
AZOLI_3015	AZL_000190	AZOBR_10486	AZCBG_100056
AZOLI_3016	AZL_000180	AZOBR_10485	AZCBG_100055
AZOLI_3017	AZL_000170	AZOBR_10484	AZCBG_100054
AZOLI_3018	AZL_000160	AZOBR_10482	AZCBG_100052
AZOLI_3019	AZL_000150	AZOBR_10404	AZCBG_80037
AZOLI_3020	AZL_000140	AZOBR_10403	AZCBG_80036
AZOLI_3021	AZL_000130	AZOBR_10402	AZCBG_80035
AZOLI_3022	AZL_000120	AZOBR_10401	AZCBG_80034
AZOLI_3023	AZL_000110	AZOBR_10400	AZCBG_80033
AZOLI_3025	AZL_000090	AZOBR_10398	AZCBG_80031
AZOLI_3026	AZL_000080	AZOBR_10397	AZCBG_80030
AZOLI_3027	AZL_000070	AZOBR_10396	AZCBG_80029
AZOLI_3028	AZL_000060	AZOBR_10395	AZCBG_80028
AZOLI_3029	AZL_000050	AZOBR_10394	AZCBG_80027
AZOLI_3030	AZL_000040	AZOBR_10393	AZCBG_80026
AZOLI_3031	AZL_000030	AZOBR_10392	AZCBG_80025
AZOLI_3032	AZL_000020	AZOBR_10391	AZCBG_80024
AZOLI_3033	AZL_000010	AZOBR_10390	AZCBG_80023
AZOLI_3035	AZL_028930	AZOBR_10388	AZCBG_80022
AZOLI_3037	AZL_028910	AZOBR_10387	AZCBG_80021
AZOLI_3038	AZL_028900	AZOBR_10382	AZCBG_80017
AZOLI_3039	AZL_028890	AZOBR_10381	AZCBG_80016
AZOLI_3042	AZL_028870	AZOBR_10378	AZCBG_80013
AZOLI_3043	AZL_028860	AZOBR_10377	AZCBG_80012
AZOLI_3044	AZL_028850	AZOBR_10366	AZCBG_80001
AZOLI_3045	AZL_028840	AZOBR_10367	AZCBG_80002
AZOLI_3048	AZL_028820	AZOBR_10369	AZCBG_80004
AZOLI_3049	AZL_028810	AZOBR_10370	AZCBG_80005
AZOLI_3050	AZL_028800	AZOBR_10371	AZCBG_80006
AZOLI_3051	AZL_028790	AZOBR_10372	AZCBG_80007
AZOLI_3052	AZL_028780	AZOBR_10373	AZCBG_80008
AZOLI_3053	AZL_028770	AZOBR_10374	AZCBG_80009
AZOLI_3054	AZL_028760	AZOBR_10375	AZCBG_80010
AZOLI_3056	AZL_028740	AZOBR_10361	AZCBG_70080
AZOLI_3057	AZL_028720	AZOBR_10157	AZCBG_40043
AZOLI_3059	AZL_028700	AZOBR_10155	AZCBG_40041
AZOLI_3060	AZL_028690	AZOBR_10154	AZCBG_40040
AZOLI_3061	AZL_028680	AZOBR_10153	AZCBG_40039
AZOLI_3063	AZL_028660	AZOBR_10151	AZCBG_40037
AZOLI_3065	AZL_028650	AZOBR_40196	AZCBG_160142
AZOLI_3068	AZL_028630	AZOBR_10306	AZCBG_70030
AZOLI_3070	AZL_028620	AZOBR_10308	AZCBG_70033
AZOLI_3077	AZL_028570	AZOBR_10313	AZCBG_70038
AZOLI_3078	AZL_028560	AZOBR_10314	AZCBG_70039
AZOLI_3079	AZL_028550	AZOBR_10315	AZCBG_70040
AZOLI_3080	AZL_028540	AZOBR_10319	AZCBG_70041
AZOLI_3081	AZL_028530	AZOBR_10320	AZCBG_70042

AZOLI_3083	AZL_028510	AZOBR_10323	AZCBG_70044
AZOLI_3084	AZL_028500	AZOBR_10324	AZCBG_70045
AZOLI_3086	AZL_028480	AZOBR_10326	AZCBG_70047
AZOLI_3087	AZL_028470	AZOBR_10327	AZCBG_70048
AZOLI_3089	AZL_028450	AZOBR_10089	AZCBG_30068
AZOLI_3091	AZL_028440	AZOBR_10090	AZCBG_30069
AZOLI_3093	AZL_028420	AZOBR_10526	AZCBG_110012
AZOLI_3098	AZL_028370	AZOBR_10347	AZCBG_70066
AZOLI_3100	AZL_028350	AZOBR_10345	AZCBG_70064
AZOLI_3101	AZL_028340	AZOBR_10344	AZCBG_70063
AZOLI_3104	AZL_028310	AZOBR_10343	AZCBG_70062
AZOLI_3105	AZL_028300	AZOBR_10342	AZCBG_70061
AZOLI_3106	AZL_028290	AZOBR_10341	AZCBG_70060
AZOLI_3107	AZL_028280	AZOBR_10338	AZCBG_70057
AZOLI_3108	AZL_028270	AZOBR_10490	AZCBG_100061
AZOLI_3109	AZL_028260	AZOBR_10489	AZCBG_100059
AZOLI_3110	AZL_028250	AZOBR_10488	AZCBG_100058
AZOLI_3111	AZL_028240	AZOBR_10487	AZCBG_100057
AZOLI_3112	AZL_028230	AZOBR_10509	AZCBG_670004
AZOLI_3114	AZL_028180	AZOBR_10448	AZCBG_100016
AZOLI_3118	AZL_028140	AZOBR_10454	AZCBG_100024
AZOLI_3119	AZL_028130	AZOBR_10457	AZCBG_100027
AZOLI_3120	AZL_028120	AZOBR_10458	AZCBG_100028
AZOLI_3121	AZL_028090	AZOBR_10479	AZCBG_100048
AZOLI_3123	AZL_028070	AZOBR_10477	AZCBG_100046
AZOLI_3124	AZL_028060	AZOBR_10476	AZCBG_100045
AZOLI_3125	AZL_028050	AZOBR_10475	AZCBG_100044
AZOLI_3126	AZL_028040	AZOBR_10139	AZCBG_40021
AZOLI_3127	AZL_028030	AZOBR_10140	AZCBG_40020
AZOLI_3129	AZL_028010	AZOBR_10142	AZCBG_40018
AZOLI_3131	AZL_028000	AZOBR_10143	AZCBG_40017
AZOLI_3133	AZL_027990	AZOBR_10146	AZCBG_40014
AZOLI_3134	AZL_027980	AZOBR_10147	AZCBG_40013
AZOLI_3135	AZL_027970	AZOBR_p130080	AZCBG_p160455
AZOLI_3137	AZL_027960	AZOBR_p430063	AZCBG_p2210005
AZOLI_3153	AZL_027280	AZOBR_p170041	AZCBG_p1100043
AZOLI_3154	AZL_027270	AZOBR_p170040	AZCBG_p1100042
AZOLI_3155	AZL_027260	AZOBR_p170039	AZCBG_p1100041
AZOLI_3157	AZL_027240	AZOBR_p170038	AZCBG_p1100040
AZOLI_3159	AZL_027220	AZOBR_10124	AZCBG_40012
AZOLI_3164	AZL_027190	AZOBR_10118	AZCBG_40008
AZOLI_3165	AZL_027180	AZOBR_10117	AZCBG_40007
AZOLI_3167	AZL_3081	AZOBR_10115	AZCBG_40005
AZOLI_3168	AZL_027160	AZOBR_10114	AZCBG_40004
AZOLI_3169	AZL_026830	AZOBR_10510	AZCBG_670003
AZOLI_3171	AZL_026810	AZOBR_10513	AZCBG_670001
AZOLI_3174	AZL_026790	AZOBR_10514	AZCBG_110001
AZOLI_3175	AZL_026740	AZOBR_10194	AZCBG_40083
AZOLI_3176	AZL_026730	AZOBR_10282	AZCBG_70008
AZOLI_p10013	AZL_a07620	AZOBR_140311	AZCBG_370106
AZOLI_p10015	AZL_a07630	AZOBR_140310	AZCBG_370105
AZOLI_p10018	AZL_a07660	AZOBR_110088	AZCBG_330084
AZOLI_p10019	AZL_a07670	AZOBR_110085	AZCBG_330081
AZOLI_p10020	AZL_a07680	AZOBR_p1130013	AZCBG_p140101
AZOLI_p10023	AZL_b05730	AZOBR_p1170094	AZCBG_p160149
AZOLI_p10024	AZL_a02290	AZOBR_p210005	AZCBG_730007
AZOLI_p10025	AZL_a02280	AZOBR_p210006	AZCBG_730006
AZOLI_p10027	AZL_a02260	AZOBR_p210008	AZCBG_730004
AZOLI_p10028	AZL_a02250	AZOBR_p210009	AZCBG_730003

AZOLI_p10029	AZL_a02240	AZOBR_p330145	AZCBG_730002
AZOLI_p10030	AZL_a02230	AZOBR_p210012	AZCBG_730001
AZOLI_p10045	AZL_a11280	AZOBR_10032	AZCBG_20013
AZOLI_p10052	AZL_a00020	AZOBR_p480065	AZCBG_p4230084
AZOLI_p10053	AZL_a00030	AZOBR_p480055	AZCBG_p410006
AZOLI_p10066	AZL_a00110	AZOBR_p210112	AZCBG_p2200035
AZOLI_p10067	AZL_a00120	AZOBR_p110136	AZCBG_p160305
AZOLI_p10068	AZL_a00150	AZOBR_p110137	AZCBG_p160306
AZOLI_p10069	AZL_a00160	AZOBR_p110138	AZCBG_p160307
AZOLI_p10070	AZL_a00170	AZOBR_p110139	AZCBG_p160308
AZOLI_p10071	AZL_a00180	AZOBR_p110140	AZCBG_p160309
AZOLI_p10072	AZL_a00190	AZOBR_p110141	AZCBG_p160310
AZOLI_p10075	AZL_a00220	AZOBR_160057	AZCBG_460004
AZOLI_p10076	AZL_a00230	AZOBR_160056	AZCBG_460003
AZOLI_p10077	AZL_a00240	AZOBR_160055	AZCBG_460002
AZOLI_p10078	AZL_a00250	AZOBR_160054	AZCBG_460001
AZOLI_p10079	AZL_a00260	AZOBR_160053	AZCBG_450022
AZOLI_p10092	AZL_a00300	AZOBR_100377	AZCBG_320051
AZOLI_p10093	AZL_a00310	AZOBR_100378	AZCBG_320053
AZOLI_p10094	AZL_a00320	AZOBR_100379	AZCBG_320054
AZOLI_p10095	AZL_a00330	AZOBR_100380	AZCBG_320055
AZOLI_p10096	AZL_a00340	AZOBR_100381	AZCBG_320056
AZOLI_p10097	AZL_a00350	AZOBR_100382	AZCBG_320057
AZOLI_p10099	AZL_a00370	AZOBR_p210069	AZCBG_p230007
AZOLI_p10101	AZL_a00390	AZOBR_140106	AZCBG_350092
AZOLI_p10102	AZL_a00400	AZOBR_140107	AZCBG_350093
AZOLI_p10105	AZL_a00410	AZOBR_140110	AZCBG_350094
AZOLI_p10106	AZL_a00420	AZOBR_140111	AZCBG_350095
AZOLI_p10107	AZL_a00430	AZOBR_140112	AZCBG_350096
AZOLI_p10187	AZL_a02320	AZOBR_p110094	AZCBG_p160271
AZOLI_p10190	AZL_a02480	AZOBR_p210028	AZCBG_p220013
AZOLI_p10191	AZL_a02490	AZOBR_p210023	AZCBG_p30003
AZOLI_p10193	AZL_a02500	AZOBR_p210022	AZCBG_p30002
AZOLI_p10194	AZL_a02510	AZOBR_p210021	AZCBG_p30001
AZOLI_p10195	AZL_a02520	AZOBR_p210020	AZCBG_640006
AZOLI_p10196	AZL_a02530	AZOBR_p210019	AZCBG_640005
AZOLI_p10197	AZL_a02540	AZOBR_p210018	AZCBG_640004
AZOLI_p10198	AZL_a02550	AZOBR_p210017	AZCBG_640003
AZOLI_p10199	AZL_a02560	AZOBR_p210016	AZCBG_640002
AZOLI_p10200	AZL_a02570	AZOBR_p110049	AZCBG_p160232
AZOLI_p10251	AZL_009290	AZOBR_p1130026	AZCBG_p150061
AZOLI_p10253	AZL_a08020	AZOBR_p470098	AZCBG_p4230045
AZOLI_p10256	AZL_a02580	AZOBR_p220102	AZCBG_p2110012
AZOLI_p10259	AZL_a02610	AZOBR_150077	AZCBG_420002
AZOLI_p10260	AZL_a02630	AZOBR_100383	AZCBG_320058
AZOLI_p10262	AZL_a02650	AZOBR_100336	AZCBG_320014
AZOLI_p10263	AZL_a02660	AZOBR_100335	AZCBG_320013
AZOLI_p10265	AZL_a02670	AZOBR_100333	AZCBG_320011
AZOLI_p10269	AZL_a02710	AZOBR_100341	AZCBG_320018
AZOLI_p10271	AZL_a02730	AZOBR_100344	AZCBG_320020
AZOLI_p10272	AZL_a02740	AZOBR_100345	AZCBG_320021
AZOLI_p10273	AZL_a02750	AZOBR_100347	AZCBG_320024
AZOLI_p10276	AZL_a02770	AZOBR_p1130199	AZCBG_p150222
AZOLI_p10287	AZL_a03270	AZOBR_110004	AZCBG_330006
AZOLI_p10290	AZL_a03290	AZOBR_110011	AZCBG_330012
AZOLI_p10293	AZL_a03320	AZOBR_110015	AZCBG_330015
AZOLI_p10294	AZL_a03330	AZOBR_100404	AZCBG_320098
AZOLI_p10295	AZL_a03340	AZOBR_110016	AZCBG_330016
AZOLI_p10297	AZL_a03350	AZOBR_100401	AZCBG_320096

AZOLI_p10298	AZL_a03360	AZOBR_100405	AZCBG_320099
AZOLI_p10299	AZL_a03370	AZOBR_100402	AZCBG_320097
AZOLI_p10302	AZL_a03390	AZOBR_100407	AZCBG_320101
AZOLI_p10306	AZL_a03430	AZOBR_p1100099	AZCBG_p120018
AZOLI_p10307	AZL_a03440	AZOBR_200193	AZCBG_10024
AZOLI_p10309	AZL_a03450	AZOBR_p1100098	AZCBG_p120017
AZOLI_p10310	AZL_a03460	AZOBR_110008	AZCBG_330010
AZOLI_p10313	AZL_a03480	AZOBR_p1100096	AZCBG_p120015
AZOLI_p10322	AZL_a03560	AZOBR_p470030	AZCBG_p4200011
AZOLI_p10325	AZL_a03580	AZOBR_p1160053	AZCBG_p160051
AZOLI_p10326	AZL_a03590	AZOBR_p1160054	AZCBG_p160052
AZOLI_p10327	AZL_a03600	AZOBR_p1160055	AZCBG_p160053
AZOLI_p10331	AZL_a03610	AZOBR_p1170004	AZCBG_p160059
AZOLI_p10332	AZL_a03620	AZOBR_p1170005	AZCBG_p160060
AZOLI_p10333	AZL_a03630	AZOBR_p1170006	AZCBG_p160061
AZOLI_p10335	AZL_a03640	AZOBR_p1170008	AZCBG_p160063
AZOLI_p10337	AZL_a03650	AZOBR_p1170010	AZCBG_p160065
AZOLI_p10338	AZL_a03660	AZOBR_p1170013	AZCBG_p160068
AZOLI_p10340	AZL_a03680	AZOBR_p1170023	AZCBG_p160082
AZOLI_p10342	AZL_a03690	AZOBR_p1170024	AZCBG_p160083
AZOLI_p10343	AZL_a03700	AZOBR_p1170025	AZCBG_p160084
AZOLI_p10344	AZL_a03710	AZOBR_p1170026	AZCBG_p160085
AZOLI_p10345	AZL_a03720	AZOBR_p1170027	AZCBG_p160086
AZOLI_p10347	AZL_a03740	AZOBR_p1170034	AZCBG_p160092
AZOLI_p10348	AZL_a03750	AZOBR_p1170035	AZCBG_p160093
AZOLI_p10349	AZL_a03770	AZOBR_180068	AZCBG_230014
AZOLI_p10350	AZL_a03780	AZOBR_180069	AZCBG_230013
AZOLI_p10352	AZL_a03800	AZOBR_180071	AZCBG_230011
AZOLI_p10354	AZL_a03810	AZOBR_p1160006	AZCBG_p160007
AZOLI_p10355	AZL_a03830	AZOBR_p140067	AZCBG_p180040
AZOLI_p10366	AZL_a03890	AZOBR_p1130160	AZCBG_p150187
AZOLI_p10367	AZL_a03900	AZOBR_p1130161	AZCBG_p150188
AZOLI_p10368	AZL_a03910	AZOBR_p1130162	AZCBG_p150189
AZOLI_p10369	AZL_a03920	AZOBR_p1130163	AZCBG_p150190
AZOLI_p10372	AZL_a03930	AZOBR_p470020	AZCBG_p4200004
AZOLI_p10373	AZL_a03940	AZOBR_p470019	AZCBG_p4200003
AZOLI_p10374	AZL_a03950	AZOBR_p470018	AZCBG_p4200002
AZOLI_p10375	AZL_a03960	AZOBR_p470017	AZCBG_p4200001
AZOLI_p10376	AZL_a03970	AZOBR_p470016	AZCBG_p4190011
AZOLI_p10393	AZL_a07870	AZOBR_p140002	AZCBG_p170061
AZOLI_p10394	AZL_a07860	AZOBR_p140003	AZCBG_p170062
AZOLI_p10395	AZL_a07850	AZOBR_p1180008	AZCBG_p160156
AZOLI_p10413	AZL_a07270	AZOBR_140158	AZCBG_350138
AZOLI_p10414	AZL_a07260	AZOBR_140159	AZCBG_350139
AZOLI_p10477	AZL_a03240	AZOBR_140162	AZCBG_350142
AZOLI_p10480	AZL_a03250	AZOBR_140166	AZCBG_350146
AZOLI_p10482	AZL_a04070	AZOBR_140169	AZCBG_350149
AZOLI_p10483	AZL_a04080	AZOBR_140170	AZCBG_350150
AZOLI_p10484	AZL_a04090	AZOBR_140171	AZCBG_360001
AZOLI_p10487	AZL_a04110	AZOBR_140172	AZCBG_360002
AZOLI_p10489	AZL_a04130	AZOBR_140174	AZCBG_360004
AZOLI_p10491	AZL_a04150	AZOBR_100130	AZCBG_300006
AZOLI_p10492	AZL_a04160	AZOBR_100129	AZCBG_300005
AZOLI_p10493	AZL_a04170	AZOBR_100128	AZCBG_300004
AZOLI_p10494	AZL_a04180	AZOBR_100127	AZCBG_300003
AZOLI_p10495	AZL_a04190	AZOBR_100126	AZCBG_300002
AZOLI_p10496	AZL_a04200	AZOBR_140175	AZCBG_360005
AZOLI_p10498	AZL_a04220	AZOBR_100136	AZCBG_300011
AZOLI_p10500	AZL_a04240	AZOBR_100138	AZCBG_300013

AZOLI_p10501	AZL_a04250	AZOBR_100139	AZCBG_300014
AZOLI_p10521	AZL_b02320	AZOBR_140097	AZCBG_350084
AZOLI_p10522	AZL_b02310	AZOBR_140098	AZCBG_350085
AZOLI_p10527	AZL_a09040	AZOBR_p420013	AZCBG_p460017
AZOLI_p10528	AZL_a09030	AZOBR_p440169	AZCBG_p4120006
AZOLI_p10532	AZL_a07070	AZOBR_p1110133	AZCBG_p130134
AZOLI_p10533	AZL_a07060	AZOBR_p1110134	AZCBG_p130135
AZOLI_p10534	AZL_a07050	AZOBR_p1110135	AZCBG_p130136
AZOLI_p10535	AZL_a07040	AZOBR_p1110136	AZCBG_p130137
AZOLI_p10549	AZL_a07010	AZOBR_p1110150	AZCBG_p140002
AZOLI_p10550	AZL_a07000	AZOBR_p1110151	AZCBG_p140003
AZOLI_p10551	AZL_a06990	AZOBR_p1110152	AZCBG_p140004
AZOLI_p10553	AZL_a06970	AZOBR_p1110155	AZCBG_p140006
AZOLI_p10554	AZL_a06960	AZOBR_p1110156	AZCBG_p140007
AZOLI_p10555	AZL_a06950	AZOBR_p1110157	AZCBG_p140008
AZOLI_p10557	AZL_a06930	AZOBR_p1110159	AZCBG_p140010
AZOLI_p10558	AZL_a06920	AZOBR_p1110160	AZCBG_p140011
AZOLI_p10559	AZL_a06910	AZOBR_p1110163	AZCBG_p140013
AZOLI_p10561	AZL_a06890	AZOBR_p1110164	AZCBG_p140014
AZOLI_p10562	AZL_a06880	AZOBR_p1110165	AZCBG_p140015
AZOLI_p10563	AZL_a06690	AZOBR_p1110166	AZCBG_p140016
AZOLI_p10564	AZL_a06680	AZOBR_p1110167	AZCBG_p140017
AZOLI_p10566	AZL_a06660	AZOBR_p1110170	AZCBG_p140020
AZOLI_p10567	AZL_a06650	AZOBR_p1120003	AZCBG_p140022
AZOLI_p10570	AZL_pA0738	AZOBR_p1120005	AZCBG_p140024
AZOLI_p10572	AZL_a06630	AZOBR_p1120008	AZCBG_p140027
AZOLI_p10573	AZL_a06620	AZOBR_p1120009	AZCBG_p140028
AZOLI_p10575	AZL_a06610	AZOBR_p1160036	AZCBG_p160035
AZOLI_p10576	AZL_a06600	AZOBR_p1160037	AZCBG_p160036
AZOLI_p10577	AZL_a06590	AZOBR_p1160038	AZCBG_p160037
AZOLI_p10578	AZL_a06580	AZOBR_p1160039	AZCBG_p160038
AZOLI_p10579	AZL_a06570	AZOBR_p1160040	AZCBG_p160039
AZOLI_p10581	AZL_a06550	AZOBR_p1160042	AZCBG_p160041
AZOLI_p10583	AZL_a06530	AZOBR_p1160044	AZCBG_p160043
AZOLI_p10585	AZL_a06510	AZOBR_p1160046	AZCBG_p160045
AZOLI_p10591	AZL_a06460	AZOBR_p1160052	AZCBG_p160050
AZOLI_p10599	AZL_a06400	AZOBR_p110050	AZCBG_p160233
AZOLI_p10600	AZL_a05490	AZOBR_p140057	AZCBG_p180030
AZOLI_p10601	AZL_a05500	AZOBR_p140056	AZCBG_p180029
AZOLI_p10605	AZL_a05530	AZOBR_p140051	AZCBG_p180025
AZOLI_p10606	AZL_a05540	AZOBR_p140050	AZCBG_p180024
AZOLI_p10607	AZL_a05550	AZOBR_p140049	AZCBG_p180023
AZOLI_p10611	AZL_a05560	AZOBR_p140022	AZCBG_p170079
AZOLI_p10619	AZL_a05570	AZOBR_p130034	AZCBG_p160409
AZOLI_p10621	AZL_a05590	AZOBR_p140020	AZCBG_p170077
AZOLI_p10624	AZL_a05610	AZOBR_p140016	AZCBG_p170073
AZOLI_p10625	AZL_a05620	AZOBR_p140015	AZCBG_p170072
AZOLI_p10628	AZL_a05630	AZOBR_p1140109	AZCBG_p150234
AZOLI_p10629	AZL_a05640	AZOBR_p1140110	AZCBG_p150235
AZOLI_p10630	AZL_a05650	AZOBR_p1140111	AZCBG_p150236
AZOLI_p10633	AZL_a05660	AZOBR_p1140112	AZCBG_p150237
AZOLI_p10636	AZL_a05690	AZOBR_p1160033	AZCBG_p160033
AZOLI_p10637	AZL_a05700	AZOBR_p1160028	AZCBG_p160027
AZOLI_p10640	AZL_a05720	AZOBR_p1130006	AZCBG_p140093
AZOLI_p10641	AZL_a05730	AZOBR_p1130007	AZCBG_p140094
AZOLI_p10643	AZL_a05290	AZOBR_p140066	AZCBG_p180039
AZOLI_p10645	AZL_a05310	AZOBR_p140063	AZCBG_p180037
AZOLI_p10646	AZL_a05320	AZOBR_p140062	AZCBG_p180036
AZOLI_p10647	AZL_a05330	AZOBR_p140061	AZCBG_p180035

AZOLI_p10649	AZL_a05350	AZOBR_p140092	AZCBG_p190012
AZOLI_p10652	AZL_a05380	AZOBR_p1130047	AZCBG_p150084
AZOLI_p10654	AZL_a05400	AZOBR_p1130050	AZCBG_p150086
AZOLI_p10655	AZL_a05410	AZOBR_p110045	AZCBG_p160228
AZOLI_p10663	AZL_a05420	AZOBR_p110036	AZCBG_p160218
AZOLI_p10668	AZL_c01630	AZOBR_p110027	AZCBG_p160210
AZOLI_p10680	AZL_c02500	AZOBR_p280056	AZCBG_p210005
AZOLI_p10736	AZL_c02910	AZOBR_p460052	AZCBG_p4170032
AZOLI_p10738	AZL_c02930	AZOBR_p460054	AZCBG_p4170034
AZOLI_p10751	AZL_a04600	AZOBR_p1100059	AZCBG_p1130002
AZOLI_p10753	AZL_a04580	AZOBR_150169	AZCBG_420086
AZOLI_p10762	AZL_a04560	AZOBR_150171	AZCBG_420088
AZOLI_p10763	AZL_a04550	AZOBR_p120107	AZCBG_p160359
AZOLI_p10766	AZL_a04520	AZOBR_p120111	AZCBG_p160363
AZOLI_p10767	AZL_a04510	AZOBR_p120112	AZCBG_p160364
AZOLI_p10768	AZL_a04500	AZOBR_p120113	AZCBG_p160365
AZOLI_p10771	AZL_a04470	AZOBR_p120117	AZCBG_p160368
AZOLI_p10772	AZL_a04460	AZOBR_p120119	AZCBG_p160369
AZOLI_p10773	AZL_a04450	AZOBR_p120120	AZCBG_p160370
AZOLI_p10774	AZL_a04440	AZOBR_p120121	AZCBG_p160371
AZOLI_p10776	AZL_a04430	AZOBR_p120122	AZCBG_p160372
AZOLI_p10806	AZL_a04390	AZOBR_p110117	AZCBG_p160288
AZOLI_p10807	AZL_a04380	AZOBR_p110118	AZCBG_p160289
AZOLI_p10809	AZL_pA0481	AZOBR_p110120	AZCBG_p160291
AZOLI_p10810	AZL_a04360	AZOBR_p110121	AZCBG_p160292
AZOLI_p10812	AZL_pA0478	AZOBR_p110124	AZCBG_p160294
AZOLI_p10814	AZL_a04330	AZOBR_p1180011	AZCBG_p160159
AZOLI_p10815	AZL_a04320	AZOBR_p1180010	AZCBG_p160158
AZOLI_p10818	AZL_a04300	AZOBR_p1180007	AZCBG_p160155
AZOLI_p10820	AZL_a04290	AZOBR_p1180006	AZCBG_p160154
AZOLI_p10849	AZL_a07220	AZOBR_p210065	AZCBG_p230003
AZOLI_p10850	AZL_a07230	AZOBR_p210064	AZCBG_p230002
AZOLI_p10852	AZL_a07240	AZOBR_p210063	AZCBG_p230001
AZOLI_p10867	AZL_c01750	AZOBR_p220082	AZCBG_p2100009
AZOLI_p10877	AZL_c01830	AZOBR_p220084	AZCBG_p2100011
AZOLI_p10901	AZL_c01890	AZOBR_p270100	AZCBG_p2180033
AZOLI_p11028	AZL_a06750	AZOBR_40339	AZCBG_190054
AZOLI_p11029	AZL_a06760	AZOBR_40338	AZCBG_190053
AZOLI_p11031	AZL_a06780	AZOBR_40329	AZCBG_190047
AZOLI_p11032	AZL_a06790	AZOBR_40328	AZCBG_190046
AZOLI_p20005	AZL_a08110	AZOBR_100411	AZCBG_620003
AZOLI_p20172	AZL_e02620	AZOBR_60020	AZCBG_p170041
AZOLI_p20231	AZL_a08540	AZOBR_p460017	AZCBG_p4170001
AZOLI_p20258	AZL_e03320	AZOBR_p210128	AZCBG_p250004
AZOLI_p20261	AZL_a04910	AZOBR_p210124	AZCBG_p2140010
AZOLI_p20286	AZL_a08320	AZOBR_p110017	AZCBG_p160200
AZOLI_p20296	AZL_a08230	AZOBR_p170138	AZCBG_p1100056
AZOLI_p20317	AZL_c01160	AZOBR_p210089	AZCBG_p240013
AZOLI_p20318	AZL_c01170	AZOBR_p210090	AZCBG_p240014
AZOLI_p20320	AZL_c01190	AZOBR_p1100018	AZCBG_p1110029
AZOLI_p20322	AZL_c01210	AZOBR_p1100017	AZCBG_p1110027
AZOLI_p20323	AZL_c01220	AZOBR_p1100016	AZCBG_p1110026
AZOLI_p20324	AZL_c01230	AZOBR_p1100015	AZCBG_p1110025
AZOLI_p20325	AZL_c01240	AZOBR_p1100014	AZCBG_p1110024
AZOLI_p20327	AZL_a02850	AZOBR_p1130086	AZCBG_p150116
AZOLI_p20329	AZL_a02870	AZOBR_p1130088	AZCBG_p150118
AZOLI_p20330	AZL_a02880	AZOBR_p1130089	AZCBG_p150119
AZOLI_p20331	AZL_a02890	AZOBR_p1130090	AZCBG_p150120
AZOLI_p20333	AZL_a02910	AZOBR_p1130092	AZCBG_p150122

AZOLI_p20334	AZL_a02920	AZOBR_p1130093	AZCBG_p150123
AZOLI_p20335	AZL_a02930	AZOBR_p1130094	AZCBG_p150124
AZOLI_p20338	AZL_a02960	AZOBR_p140099	AZCBG_p190023
AZOLI_p20342	AZL_a03000	AZOBR_p140093	AZCBG_p190018
AZOLI_p20357	AZL_a03080	AZOBR_140081	AZCBG_350071
AZOLI_p20361	AZL_a03090	AZOBR_p1100040	AZCBG_p1110049
AZOLI_p20363	AZL_a03110	AZOBR_p1100037	AZCBG_p1110047
AZOLI_p20370	AZL_a03170	AZOBR_100226	AZCBG_300094
AZOLI_p20382	AZL_a07210	AZOBR_p210029	AZCBG_p220014
AZOLI_p20384	AZL_a07190	AZOBR_p210032	AZCBG_p220016
AZOLI_p20385	AZL_a07180	AZOBR_p210036	AZCBG_p220017
AZOLI_p20393	AZL_a07120	AZOBR_p210044	AZCBG_p220023
AZOLI_p20394	AZL_a07110	AZOBR_p210045	AZCBG_p220024
AZOLI_p20395	AZL_a07100	AZOBR_p210046	AZCBG_p220025
AZOLI_p20397	AZL_a07090	AZOBR_p210048	AZCBG_p220027
AZOLI_p20398	AZL_a07080	AZOBR_p210049	AZCBG_p220028
AZOLI_p20403	AZL_a05200	AZOBR_180219	AZCBG_490112
AZOLI_p20404	AZL_a05210	AZOBR_180218	AZCBG_490111
AZOLI_p20405	AZL_a05220	AZOBR_180217	AZCBG_490110
AZOLI_p20407	AZL_a05240	AZOBR_180213	AZCBG_490107
AZOLI_p20408	AZL_a05250	AZOBR_180212	AZCBG_490106
AZOLI_p20414	AZL_a05750	AZOBR_180206	AZCBG_490100
AZOLI_p20415	AZL_a05760	AZOBR_180207	AZCBG_490101
AZOLI_p20416	AZL_a05770	AZOBR_180208	AZCBG_490102
AZOLI_p20423	AZL_a05820	AZOBR_p440074	AZCBG_p490016
AZOLI_p20424	AZL_a05830	AZOBR_p440075	AZCBG_p490017
AZOLI_p20454	AZL_c03620	AZOBR_p230061	AZCBG_p2110088
AZOLI_p20455	AZL_c03610	AZOBR_p230060	AZCBG_p2110087
AZOLI_p20457	AZL_b03100	AZOBR_p420011	AZCBG_p460019
AZOLI_p20460	AZL_b03080	AZOBR_p420025	AZCBG_p460003
AZOLI_p20499	AZL_a01340	AZOBR_p410010	AZCBG_p420007
AZOLI_p20514	AZL_b02730	AZOBR_p230078	AZCBG_p2120015
AZOLI_p20515	AZL_b02720	AZOBR_p230079	AZCBG_p2120016
AZOLI_p20516	AZL_b02710	AZOBR_p230080	AZCBG_p2120017
AZOLI_p20517	AZL_b02700	AZOBR_p230081	AZCBG_p2120018
AZOLI_p20518	AZL_b02690	AZOBR_p230082	AZCBG_p2120019
AZOLI_p20521	AZL_b04890	AZOBR_p1120037	AZCBG_p140053
AZOLI_p20522	AZL_b04880	AZOBR_p1120036	AZCBG_p140052
AZOLI_p20527	AZL_b04860	AZOBR_p440091	AZCBG_p490028
AZOLI_p20540	AZL_b04660	AZOBR_p1160024	AZCBG_p160023
AZOLI_p20542	AZL_b04640	AZOBR_p210047	AZCBG_p220026
AZOLI_p20546	AZL_a00760	AZOBR_p130099	AZCBG_p160469
AZOLI_p20554	AZL_b04580	AZOBR_p170036	AZCBG_p1100037
AZOLI_p20564	AZL_b05120	AZOBR_p130049	AZCBG_p160424
AZOLI_p20565	AZL_b05110	AZOBR_p130048	AZCBG_p160423
AZOLI_p20566	AZL_b05100	AZOBR_p130047	AZCBG_p160422
AZOLI_p20567	AZL_b05090	AZOBR_p130045	AZCBG_p160421
AZOLI_p20568	AZL_b05080	AZOBR_p130044	AZCBG_p160420
AZOLI_p20574	AZL_b05020	AZOBR_160046	AZCBG_450015
AZOLI_p20575	AZL_b05010	AZOBR_p280005	AZCBG_p210050
AZOLI_p20594	AZL_b04930	AZOBR_p170026	AZCBG_p1100028
AZOLI_p20628	AZL_b02910	AZOBR_p480063	AZCBG_p4230086
AZOLI_p20629	AZL_b02900	AZOBR_p220098	AZCBG_p2110008
AZOLI_p20630	AZL_b02890	AZOBR_p170019	AZCBG_p1100022
AZOLI_p20631	AZL_a10620	AZOBR_p220105	AZCBG_p2110014
AZOLI_p20691	AZL_b06300	AZOBR_p230093	AZCBG_p2120031
AZOLI_p20692	AZL_b06310	AZOBR_p230094	AZCBG_p2120032
AZOLI_p20707	AZL_b00120	AZOBR_p230087	AZCBG_p2120023
AZOLI_p20743	AZL_b00240	AZOBR_p60071	AZCBG_p60100

AZOLI_p30004	AZL_c00390	AZOBR_p1120063	AZCBG_p140079
AZOLI_p30047	AZL_b05190	AZOBR_40382	AZCBG_200012
AZOLI_p30049	AZL_a10630	AZOBR_p1120068	AZCBG_p140083
AZOLI_p30050	AZL_a10640	AZOBR_p1120069	AZCBG_p140084
AZOLI_p30051	AZL_a10650	AZOBR_p1120070	AZCBG_p140085
AZOLI_p30069	AZL_c02000	AZOBR_p1130145	AZCBG_p150170
AZOLI_p30072	AZL_c02020	AZOBR_p1130134	AZCBG_p150158
AZOLI_p30082	AZL_c02030	AZOBR_p310167	AZCBG_p340161
AZOLI_p30083	AZL_c02040	AZOBR_100170	AZCBG_300039
AZOLI_p30084	AZL_c02050	AZOBR_p350035	AZCBG_p340166
AZOLI_p30085	AZL_c02060	AZOBR_p350036	AZCBG_p340165
AZOLI_p30086	AZL_c02070	AZOBR_p350037	AZCBG_p340183
AZOLI_p30087	AZL_c02080	AZOBR_p350038	AZCBG_p340182
AZOLI_p30089	AZL_c02090	AZOBR_p410038	AZCBG_p430013
AZOLI_p30101	AZL_c02200	AZOBR_40440	AZCBG_p210053
AZOLI_p30104	AZL_c02220	AZOBR_120024	AZCBG_330138
AZOLI_p30105	AZL_c02230	AZOBR_120023	AZCBG_330137
AZOLI_p30108	AZL_c02260	AZOBR_120020	AZCBG_330134
AZOLI_p30109	AZL_c02270	AZOBR_120019	AZCBG_330133
AZOLI_p30112	AZL_c02280	AZOBR_120018	AZCBG_330132
AZOLI_p30113	AZL_c02290	AZOBR_120016	AZCBG_330131
AZOLI_p30114	AZL_c02300	AZOBR_120015	AZCBG_330130
AZOLI_p30115	AZL_c02310	AZOBR_120014	AZCBG_330129
AZOLI_p30123	AZL_c02370	AZOBR_p1170049	AZCBG_p160107
AZOLI_p30124	AZL_c02360	AZOBR_p1170050	AZCBG_p160108
AZOLI_p30125	AZL_c02350	AZOBR_p1170051	AZCBG_p160109
AZOLI_p30127	AZL_c02330	AZOBR_120012	AZCBG_330127
AZOLI_p30128	AZL_c02320	AZOBR_120013	AZCBG_330128
AZOLI_p30135	AZL_c01620	AZOBR_180019	AZCBG_480042
AZOLI_p30138	AZL_c02530	AZOBR_p460073	AZCBG_p4180015
AZOLI_p30140	AZL_c02550	AZOBR_p460072	AZCBG_p4180014
AZOLI_p30141	AZL_c02560	AZOBR_p460071	AZCBG_p4180013
AZOLI_p30143	AZL_c02580	AZOBR_p130212	AZCBG_p170054
AZOLI_p30144	AZL_c02590	AZOBR_p130213	AZCBG_p170055
AZOLI_p30145	AZL_c02600	AZOBR_p130214	AZCBG_p170056
AZOLI_p30148	AZL_a10110	AZOBR_p1140117	AZCBG_p150241
AZOLI_p30165	AZL_c01530	AZOBR_p410040	AZCBG_p430015
AZOLI_p30169	AZL_c01490	AZOBR_p220032	AZCBG_p280046
AZOLI_p30170	AZL_c01480	AZOBR_p280059	AZCBG_p210002
AZOLI_p30173	AZL_c01460	AZOBR_p410047	AZCBG_p440006
AZOLI_p30175	AZL_c01440	AZOBR_30020	AZCBG_140019
AZOLI_p30182	AZL_c01010	AZOBR_p1140024	AZCBG_p360053
AZOLI_p30183	AZL_c01000	AZOBR_p1140023	AZCBG_p360052
AZOLI_p30184	AZL_c00990	AZOBR_p1140022	AZCBG_p360050
AZOLI_p30185	AZL_c00980	AZOBR_p1140021	AZCBG_p360049
AZOLI_p30186	AZL_c00970	AZOBR_p1140020	AZCBG_p360048
AZOLI_p30187	AZL_c00960	AZOBR_p1140019	AZCBG_p360047
AZOLI_p30190	AZL_c00940	AZOBR_p1140016	AZCBG_p360045
AZOLI_p30191	AZL_c00930	AZOBR_p1140015	AZCBG_p360044
AZOLI_p30192	AZL_c00920	AZOBR_p1120074	AZCBG_p140088
AZOLI_p30193	AZL_c00910	AZOBR_p1120073	AZCBG_p140087
AZOLI_p30195	AZL_c00860	AZOBR_p150050	AZCBG_p150024
AZOLI_p30197	AZL_c00880	AZOBR_150084	AZCBG_420007
AZOLI_p30201	AZL_c02770	AZOBR_p440044	AZCBG_p480028
AZOLI_p30205	AZL_c02780	AZOBR_p440043	AZCBG_p480027
AZOLI_p30206	AZL_c02790	AZOBR_p440042	AZCBG_p480026
AZOLI_p30207	AZL_c02800	AZOBR_p440041	AZCBG_p480025
AZOLI_p30209	AZL_c02820	AZOBR_100146	AZCBG_300019
AZOLI_p30210	AZL_c02830	AZOBR_100145	AZCBG_300018

AZOLI_p30211	AZL_002350	AZOBR_p1140101	AZCBG_p150226
AZOLI_p30236	AZL_c04090	AZOBR_10242	AZCBG_50002
AZOLI_p30238	AZL_c04100	AZOBR_150080	AZCBG_420004
AZOLI_p30239	AZL_c04110	AZOBR_150079	AZCBG_420003
AZOLI_p30241	AZL_c04130	AZOBR_p1110129	AZCBG_p130130
AZOLI_p30242	AZL_c04140	AZOBR_p1110128	AZCBG_p130129
AZOLI_p30246	AZL_c04170	AZOBR_p150065	AZCBG_p150010
AZOLI_p30247	AZL_c04210	AZOBR_70096	AZCBG_230107
AZOLI_p30250	AZL_c04240	AZOBR_p110102	AZCBG_p160277
AZOLI_p30252	AZL_c04260	AZOBR_p110104	AZCBG_p160279
AZOLI_p30253	AZL_c04270	AZOBR_p110105	AZCBG_p160280
AZOLI_p30255	AZL_c04280	AZOBR_p110106	AZCBG_p160281
AZOLI_p30256	AZL_c04290	AZOBR_p110107	AZCBG_p160282
AZOLI_p30257	AZL_c04300	AZOBR_p110108	AZCBG_p160283
AZOLI_p30258	AZL_c04310	AZOBR_p110109	AZCBG_p160284
AZOLI_p30260	AZL_c04330	AZOBR_p110111	AZCBG_p160286
AZOLI_p30267	AZL_c04350	AZOBR_40086	AZCBG_160046
AZOLI_p30268	AZL_c04360	AZOBR_150158	AZCBG_420076
AZOLI_p30271	AZL_c04380	AZOBR_p210120	AZCBG_p2200029
AZOLI_p30273	AZL_c04400	AZOBR_p210117	AZCBG_p2200031
AZOLI_p30282	AZL_c04410	AZOBR_p150017	AZCBG_p150046
AZOLI_p30288	AZL_c04430	AZOBR_p420022	AZCBG_p460007
AZOLI_p30290	AZL_c04440	AZOBR_p420023	AZCBG_p460006
AZOLI_p30291	AZL_c04450	AZOBR_p420012	AZCBG_p460018
AZOLI_p30296	AZL_c04510	AZOBR_p440170	AZCBG_p4120007
AZOLI_p30313	AZL_c04610	AZOBR_p440034	AZCBG_p480022
AZOLI_p30323	AZL_c00730	AZOBR_130012	AZCBG_330189
AZOLI_p30328	AZL_c00720	AZOBR_130014	AZCBG_330190
AZOLI_p30329	AZL_c00710	AZOBR_130015	AZCBG_330191
AZOLI_p30330	AZL_c00700	AZOBR_130016	AZCBG_330192
AZOLI_p30331	AZL_c00690	AZOBR_130017	AZCBG_330193
AZOLI_p30340	AZL_c00640	AZOBR_130018	AZCBG_330194
AZOLI_p30341	AZL_c00630	AZOBR_130019	AZCBG_330195
AZOLI_p30342	AZL_c00620	AZOBR_130020	AZCBG_330196
AZOLI_p30345	AZL_c00570	AZOBR_p1130119	AZCBG_p150143
AZOLI_p30347	AZL_c00560	AZOBR_p1130120	AZCBG_p150144
AZOLI_p30352	AZL_c00520	AZOBR_p1130112	AZCBG_p150138
AZOLI_p30353	AZL_c00510	AZOBR_p1130110	AZCBG_p150137
AZOLI_p30354	AZL_c00500	AZOBR_p1130109	AZCBG_p150136
AZOLI_p30355	AZL_c00490	AZOBR_p1130107	AZCBG_p150134
AZOLI_p30356	AZL_c00480	AZOBR_p1130106	AZCBG_p150133
AZOLI_p30357	AZL_c00470	AZOBR_p1130105	AZCBG_p150132
AZOLI_p30358	AZL_c00460	AZOBR_p1130104	AZCBG_p150131
AZOLI_p30359	AZL_pC0049	AZOBR_p1130102	AZCBG_p150130
AZOLI_p30361	AZL_c00430	AZOBR_p1130097	AZCBG_p150127
AZOLI_p30364	AZL_c00410	AZOBR_p130070	AZCBG_p160446
AZOLI_p30368	AZL_c00380	AZOBR_p1120062	AZCBG_p140078
AZOLI_p30369	AZL_c00370	AZOBR_p1120061	AZCBG_p140077
AZOLI_p30370	AZL_c00360	AZOBR_p1120060	AZCBG_p140076
AZOLI_p30372	AZL_c00340	AZOBR_p1120058	AZCBG_p140074
AZOLI_p30376	AZL_c00280	AZOBR_p1120047	AZCBG_p140063
AZOLI_p30378	AZL_c00260	AZOBR_p1120049	AZCBG_p140065
AZOLI_p30379	AZL_c00240	AZOBR_p1120050	AZCBG_p140066
AZOLI_p30380	AZL_c00230	AZOBR_p1120051	AZCBG_p140067
AZOLI_p30381	AZL_c00220	AZOBR_p1120052	AZCBG_p140068
AZOLI_p30383	AZL_c00200	AZOBR_p1110126	AZCBG_p130127
AZOLI_p30385	AZL_c00180	AZOBR_p1120007	AZCBG_p140026
AZOLI_p30386	AZL_c00170	AZOBR_p440184	AZCBG_p4140005
AZOLI_p30387	AZL_c00160	AZOBR_p440183	AZCBG_p4140004

AZOLI_p30395	AZL_b03310	AZOBR_p210180	AZCBG_p260047
AZOLI_p30396	AZL_b03300	AZOBR_p210181	AZCBG_p260049
AZOLI_p30397	AZL_b03290	AZOBR_p1100048	AZCBG_p1110057
AZOLI_p30398	AZL_b03280	AZOBR_p210182	AZCBG_p260050
AZOLI_p30399	AZL_b03270	AZOBR_p210183	AZCBG_p270001
AZOLI_p30400	AZL_b03260	AZOBR_p210184	AZCBG_p270002
AZOLI_p30401	AZL_b03250	AZOBR_p210185	AZCBG_p270003
AZOLI_p30404	AZL_b03220	AZOBR_p210188	AZCBG_p270006
AZOLI_p30405	AZL_b03210	AZOBR_p210191	AZCBG_p280001
AZOLI_p30406	AZL_c00130	AZOBR_p410100	AZCBG_p450010
AZOLI_p30407	AZL_c00120	AZOBR_p280134	AZCBG_730017
AZOLI_p30429	AZL_c05180	AZOBR_p220118	AZCBG_p2110026
AZOLI_p30430	AZL_c05190	AZOBR_140216	AZCBG_370017
AZOLI_p30431	AZL_c05200	AZOBR_p230091	AZCBG_p2120029
AZOLI_p30432	AZL_c05250	AZOBR_p170147	AZCBG_p1100065
AZOLI_p30436	AZL_c05290	AZOBR_p1130004	AZCBG_p140091
AZOLI_p30437	AZL_c05300	AZOBR_10422	AZCBG_80052
AZOLI_p30447	AZL_c00030	AZOBR_p220074	AZCBG_p2100002
AZOLI_p30449	AZL_c00060	AZOBR_p220092	AZCBG_p2110003
AZOLI_p30450	AZL_c00070	AZOBR_100334	AZCBG_320012
AZOLI_p30451	AZL_c00080	AZOBR_p220091	AZCBG_p2110002
AZOLI_p30452	AZL_c00090	AZOBR_p1170014	AZCBG_p160069
AZOLI_p30455	AZL_c00110	AZOBR_p1170018	AZCBG_p160072
AZOLI_p30457	AZL_c04960	AZOBR_p230039	AZCBG_p2110067
AZOLI_p30461	AZL_c04990	AZOBR_p210122	AZCBG_p2200027
AZOLI_p30472	AZL_c05040	AZOBR_p110091	AZCBG_p160269
AZOLI_p30493	AZL_c04920	AZOBR_p1170057	AZCBG_p160114
AZOLI_p30494	AZL_c04910	AZOBR_p1170058	AZCBG_p160115
AZOLI_p30495	AZL_c04900	AZOBR_p1170059	AZCBG_p160116
AZOLI_p30496	AZL_c04880	AZOBR_p1170069	AZCBG_p160125
AZOLI_p30497	AZL_c04740	AZOBR_p1140107	AZCBG_p150232
AZOLI_p30500	AZL_c04760	AZOBR_p1140106	AZCBG_p150231
AZOLI_p30501	AZL_c04770	AZOBR_p1140104	AZCBG_p150229
AZOLI_p30502	AZL_c04780	AZOBR_p1140103	AZCBG_p150228
AZOLI_p30503	AZL_c04790	AZOBR_p1140102	AZCBG_p150227
AZOLI_p30506	AZL_c04820	AZOBR_p1140013	AZCBG_p360042
AZOLI_p30507	AZL_c04830	AZOBR_p1140012	AZCBG_p360041
AZOLI_p30508	AZL_c04840	AZOBR_p1140011	AZCBG_p360040
AZOLI_p30509	AZL_c04850	AZOBR_p1140010	AZCBG_p360039
AZOLI_p30510	AZL_c04860	AZOBR_p1140009	AZCBG_p360038
AZOLI_p30511	AZL_c04870	AZOBR_p1140008	AZCBG_p360037
AZOLI_p30512	AZL_pC0542	AZOBR_p110053	AZCBG_p160235
AZOLI_p30514	AZL_c04720	AZOBR_p280101	AZCBG_p2210057
AZOLI_p30518	AZL_c04700	AZOBR_110028	AZCBG_330027
AZOLI_p30533	AZL_c00820	AZOBR_p480080	AZCBG_p4230072
AZOLI_p30556	AZL_c02640	AZOBR_p480014	AZCBG_p410052
AZOLI_p30557	AZL_c02630	AZOBR_p480015	AZCBG_p410051
AZOLI_p30559	AZL_c02610	AZOBR_p480016	AZCBG_p410049
AZOLI_p30580	AZL_a10060	AZOBR_p130039	AZCBG_p160415
AZOLI_p30582	AZL_a10040	AZOBR_p130041	AZCBG_p160417
AZOLI_p30586	AZL_a10000	AZOBR_100320	AZCBG_320001
AZOLI_p30587	AZL_a09990	AZOBR_100321	AZCBG_320002
AZOLI_p30588	AZL_a09980	AZOBR_100322	AZCBG_320003
AZOLI_p30589	AZL_a09970	AZOBR_100323	AZCBG_320004
AZOLI_p30592	AZL_a09950	AZOBR_180210	AZCBG_490104
AZOLI_p30594	AZL_a09930	AZOBR_p130076	AZCBG_p160452
AZOLI_p40002	AZL_d00950	AZOBR_p1100026	AZCBG_p1110038
AZOLI_p40004	AZL_d00970	AZOBR_p1100023	AZCBG_p1110035
AZOLI_p40037	AZL_d00410	AZOBR_p130073	AZCBG_p160449

AZOLI_p40038	AZL_d00400	AZOBR_p130074	AZCBG_p160450
AZOLI_p40039	AZL_d00390	AZOBR_p130075	AZCBG_p160451
AZOLI_p40052	AZL_d04580	AZOBR_150073	AZCBG_410030
AZOLI_p40059	AZL_d04630	AZOBR_100225	AZCBG_300093
AZOLI_p40061	AZL_d04650	AZOBR_100224	AZCBG_300092
AZOLI_p40062	AZL_d04660	AZOBR_100223	AZCBG_300091
AZOLI_p40064	AZL_d04720	AZOBR_p170135	AZCBG_p1100054
AZOLI_p40065	AZL_d04730	AZOBR_p460001	AZCBG_p4160006
AZOLI_p40074	AZL_d04800	AZOBR_p210025	AZCBG_p220010
AZOLI_p40082	AZL_d04880	AZOBR_150175	AZCBG_420091
AZOLI_p40084	AZL_d04900	AZOBR_10430	AZCBG_90001
AZOLI_p40085	AZL_d04910	AZOBR_10429	AZCBG_80058
AZOLI_p40086	AZL_d04920	AZOBR_10428	AZCBG_80057
AZOLI_p40087	AZL_d04930	AZOBR_10427	AZCBG_80056
AZOLI_p40091	AZL_d04950	AZOBR_10437	AZCBG_100005
AZOLI_p40095	AZL_d04990	AZOBR_10432	AZCBG_90003
AZOLI_p40100	AZL_d05050	AZOBR_p170153	AZCBG_p1100070
AZOLI_p40102	AZL_d05070	AZOBR_p170155	AZCBG_p1100072
AZOLI_p40107	AZL_d05110	AZOBR_p410075	AZCBG_p440035
AZOLI_p40115	AZL_d05190	AZOBR_p460008	AZCBG_p4160012
AZOLI_p40121	AZL_d00040	AZOBR_p150013	AZCBG_p150051
AZOLI_p40126	AZL_d00090	AZOBR_p1130043	AZCBG_p150078
AZOLI_p40127	AZL_d00100	AZOBR_p1130042	AZCBG_p150077
AZOLI_p40137	AZL_d00160	AZOBR_p150038	AZCBG_p150032
AZOLI_p40138	AZL_d00170	AZOBR_p150037	AZCBG_p150033
AZOLI_p40139	AZL_d00180	AZOBR_p150036	AZCBG_p150034
AZOLI_p40140	AZL_pD0023	AZOBR_p150035	AZCBG_p150035
AZOLI_p40150	AZL_d00250	AZOBR_p110148	AZCBG_p160316
AZOLI_p40160	AZL_d00290	AZOBR_p1130011	AZCBG_p140099
AZOLI_p40191	AZL_e02740	AZOBR_110076	AZCBG_330070
AZOLI_p40206	AZL_pD0114	AZOBR_p460068	AZCBG_p4180011
AZOLI_p40209	AZL_d01120	AZOBR_40392	AZCBG_210007
AZOLI_p40212	AZL_d01080	AZOBR_p150030	AZCBG_p150039
AZOLI_p40213	AZL_d01070	AZOBR_p410085	AZCBG_p440046
AZOLI_p40219	AZL_d01150	AZOBR_p140085	AZCBG_p190004
AZOLI_p40220	AZL_d01160	AZOBR_p1130127	AZCBG_p150151
AZOLI_p40230	AZL_d01230	AZOBR_p410050	AZCBG_p440009
AZOLI_p40236	AZL_d03400	AZOBR_p140044	AZCBG_p180018
AZOLI_p40272	AZL_d01670	AZOBR_p130202	AZCBG_p160588
AZOLI_p40274	AZL_pD0184	AZOBR_p130201	AZCBG_p160587
AZOLI_p40275	AZL_pD0185	AZOBR_p130199	AZCBG_p160585
AZOLI_p40276	AZL_d01680	AZOBR_p110144	AZCBG_p160312
AZOLI_p40280	AZL_d01700	AZOBR_p110154	AZCBG_p160322
AZOLI_p40281	AZL_d01710	AZOBR_100185	AZCBG_300054
AZOLI_p40286	AZL_d02930	AZOBR_p170015	AZCBG_p1100018
AZOLI_p40287	AZL_d02920	AZOBR_p170014	AZCBG_p1100017
AZOLI_p40289	AZL_d02910	AZOBR_p170012	AZCBG_p1100015
AZOLI_p40301	AZL_d02860	AZOBR_180224	AZCBG_490117
AZOLI_p40302	AZL_d02850	AZOBR_180223	AZCBG_490115
AZOLI_p40308	AZL_pD0311	AZOBR_p280052	AZCBG_p210009
AZOLI_p40309	AZL_d02810	AZOBR_p250005	AZCBG_p210079
AZOLI_p40311	AZL_d02170	AZOBR_130035	AZCBG_330213
AZOLI_p40321	AZL_d02240	AZOBR_20005	AZCBG_110028
AZOLI_p40327	AZL_d02310	AZOBR_p1130128	AZCBG_p150152
AZOLI_p40330	AZL_d02340	AZOBR_p1130124	AZCBG_p150148
AZOLI_p40333	AZL_d01960	AZOBR_p1110085	AZCBG_p130080
AZOLI_p40336	AZL_d01990	AZOBR_p1100021	AZCBG_p1110033
AZOLI_p40345	AZL_d02070	AZOBR_p110156	AZCBG_p160324
AZOLI_p40346	AZL_d02080	AZOBR_p110155	AZCBG_p160323

AZOLI_p40349	AZL_d02100	AZOBR_p1140114	AZCBG_p150239
AZOLI_p40350	AZL_d02110	AZOBR_p220088	AZCBG_p2100015
AZOLI_p40351	AZL_d02120	AZOBR_p280136	AZCBG_730016
AZOLI_p40352	AZL_d02130	AZOBR_p1150021	AZCBG_p150280
AZOLI_p40353	AZL_d02140	AZOBR_p440052	AZCBG_p480036
AZOLI_p40356	AZL_d02150	AZOBR_p1130154	AZCBG_p150180
AZOLI_p40358	AZL_d02790	AZOBR_p280043	AZCBG_p210017
AZOLI_p40362	AZL_d02750	AZOBR_100168	AZCBG_300037
AZOLI_p40368	AZL_d02670	AZOBR_p1100097	AZCBG_p120016
AZOLI_p40371	AZL_027950	AZOBR_p430064	AZCBG_p2210004
AZOLI_p40373	AZL_d02600	AZOBR_40169	AZCBG_160118
AZOLI_p40374	AZL_d02590	AZOBR_40168	AZCBG_160117
AZOLI_p40375	AZL_d02580	AZOBR_40167	AZCBG_160116
AZOLI_p40376	AZL_d02570	AZOBR_40166	AZCBG_160115
AZOLI_p40377	AZL_d02560	AZOBR_40162	AZCBG_160114
AZOLI_p40382	AZL_d02550	AZOBR_40160	AZCBG_160112
AZOLI_p40383	AZL_d02540	AZOBR_40159	AZCBG_160111
AZOLI_p40385	AZL_d02530	AZOBR_40158	AZCBG_160110
AZOLI_p40388	AZL_d02500	AZOBR_40155	AZCBG_160107
AZOLI_p40389	AZL_d02490	AZOBR_40154	AZCBG_160106
AZOLI_p40396	AZL_d02400	AZOBR_p1110120	AZCBG_p130120
AZOLI_p40403	AZL_d02360	AZOBR_p170021	AZCBG_p1100024
AZOLI_p40420	AZL_d01830	AZOBR_p220081	AZCBG_p2100008
AZOLI_p40426	AZL_d01790	AZOBR_p220080	AZCBG_p2100007
AZOLI_p40428	AZL_d01770	AZOBR_p470004	AZCBG_p4190002
AZOLI_p40431	AZL_d01740	AZOBR_100269	AZCBG_310004
AZOLI_p40434	AZL_d02950	AZOBR_p1130083	AZCBG_p150114
AZOLI_p40436	AZL_d02970	AZOBR_p1130080	AZCBG_p150112
AZOLI_p40439	AZL_d03000	AZOBR_40202	AZCBG_160148
AZOLI_p40441	AZL_d03020	AZOBR_p1130076	AZCBG_p150108
AZOLI_p40442	AZL_d03030	AZOBR_p1130075	AZCBG_p150107
AZOLI_p40443	AZL_d03040	AZOBR_p1130074	AZCBG_p150106
AZOLI_p40446	AZL_d03060	AZOBR_p1130071	AZCBG_p150103
AZOLI_p40447	AZL_d03070	AZOBR_p1130070	AZCBG_p150102
AZOLI_p40451	AZL_d03080	AZOBR_p1170083	AZCBG_p160139
AZOLI_p40453	AZL_d03100	AZOBR_p1170080	AZCBG_p160136
AZOLI_p40460	AZL_d03140	AZOBR_200229	AZCBG_10057
AZOLI_p40461	AZL_013620	AZOBR_140302	AZCBG_370097
AZOLI_p40463	AZL_013610	AZOBR_100105	AZCBG_290009
AZOLI_p40464	AZL_013600	AZOBR_100103	AZCBG_290007
AZOLI_p40466	AZL_013580	AZOBR_100099	AZCBG_290004
AZOLI_p40467	AZL_013570	AZOBR_100227	AZCBG_300095
AZOLI_p40470	AZL_013540	AZOBR_p130016	AZCBG_p160394
AZOLI_p40471	AZL_013530	AZOBR_p130017	AZCBG_p160395
AZOLI_p40473	AZL_013510	AZOBR_140293	AZCBG_370090
AZOLI_p40475	AZL_013500	AZOBR_140292	AZCBG_370089
AZOLI_p40477	AZL_013480	AZOBR_140288	AZCBG_370086
AZOLI_p40478	AZL_013470	AZOBR_140287	AZCBG_370085
AZOLI_p40479	AZL_013460	AZOBR_140286	AZCBG_370084
AZOLI_p40482	AZL_013430	AZOBR_p1130068	AZCBG_p150100
AZOLI_p40494	AZL_d01430	AZOBR_p450011	AZCBG_p4150008
AZOLI_p40502	AZL_d01350	AZOBR_p410052	AZCBG_p440011
AZOLI_p40513	AZL_d03450	AZOBR_p280111	AZCBG_p2210048
AZOLI_p40516	AZL_d03480	AZOBR_p280108	AZCBG_p2210051
AZOLI_p40519	AZL_d03510	AZOBR_p280104	AZCBG_p2210055
AZOLI_p40523	AZL_a01150	AZOBR_p1120012	AZCBG_p140030
AZOLI_p40524	AZL_a01160	AZOBR_p1120013	AZCBG_p140031
AZOLI_p40525	AZL_pD0395	AZOBR_p1120017	AZCBG_p140035
AZOLI_p40532	AZL_d03620	AZOBR_p1120023	AZCBG_p140040

AZOLI_p40536	AZL_d03650	AZOBR_p1120024	AZCBG_p140041
AZOLI_p40610	AZL_d00670	AZOBR_p210081	AZCBG_p240007
AZOLI_p40614	AZL_013410	AZOBR_140271	AZCBG_370070
AZOLI_p40615	AZL_013400	AZOBR_140270	AZCBG_370069
AZOLI_p40619	AZL_013380	AZOBR_100083	AZCBG_280001
AZOLI_p40624	AZL_013340	AZOBR_100079	AZCBG_270089
AZOLI_p40625	AZL_d03160	AZOBR_100125	AZCBG_300001
AZOLI_p40629	AZL_d03180	AZOBR_100123	AZCBG_290026
AZOLI_p40630	AZL_d03190	AZOBR_100122	AZCBG_290025
AZOLI_p40632	AZL_d00700	AZOBR_10506	AZCBG_100077
AZOLI_p40646	AZL_d00810	AZOBR_10535	AZCBG_110020
AZOLI_p40648	AZL_d00830	AZOBR_10532	AZCBG_110018
AZOLI_p40651	AZL_d00850	AZOBR_10528	AZCBG_110014
AZOLI_p40654	AZL_d00870	AZOBR_p1130164	AZCBG_p150191
AZOLI_p50005	AZL_e00720	AZOBR_p410074	AZCBG_p440034
AZOLI_p50006	AZL_e00730	AZOBR_p410092	AZCBG_p450001
AZOLI_p50008	AZL_e00750	AZOBR_p410094	AZCBG_p450003
AZOLI_p50009	AZL_e00760	AZOBR_p410095	AZCBG_p450004
AZOLI_p50010	AZL_e00770	AZOBR_p410096	AZCBG_p450005
AZOLI_p50011	AZL_e00780	AZOBR_p410097	AZCBG_p450006
AZOLI_p50012	AZL_e00790	AZOBR_p470081	AZCBG_p4230030
AZOLI_p50015	AZL_e00820	AZOBR_p470076	AZCBG_p4230026
AZOLI_p50016	AZL_e00830	AZOBR_p470075	AZCBG_p4230025
AZOLI_p50018	AZL_e00850	AZOBR_p470073	AZCBG_p4230023
AZOLI_p50020	AZL_e00870	AZOBR_p470062	AZCBG_p4230014
AZOLI_p50024	AZL_e00910	AZOBR_p470059	AZCBG_p4230010
AZOLI_p50029	AZL_e00960	AZOBR_p470054	AZCBG_p4230005
AZOLI_p50031	AZL_e00980	AZOBR_p470052	AZCBG_p4230003
AZOLI_p50036	AZL_e01020	AZOBR_p410056	AZCBG_p440016
AZOLI_p50037	AZL_e01030	AZOBR_p410057	AZCBG_p440017
AZOLI_p50038	AZL_e01040	AZOBR_p410058	AZCBG_p440018
AZOLI_p50040	AZL_e01050	AZOBR_p410060	AZCBG_p440020
AZOLI_p50041	AZL_e01060	AZOBR_p410061	AZCBG_p440021
AZOLI_p50043	AZL_e01090	AZOBR_p410064	AZCBG_p440024
AZOLI_p50044	AZL_e01100	AZOBR_p410065	AZCBG_p440025
AZOLI_p50045	AZL_e01110	AZOBR_p410066	AZCBG_p440026
AZOLI_p50046	AZL_e01120	AZOBR_p410067	AZCBG_p440027
AZOLI_p50048	AZL_e01140	AZOBR_p410069	AZCBG_p440029
AZOLI_p50049	AZL_e01150	AZOBR_p410070	AZCBG_p440030
AZOLI_p50057	AZL_e01210	AZOBR_p410071	AZCBG_p440031
AZOLI_p50058	AZL_e01220	AZOBR_p410072	AZCBG_p440032
AZOLI_p50059	AZL_e01230	AZOBR_p410073	AZCBG_p440033
AZOLI_p50060	AZL_e01240	AZOBR_100215	AZCBG_300083
AZOLI_p50061	AZL_e01250	AZOBR_100216	AZCBG_300084
AZOLI_p50062	AZL_e01260	AZOBR_100217	AZCBG_300085
AZOLI_p50067	AZL_e03260	AZOBR_p410086	AZCBG_p440047
AZOLI_p50071	AZL_e03240	AZOBR_p1100067	AZCBG_p110005
AZOLI_p50072	AZL_e03230	AZOBR_p230095	AZCBG_p2120033
AZOLI_p50092	AZL_e02330	AZOBR_p280114	AZCBG_p2210045
AZOLI_p50093	AZL_e02320	AZOBR_p280115	AZCBG_p2210044
AZOLI_p50094	AZL_e02310	AZOBR_p280116	AZCBG_p2210043
AZOLI_p50103	AZL_e02220	AZOBR_p230050	AZCBG_p2110077
AZOLI_p50104	AZL_e02210	AZOBR_p450005	AZCBG_p4150003
AZOLI_p50109	AZL_e02160	AZOBR_p450004	AZCBG_p4150002
AZOLI_p50152	AZL_e04000	AZOBR_p130071	AZCBG_p160447
AZOLI_p50156	AZL_e02660	AZOBR_p1130152	AZCBG_p150177
AZOLI_p50158	AZL_e02680	AZOBR_110083	AZCBG_330078
AZOLI_p50160	AZL_e02700	AZOBR_110082	AZCBG_330077
AZOLI_p50162	AZL_e02710	AZOBR_110079	AZCBG_330073

AZOLI_p50163	AZL_e02720	AZOBR_110078	AZCBG_330072
AZOLI_p50164	AZL_e02730	AZOBR_110077	AZCBG_330071
AZOLI_p50166	AZL_e02750	AZOBR_110075	AZCBG_330069
AZOLI_p50171	AZL_e02810	AZOBR_110068	AZCBG_330063
AZOLI_p50172	AZL_e02820	AZOBR_110067	AZCBG_330062
AZOLI_p50173	AZL_e02830	AZOBR_110066	AZCBG_330061
AZOLI_p50177	AZL_e02900	AZOBR_100173	AZCBG_300043
AZOLI_p50178	AZL_e02910	AZOBR_100171	AZCBG_300040
AZOLI_p50180	AZL_e02920	AZOBR_100169	AZCBG_300038
AZOLI_p50184	AZL_e02950	AZOBR_p280017	AZCBG_p210041
AZOLI_p50186	AZL_e02980	AZOBR_10483	AZCBG_100053
AZOLI_p50190	AZL_e03010	AZOBR_p280095	AZCBG_p2210063
AZOLI_p50191	AZL_e03020	AZOBR_p280093	AZCBG_p2210064
AZOLI_p50193	AZL_e03040	AZOBR_p280091	AZCBG_p2210066
AZOLI_p50194	AZL_e03050	AZOBR_p280090	AZCBG_p2210067
AZOLI_p50195	AZL_e03060	AZOBR_p280089	AZCBG_p2210068
AZOLI_p50196	AZL_e03070	AZOBR_p280088	AZCBG_p2210069
AZOLI_p50197	AZL_e03080	AZOBR_p280087	AZCBG_p2210070
AZOLI_p50201	AZL_e03110	AZOBR_p1100103	AZCBG_p120021
AZOLI_p50204	AZL_e03120	AZOBR_p130072	AZCBG_p160448
AZOLI_p50205	AZL_e03130	AZOBR_100214	AZCBG_300082
AZOLI_p50206	AZL_e03140	AZOBR_p110126	AZCBG_p160296
AZOLI_p50210	AZL_e03180	AZOBR_p110131	AZCBG_p160300
AZOLI_p50211	AZL_e03190	AZOBR_p110132	AZCBG_p160301
AZOLI_p50213	AZL_e02480	AZOBR_p1130156	AZCBG_p150183
AZOLI_p50215	AZL_e02490	AZOBR_p1130157	AZCBG_p150184
AZOLI_p50218	AZL_e02500	AZOBR_p150084	AZCBG_p140109
AZOLI_p50222	AZL_e02540	AZOBR_p440122	AZCBG_p4100010
AZOLI_p50223	AZL_e02550	AZOBR_p440123	AZCBG_p4100011
AZOLI_p50225	AZL_e02570	AZOBR_p440126	AZCBG_p4100014
AZOLI_p50226	AZL_e02580	AZOBR_p440127	AZCBG_p4100015
AZOLI_p50228	AZL_e02650	AZOBR_100218	AZCBG_300086
AZOLI_p50234	AZL_e03780	AZOBR_p150028	AZCBG_p150041
AZOLI_p50235	AZL_e03790	AZOBR_p280099	AZCBG_p2210059
AZOLI_p50239	AZL_e03820	AZOBR_p270227	AZCBG_p2200023
AZOLI_p50240	AZL_e03840	AZOBR_p270225	AZCBG_p2200020
AZOLI_p50241	AZL_e03850	AZOBR_p270224	AZCBG_p2200019
AZOLI_p50243	AZL_e03870	AZOBR_p270222	AZCBG_p2200017
AZOLI_p50244	AZL_e03880	AZOBR_110023	AZCBG_330021
AZOLI_p50247	AZL_e02150	AZOBR_p210002	AZCBG_730009
AZOLI_p50249	AZL_e02130	AZOBR_p150079	AZCBG_p140113
AZOLI_p50250	AZL_e02120	AZOBR_p150080	AZCBG_p140112
AZOLI_p50251	AZL_e02110	AZOBR_p150081	AZCBG_p140111
AZOLI_p50254	AZL_e02040	AZOBR_p1160014	AZCBG_p160015
AZOLI_p50255	AZL_e02030	AZOBR_p1160015	AZCBG_p160016
AZOLI_p50256	AZL_e02020	AZOBR_p1130151	AZCBG_p150176
AZOLI_p50259	AZL_e01990	AZOBR_p130186	AZCBG_p160570
AZOLI_p50260	AZL_e01980	AZOBR_p1160023	AZCBG_p160022
AZOLI_p50261	AZL_e01970	AZOBR_p1160022	AZCBG_p160021
AZOLI_p50262	AZL_e01960	AZOBR_p1160021	AZCBG_p160020
AZOLI_p50263	AZL_e01950	AZOBR_p1160020	AZCBG_p160019
AZOLI_p50269	AZL_e01840	AZOBR_p1110013	AZCBG_p130011
AZOLI_p50272	AZL_e01830	AZOBR_100210	AZCBG_300078
AZOLI_p50273	AZL_e01820	AZOBR_100209	AZCBG_300077
AZOLI_p50276	AZL_e01800	AZOBR_100207	AZCBG_300075
AZOLI_p50279	AZL_pE0217	AZOBR_100202	AZCBG_300070
AZOLI_p50282	AZL_e01740	AZOBR_150149	AZCBG_420067
AZOLI_p50283	AZL_e01730	AZOBR_110099	AZCBG_330095
AZOLI_p50284	AZL_e01720	AZOBR_110098	AZCBG_330094

AZOLI_p50285	AZL_e01710	AZOBR_110097	AZCBG_330093
AZOLI_p50286	AZL_e01700	AZOBR_110096	AZCBG_330092
AZOLI_p50295	AZL_e01630	AZOBR_p140078	AZCBG_p180050
AZOLI_p50296	AZL_e01620	AZOBR_p140079	AZCBG_p180051
AZOLI_p50297	AZL_e01610	AZOBR_p140080	AZCBG_p180052
AZOLI_p50300	AZL_e01590	AZOBR_p140082	AZCBG_p190001
AZOLI_p50301	AZL_e01580	AZOBR_p350001	AZCBG_p190002
AZOLI_p50304	AZL_pE0191	AZOBR_p1100092	AZCBG_p120012
AZOLI_p50307	AZL_e01530	AZOBR_p1100102	AZCBG_p120020
AZOLI_p50309	AZL_e01490	AZOBR_p270141	AZCBG_p2190015
AZOLI_p50310	AZL_e01480	AZOBR_p270139	AZCBG_p2190014
AZOLI_p50311	AZL_e01470	AZOBR_p270138	AZCBG_p2190013
AZOLI_p50312	AZL_e01460	AZOBR_p270137	AZCBG_p2190012
AZOLI_p50315	AZL_e01430	AZOBR_p440076	AZCBG_p490019
AZOLI_p50316	AZL_e01420	AZOBR_p280131	AZCBG_730019
AZOLI_p50317	AZL_e01410	AZOBR_p280124	AZCBG_p2210038
AZOLI_p50318	AZL_e01400	AZOBR_p280123	AZCBG_p2210039
AZOLI_p50320	AZL_e01390	AZOBR_p130081	AZCBG_p160456
AZOLI_p50322	AZL_e01380	AZOBR_p230016	AZCBG_p2110044
AZOLI_p50323	AZL_e01370	AZOBR_p1130176	AZCBG_p150202
AZOLI_p50335	AZL_e01340	AZOBR_p220019	AZCBG_p280033
AZOLI_p50336	AZL_e01330	AZOBR_p220018	AZCBG_p280032
AZOLI_p50337	AZL_e01320	AZOBR_p220017	AZCBG_p280031
AZOLI_p50338	AZL_e01310	AZOBR_p220016	AZCBG_p280030
AZOLI_p50339	AZL_e01300	AZOBR_p220015	AZCBG_p280029
AZOLI_p50340	AZL_e01290	AZOBR_p220014	AZCBG_p280028
AZOLI_p50341	AZL_e01280	AZOBR_p220013	AZCBG_p280027
AZOLI_p50342	AZL_e01270	AZOBR_p220012	AZCBG_p280026
AZOLI_p50355	AZL_e03420	AZOBR_p410076	AZCBG_p440036
AZOLI_p50356	AZL_e03460	AZOBR_p1100079	AZCBG_p120003
AZOLI_p50359	AZL_e03490	AZOBR_p1100076	AZCBG_p110016
AZOLI_p50360	AZL_e03500	AZOBR_p1100075	AZCBG_p110015
AZOLI_p50362	AZL_e03510	AZOBR_p1100074	AZCBG_p110014
AZOLI_p50364	AZL_e03520	AZOBR_p1100073	AZCBG_p110013
AZOLI_p50365	AZL_e03530	AZOBR_p1100072	AZCBG_p110012
AZOLI_p50366	AZL_e03540	AZOBR_100400	AZCBG_320095
AZOLI_p50371	AZL_e03570	AZOBR_p440155	AZCBG_p4110019
AZOLI_p50372	AZL_e03580	AZOBR_p440156	AZCBG_p4110020
AZOLI_p50373	AZL_e03590	AZOBR_p440157	AZCBG_p4110021
AZOLI_p50374	AZL_e03600	AZOBR_p440158	AZCBG_p4110022
AZOLI_p50377	AZL_e03630	AZOBR_p440160	AZCBG_p4110024
AZOLI_p50378	AZL_e03640	AZOBR_p440161	AZCBG_p4110025
AZOLI_p50379	AZL_e03650	AZOBR_p440162	AZCBG_p4110026
AZOLI_p50380	AZL_e03660	AZOBR_p440163	AZCBG_p4110027
AZOLI_p50381	AZL_e03670	AZOBR_p440164	AZCBG_p4120001
AZOLI_p50383	AZL_e03690	AZOBR_p440166	AZCBG_p4120003
AZOLI_p50384	AZL_e03700	AZOBR_p440167	AZCBG_p4120004
AZOLI_p50389	AZL_e04030	AZOBR_p210148	AZCBG_p260019
AZOLI_p50393	AZL_e00110	AZOBR_p420021	AZCBG_p460009
AZOLI_p50394	AZL_e00100	AZOBR_p420020	AZCBG_p460010
AZOLI_p50404	AZL_e00060	AZOBR_p130024	AZCBG_p160401
AZOLI_p50406	AZL_e00050	AZOBR_p130027	AZCBG_p160403
AZOLI_p50407	AZL_e00030	AZOBR_p470028	AZCBG_p4200010
AZOLI_p50408	AZL_e00020	AZOBR_p220109	AZCBG_p2110017
AZOLI_p50414	AZL_e04150	AZOBR_p1100065	AZCBG_p110003
AZOLI_p50415	AZL_e04140	AZOBR_p1100066	AZCBG_p110004
AZOLI_p50419	AZL_e04090	AZOBR_p210027	AZCBG_p220012
AZOLI_p50421	AZL_e04060	AZOBR_p210077	AZCBG_p240002
AZOLI_p50422	AZL_e04050	AZOBR_p210076	AZCBG_p240001

AZOLI_p50424	AZL_d02680	AZOBR_p440177	AZCBG_p4130007
AZOLI_p50426	AZL_d02690	AZOBR_p440178	AZCBG_p4130008
AZOLI_p50427	AZL_d02700	AZOBR_p440179	AZCBG_p4130009
AZOLI_p50432	AZL_e04040	AZOBR_p210075	AZCBG_p230012
AZOLI_p50438	AZL_e00120	AZOBR_30022	AZCBG_140017
AZOLI_p60001	AZL_f01000	AZOBR_p170007	AZCBG_p1100011
AZOLI_p60004	AZL_f00990	AZOBR_p160006	AZCBG_p1100004
AZOLI_p60007	AZL_f01640	AZOBR_p60012	AZCBG_p60020
AZOLI_p60009	AZL_f01630	AZOBR_p160005	AZCBG_p1100003
AZOLI_p60011	AZL_f01580	AZOBR_p60010	AZCBG_p60023
AZOLI_p60035	AZL_002040	AZOBR_40062	AZCBG_160025
AZOLI_p60044	AZL_f01610	AZOBR_p160008	AZCBG_p1100006
AZOLI_p60059	AZL_f01200	AZOBR_p270153	AZCBG_p2200005
AZOLI_p60062	AZL_f01320	AZOBR_p270152	AZCBG_p2200004
AZOLI_p60101	AZL_f00340	AZOBR_p1160029	AZCBG_p160029
AZOLI_p60105	AZL_f00310	AZOBR_p1160031	AZCBG_p160031
AZOLI_p60109	AZL_f00270	AZOBR_p1160030	AZCBG_p160030
AZOLI_p60110	AZL_f00260	AZOBR_150085	AZCBG_420008
AZOLI_p60158	AZL_f00160	AZOBR_p60034	AZCBG_p60055
AZOLI_p60164	AZL_f00120	AZOBR_p1170036	AZCBG_p160094
AZOLI_p60168	AZL_f00080	AZOBR_p60067	AZCBG_p60096
AZOLI_p60173	AZL_f00050	AZOBR_p60065	AZCBG_p60094
AZOLI_p60177	AZL_f00010	AZOBR_p60077	AZCBG_p60106
AZOLI_p60182	AZL_f01870	AZOBR_p60091	AZCBG_p60119
AZOLI_p60192	AZL_f01820	AZOBR_p60078	AZCBG_p60107
AZOLI_p60194	AZL_f01830	AZOBR_p60033	AZCBG_p60051
AZOLI_p60195	AZL_f01810	AZOBR_p60007	AZCBG_p60025
AZOLI_p60196	AZL_f01800	AZOBR_p60006	AZCBG_p60026
AZOLI_p60197	AZL_f01790	AZOBR_p60005	AZCBG_p60027
AZOLI_p60230	AZL_f01740	AZOBR_p160004	AZCBG_p1100002
AZOLI_p60265	AZL_f01050	AZOBR_p60001	AZCBG_p60017
