# Supplementary material for: Genome Sequence of Azospirillum brasilense CBG497 and Comparative Analyses of Azospirillum Core and Accessory Genomes provide Insight into Niche Adaptation
Source: Genes (Basel). 2012 Sep 28;3(4):576–602. doi: 10.3390/genes3040576 (PMC3899980; doi:10.3390/genes3040576)

Supplementary Materials

Figure S1. Phylogenetic relationship between *Azospirillum* strains. A 16S rRNA maximum likelihood tree was constructed using the following sequences downloaded from EMBL: *Azospirillum largomobile* ACM2041 (X90759), *A. melinis* LMG24250 (GU256442), *A. lipoferum* Sp59b (GU256441), *A. oryzae* COC8 (GU256443), *A. zeae* strain N7 (DQ682470), *A. doebereinerae* GSF71 (AJ238567), *A. thiophilum* BV-s (EU678791), *A. picis* IMMIB TAR-3T (AM922283), *A. rugosum* IMMIB AFH-6T (AM419042), *A. canadense* DS2 (HM636056), *A. formosense* CC-Nfb-7 (GU256444), *A. brasilense* Sp7 (GU256438), *A. halopraeferens* Au4 (GU256439), *A. amazonense* Y1 (GU256437), *A. irakense* KBC1 (GU256440), *A. palatum* ww 10 (EU747318), *Azospirillum* sp. B510 (AB049111), *Rhodospirillum centenum* SW (NC_011420) and *Azorhizobium caulinodans* NBRC14845 (AB680677).

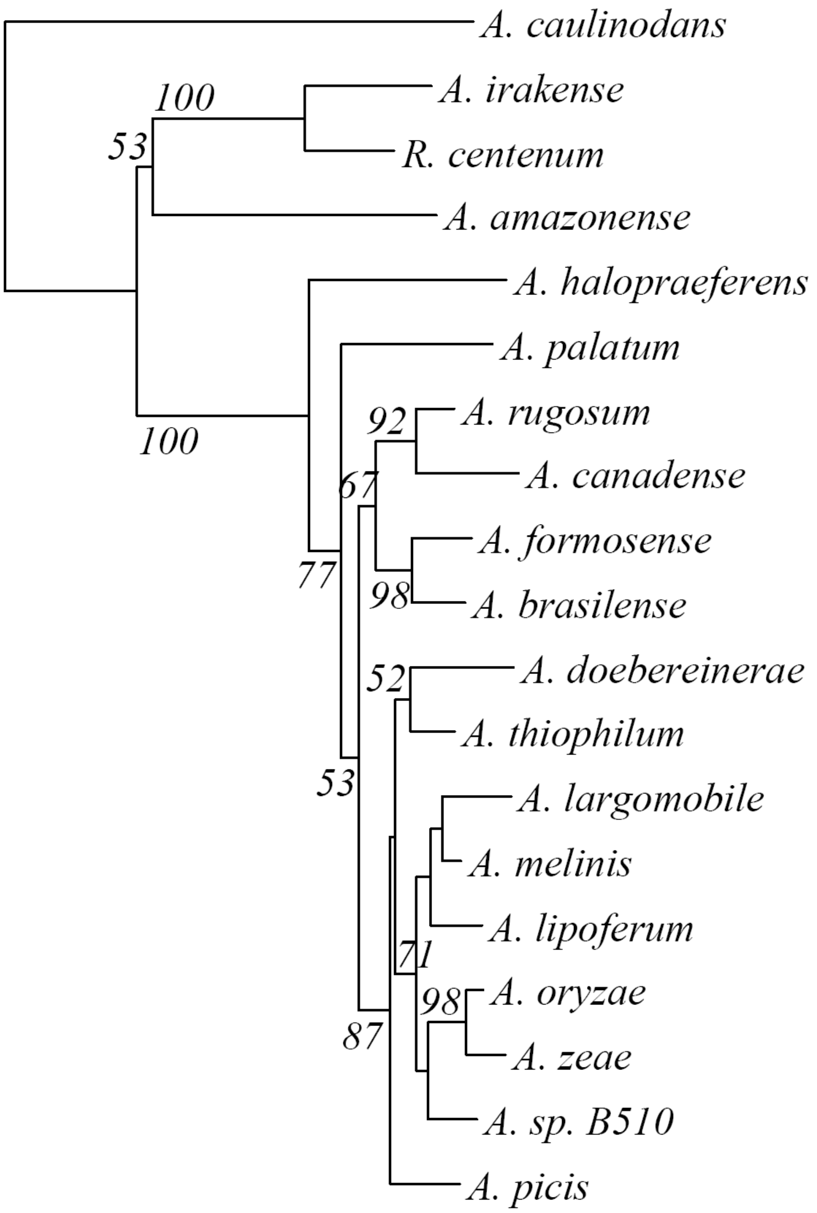

Figure S2. Growth on phenylacetate. *Azospirillum* strains were grown in AB medium containing 0.1% phenyacetate as sole carbon source. (●) *A. lipoferum* 4B; (○) *A. brasilense* Sp245; (▼) *A. brasilense* CBG497.

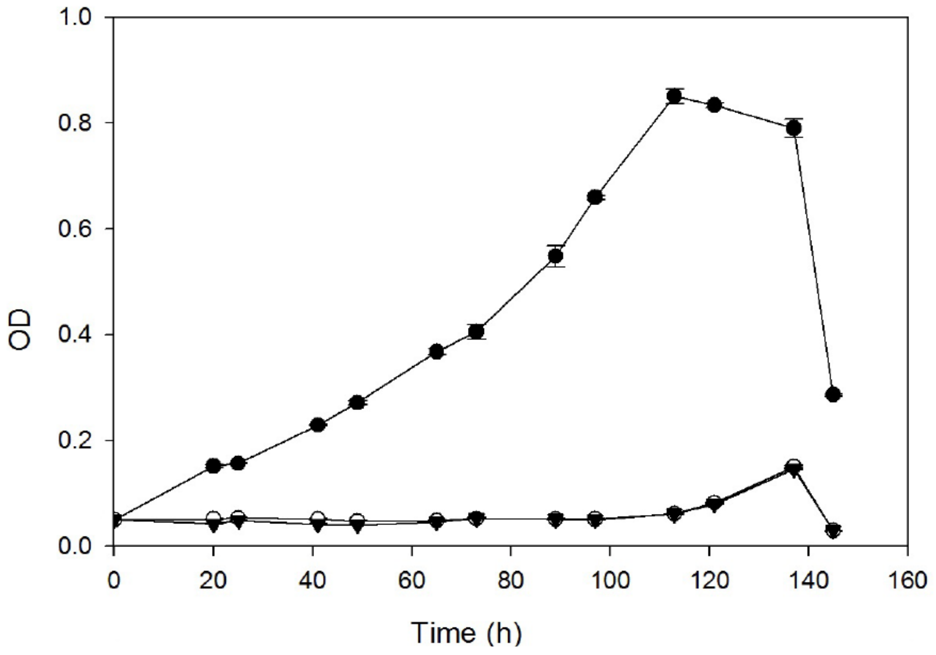

Supplement: Supplementary File 2 — Supplementary Figures (PDF, 156 KB) [file genes-03-00576-s002.pdf]
